# Genomics of chronic dry cough unravels neurological pathways

**DOI:** 10.1183/13993003.02341-2024

**Published:** 2025-09-25

**Authors:** Kayesha Coley, Catherine John, Jonas Ghouse, David J. Shepherd, Nick Shrine, Abril G. Izquierdo, Stavroula Kanoni, Emma F. Magavern, Richard Packer, Lorcan McGarvey, Jaclyn A. Smith, Henning Bundgaard, Sisse R. Ostrowski, Christian Erikstrup, Ole B.V. Pedersen, David A. van Heel, Genes and Health Research Team, William Hennah, Mikko Marttila, Robert C. Free, Edward J. Hollox, Louise V. Wain, Martin D. Tobin, Chiara Batini

**Affiliations:** 1 Department of Population Health Sciences, University of Leicester, Leicester, UK; 2 University Hospitals of Leicester NHS Trust, Leicester, UK; 3 Laboratory for Molecular Cardiology, Department of Cardiology, Copenhagen University Hospital, Rigshospitalet, Copenhagen, Denmark; 4 Laboratory for Molecular Cardiology, Department of Biomedical Sciences, University of Copenhagen, Copenhagen, Denmark; 5 William Harvey Research Institute, Barts and the London School of Medicine and Dentistry, Queen Mary University of London, London, UK; 6 Wellcome-Wolfson Institute for Experimental Medicine, School of Medicine, Dentistry and Biomedical Sciences, Queen's University Belfast, Belfast, UK; 7 Division of Immunology, Immunity to Infection and Respiratory Medicine, The University of Manchester, Manchester University NHS Foundation Trust, Manchester, UK; 8 Department of Cardiology, Copenhagen University Hospital, Rigshospitalet, University of Copenhagen, Copenhagen, Denmark; 9 Department of Clinical Medicine, University of Copenhagen, Copenhagen, Denmark; 10 Department of Clinical Immunology, Rigshospitalet, Copenhagen University Hospital, Copenhagen, Denmark; 11 Department of Clinical Immunology, Aarhus University Hospital, Aarhus, Denmark; 12 Department of Clinical Medicine, Aarhus University, Aarhus, Denmark; 13 Department of Clinical Immunology, Zealand University Hospital, Køge, Denmark; 14 Blizard Institute, Barts and the London School of Medicine and Dentistry, Queen Mary University of London, London, UK; 15 Orion Pharma, Espoo, Finland; 16 Neuroscience Center, HiLIFE, University of Helsinki, Helsinki, Finland; 17 Orion Pharma, Nottingham, UK; 18 School of Computing and Mathematical Sciences, University of Leicester, Leicester, UK; 19 Department of Genetics and Genome Biology, University of Leicester, Leicester, UK

## Abstract

**Background:**

Chronic dry cough is a symptom of common lung conditions, can occur as a side-effect of angiotensin-converting enzyme inhibitors (ACEis), or may be unexplained. Despite the substantial health burden presented by chronic dry cough, its biological mechanisms remain unclear. We hypothesised shared genetic architecture between chronic dry cough and ACEi-induced cough and aimed to identify causal genes underlying both phenotypes.

**Methods:**

We performed multi-ancestry genome-wide association studies (GWAS) of chronic dry cough and ACEi-induced cough, and a multi-trait GWAS of both phenotypes, utilising data from five cohort studies. Chronic dry cough was defined by questionnaire responses, and ACEi-induced cough by treatment switches or clinical diagnosis in electronic health records. We mapped putative causal genes and performed phenome-wide association studies (PheWAS) of associated variants, and polygenic scores for ACEi-induced cough, to identify pleiotropic effects.

**Results:**

We found seven novel genetic association signals reaching p<5×10^–8^ in the multi-trait or single-trait analyses of chronic dry cough and ACEi-induced cough. The novel variants mapped to 10 novel genes, and we mapped an additional three novel genes to known risk variants, many of which implicate neurological functions (*CTNNA1*, *KCNA10*, *MAPKAP1*, *OR4C12*, *OR4C13*, *SIL1*). The polygenic-score-based PheWAS highlighted associations with an elevated risk of several clinical conditions including asthma, diabetes and multi-site chronic pain.

**Conclusion:**

Our findings provide support for neuronal dysfunction underlying cough hypersensitivity in chronic dry cough and ACEi-induced cough, and identify diseases and traits associated with genetic predisposition to cough that could inform drug target discovery.

## Shareable abstract

**This study leverages the genetics of two strongly correlated chronic cough traits (chronic dry cough and ACE inhibitor-induced cough). It highlights the contribution of neurobiological processes and provides new evidence for drug development and repurposing.** https://bit.ly/3H0MHxy

## Graphical abstract

**Figure:**
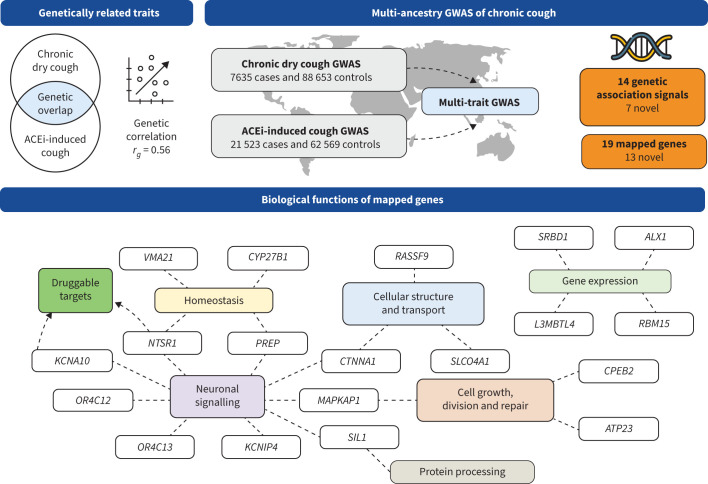
Overview of the study. GWAS: genome-wide association study; ACEi: angiotensin-converting enzyme inhibitor.

## Introduction

Chronic dry cough is common, impairs quality of life and results in substantial healthcare system burden [1]. It may derive from a range of clinical conditions, including chronic lung diseases, gastro-oesophageal reflux and rhinitis, and is also associated with risk factors such as smoking and occupational exposures. It may be a side-effect of medications, including widely used angiotensin-converting enzyme inhibitors (ACEis). In a small proportion of cases, chronic cough is unexplained, with no identifiable comorbidities [2–5]. The term cough hypersensitivity syndrome has been used to describe troublesome coughing triggered by low levels of thermal, mechanical or chemical exposure [6]. Neuronal dysregulation has been postulated to underlie cough hypersensitivity, which is thought to be a distinct treatable trait [7]. Although treatments remain underdeveloped [8], recent studies have demonstrated the efficacy of therapies targeting neuronal mechanisms in patients with features consistent with cough hypersensitivity, diagnosed with refractory or unexplained chronic cough [6, 8, 9].

As genetic variants are assigned by a random process at conception, genetic associations provide understanding about causal relationships that are not subject to the confounding and reverse causation that affects traditional observational epidemiology. Therefore, genetic associations are used to guide mechanistic, functional genomics studies and drug development; the probability of success for drug mechanisms with genetic support is 2.6 times greater than those without [10]. While the genetics of asthma, COPD and chronic sputum production have been well studied [11–13], there has been a lack of genome-wide association studies (GWAS) of chronic dry cough. In this study, we explore two cough phenotypes: chronic dry cough and ACEi-induced cough, which share similar clinical manifestations, including a dry nature and involvement of cough reflex hypersensitivity suggesting a role of the nervous system in their pathophysiology [6, 14, 15]. There have been no GWAS of chronic dry cough and few for ACEi-induced cough [16–19], with findings from the latter implicating neuronal excitability and the bradykinin pathway [16, 19]. Given the significant genetic correlation between ACEi-induced cough (as defined using an ACEi to angiotensin-II receptor blocker (ARB) switch) and “cough on most days” (*r_g_*=0.37) [16], we hypothesised that there would be common genetic determinants of chronic dry cough and ACEi-induced cough. Our study presents the first GWAS dedicated to chronic dry cough, and applies a multi-trait GWAS approach to enhance our discovery power and detect shared associated genetic variants [20].

Therefore, we 1) performed the first GWAS of chronic dry cough; 2) conducted the largest multi-ancestry study of ACEi-induced cough; 3) tested genetic correlation between chronic dry cough and ACEi-induced cough; 4) undertook the first multi-trait GWAS of chronic dry cough and ACEi-induced cough; 5) implemented a consensus-based framework to investigate putative causal genes for these traits; 6) applied phenome-wide association studies (PheWAS) to individual variants and a polygenic score (PGS) to inform understanding of the potential consequences of perturbing pathways involved in chronic cough; and 7) used PGS to assess the corroboration and transferability of findings. Using these approaches, we detected novel genetic associations and identified causal genes and pathways underlying these under-studied cough phenotypes.

## Methods

Please refer to the supplementary material for additional details relating to the following methods.

### Study populations and phenotype definitions

Our study included individuals from UK Biobank [21], the EXCEED Study [22], Genes & Health [23] and Copenhagen Hospital Biobank [16, 24], and summary statistics from a previously published study conducted in the eMERGE Network [19, 25], in discovery. Quality control and imputation of array-based genotypes was performed by each respective cohort study (supplementary methods; supplementary table S1). The All of Us Research Program [26] was used to corroborate associations, leveraging quality-controlled whole sequencing data available on the Research Workbench (supplementary methods) [27].

We defined ACEi-induced cough using electronic health records linked to UK Biobank [21], EXCEED [22], Genes & Health [23], Copenhagen Hospital Biobank [16, 24], and All of Us [26], whereby cases switched from an ACEi to an ARB and controls were continuous users of ACEis. The Electronic Medical Records and Genomics (eMERGE) Network study defined ACEi-induced cough using an algorithm based on prescriptions and the allergy section in electronic health records, which was validated by manual record review [19, 25].

We defined chronic dry cough in UK Biobank [21] using questionnaire data. Among UK Biobank participants who were neither ACEi-induced cough cases or controls, we defined chronic dry cough cases (nonproductive cough on most days) and controls (neither coughed on most days nor brought up sputum on most days).

### Discovery GWAS

In each discovery cohort, we performed ancestry-specific GWAS of chronic dry cough and ACEi-induced cough using imputed genomic variants (imputation quality ≥0.3, and minor allele count ≥10 in cases and controls), and age, age-squared, sex, genotyping array and at least 10 principal components of genetic ancestry as covariates. For chronic dry cough, ever-smoking status was an additional covariate. We also utilised summary statistics from the previously published eMERGE Network study (supplementary table S1) [19].

**FIGURE 1:**
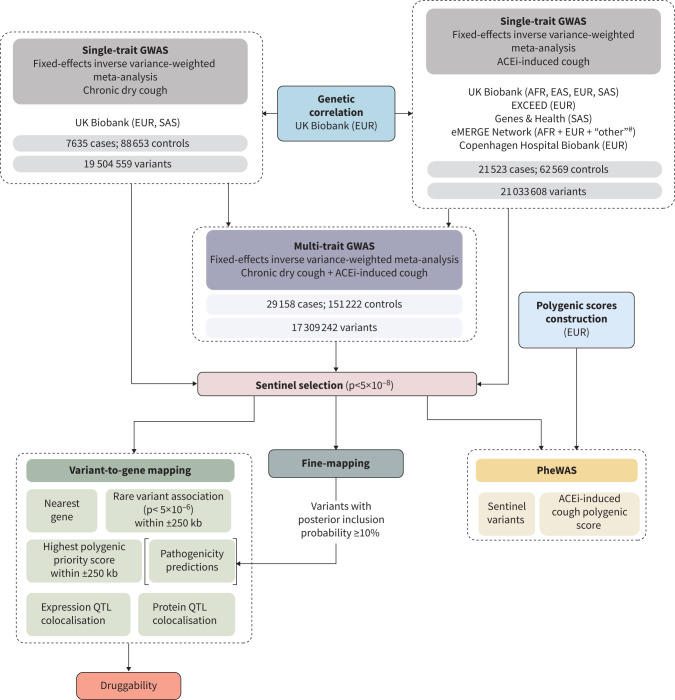
Study design. GWAS: genome-wide association studies; EUR: European; SAS: South Asian; ACEi: angiotensin-converting enzyme inhibitor; AFR: African; EAS: East Asian; PheWAS: phenome-wide association studies; QTL: quantitative trait loci. ^#^: defined by the authors of that study [19].

Using a fixed-effects inverse variance-weighted model [28] we undertook a GWAS meta-analysis for chronic dry cough and a GWAS meta-analysis for ACEi-induced cough. Then we conducted a multi-trait GWAS [20] by meta-analysis of the chronic dry cough GWAS and ACEi-induced cough GWAS using a fixed-effects inverse-variance weighted model (figure 1) [28]. To inform the aspects of our variant-to-gene mapping and the PGS development detailed later, we also performed European (EUR) ancestries-only single-trait and multi-trait GWAS.

In each GWAS meta-analysis, we defined sentinel variants reaching genome-wide significance (p<5×10^–8^) and performed fine-mapping, as described in the supplementary methods. We defined single-trait sentinels as independent from multi-trait sentinels based on a linkage disequilibrium threshold of r^2^<0.1. In addition, we used r^2^<0.1 to define novel multi-trait and single-trait (ACEi-induced cough) sentinels from previously reported associations (p<5×10^–8^) for ACEi-induced cough [16, 18, 19], given the absence of published GWAS of chronic dry cough.

We conducted sex-specific association testing and sex interaction testing in the UK Biobank (EUR only) for chronic dry cough and ACEi-induced cough to assess sex differences among our sentinel variants for each single trait and the combined multi-trait analysis.

### Polygenic scores

As PGS capture the combined effects of many variants and provide considerably more power for association testing than individual variants, we used such scores to 1) test whether we were capturing a similar phenotype in UK Biobank as the extensively validated eMERGE Network study phenotype, by testing the association of the eMERGE-derived PGS for ACEi-induced cough with ACEi-induced cough in UK Biobank; 2) corroborate our collective findings for ACEi-induced cough in UK Biobank in an additional biobank, All of Us, by testing the association of our EUR ancestry discovery (UK Biobank, EXCEED, Copenhagen Hospital Biobank) cohorts-derived PGS for ACEi-induced cough with ACEi-induced cough in All of Us participants of EUR ancestries; 3) assess transferability of findings across different ancestry groups by testing the EUR ancestry derived PGS for ACEi-induced cough in individuals of South Asian (SAS) and African (AFR) ancestries in the Genes & Health, All of Us and UK Biobank populations (supplementary methods).

### Investigating clinical and biological relevance of genetic associations

We mapped genes to all sentinel variants, both novel and previously reported, by integrating the following evidence: 1) nearest gene; 2) nearest gene to a deleterious or damaging variant with a posterior inclusion probability ≥10%; 3) the gene with the highest polygenic priority score [29] within ±250 kb of the sentinel for the relevant trait (multi-trait, chronic dry cough or ACEi-induced cough); 4) exome-wide rare variant association (p<5×10^–6^) with the relevant trait in UK Biobank within ±250 kb of the sentinel; 5) association with gene expression; and 6) association with protein expression. Mapped genes supported by at least one level of evidence were considered putative causal, and we used database (GeneCards [30], Open Targets Platform [31], Online Mendelian Inheritance in Man [32], Drug–Gene Interaction Database [33]) and literature searches to retrieve information about their biological function and clinical relevance.

We used DeepPheWAS [34] to test association between individual sentinels (across multiple ancestries) or PGS (in EUR only) and 1939 phenotypes defined in UK Biobank, adjusting for age, sex, genotyping array and 10 principal components of genetic ancestry. This was complemented by a custom respiratory PheWAS to query variants against the most powerful GWASs performed across 10 clinical respiratory traits (supplementary table S2). We report PheWAS associations at a false discovery rate (FDR) <1%. Additionally, we queried Open Targets Genetics [35] to assess whether sentinels were in linkage disequilibrium (r^2^>0.8) with genome-wide significant lead GWAS variants for other traits.

### Heritability and genetic correlations

We estimated the single-nucleotide polymorphism (SNP) heritability of chronic dry cough and ACEi-induced cough, as well as asthma and COPD for contextualisation, using linkage disequilibrium score regression (LDSC) [36, 37] in EUR individuals. We also estimated the proportion of variance explained by the sentinel variants for chronic dry cough and ACEi-induced cough. Additionally, we used LDSC [38] to assess SNP heritability enrichment across specific tissue types based on gene expression and chromatin accessibility marks. In addition, we used LDSC [36, 37] to calculate the genetic correlation between ACEi-induced cough and chronic dry cough, and between each cough trait and the clinical conditions with significant associations in the ACEi-induced cough PGS-based PheWAS (described later). Further details on heritability and genetic correlation analyses are available in the supplementary methods.

### Sensitivity analyses

We performed three sensitivity analyses: 1) for chronic dry cough, excluding cases who coughed on most days for <1 year (described as “short-term chronic dry cough”); 2) for ACEi-induced cough (in UK Biobank only), excluding cases with a clinical cough code recorded within 12 months of switching to an ARB; and 3) for chronic dry cough and ACEi-induced cough (in UK Biobank only), excluding individuals with asthma. Further details are provided in the supplementary methods.

## Results

The multi-trait GWAS of chronic dry cough and ACEi-induced cough included 29 158 cases and 151 222 controls, comprising 7635 cases and 88 653 controls for chronic dry cough and 21 523 cases and 62 569 controls for ACEi-induced cough (figure 1; supplementary table S3). The SNP heritability±se for chronic dry cough was 3.19±0.53% and for ACEi-induced cough was up to 5.30±1.4%, comparable to that of asthma (2.60±0.2%) and COPD (1.83±0.1%) (supplementary table S4), and there was a strong genetic correlation between chronic dry cough and ACEi-induced cough (*r_g_*=0.56±0.15, p=0.0001). A PGS for ACEi-induced cough derived from the eMERGE Network [19], which extensively clinically validated the ACEi-induced cough phenotype, showed strong association with the ACEi to ARB switch in UK Biobank (p=3.87×10^−4^) (supplementary table S5), indicating that the UK Biobank definition captured the same underlying phenotype.

**FIGURE 2:**
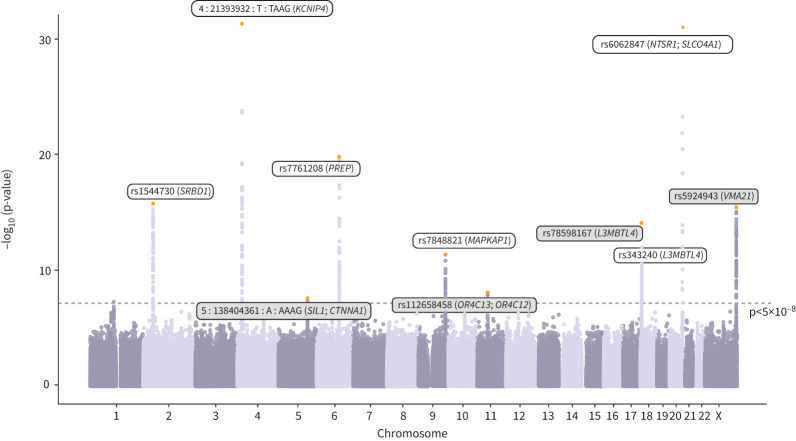
Manhattan plot for the multi-trait genome-wide association study of chronic dry cough and angiotensin-converting enzyme induced cough. The dotted grey line represents the genome-wide significance threshold (p<5×10^–8^). Sentinels are highlighted orange and mapped genes are labelled; grey boxes indicate novel sentinels.

**FIGURE 3:**
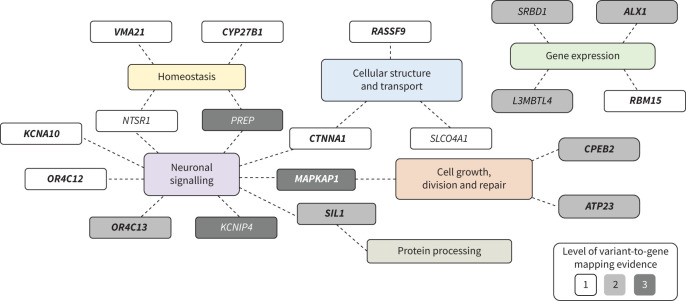
Functional summary of putative causal genes. Based on integration of evidence from multiple sources, including GeneCards [30], Open Targets Platform [31], Online Mendelian Inheritance in Man [32], the Drug–Gene Interaction Database [33] and published literature. Variant-to-gene mapping evidence is provided in supplementary figure S1 and supplementary table S8. Novel genes are presented in bold.

Across the multi-trait and single-trait analyses, we defined 14 independent signals, identified by sentinel variants reaching p<5×10^–8^ (figure 2; supplementary figure S1; supplementary tables S6 and S7). Together, our 14 sentinel variants explain 4.96% and 27.8% of the SNP-heritability of chronic dry cough and ACEi-induced cough, respectively (supplementary table S4). Through our comprehensive variant-to-gene mapping approach, we mapped these signals to 19 putative causal genes (supplementary figure S2; supplementary tables S8–S11) and explored their biological functions (figure 3; descriptions reported in supplementary table S12) and gene–drug interactions (supplementary table S13).

We found seven novel genetic association signals in the multi-trait or single-trait analyses of chronic dry cough and ACEi-induced cough (table 1; supplementary tables S6 and S7). In the multi-trait GWAS, four novel signals mapped to six genes, five of which (*CTNNA1*, *OR4C12*, *OR4C13*, *SIL1* and *VMA21*) have not been previously implicated in any cough GWAS (figure 2). The remaining gene, *L3MBTL4*, was previously implicated by a common variant (rs8097200) associated with ACEi-induced cough [16], and independent from our sentinel, rs78598167 (r^2^=0.003).

**TABLE 1:** Novel sentinel variants (p<5×10^−8^) identified in the multi-trait genome-wide association study (GWAS) of chronic dry cough and angiotensin-converting enzyme (ACEi)-induced cough, and additional novel sentinels from each single-trait GWAS

Sentinel identifier	Position (hg19)	Trait	Effect allele	Effect allele frequency	OR (95% CI)	p-value	Annotation	Mapped gene(s)
**5:138404361:A:AAAG**	5:138404361	Multi-trait	A	0.326	1.08 (1.03–1.13)	2.62×10^–8^	Intronic	*CTNNA1*; *SIL1*
**rs112658458**	11:49790181	Multi-trait	A	0.967	1.19 (1.14–1.32)	8.31×10^–9^	Intronic	*OR4C12*; *OR4C13*
**rs78598167**	18:6110865	Multi-trait	T	0.023	1.33 (1.31–1.56)	8.36×10^–15^	Intronic	*L3MBTL4*
**rs5924943**	X:150594104	Multi-trait	T	0.469	1.07 (1.07–1.12)	3.76×10^–16^	Intergenic	*VMA21*
**rs141733360**	4:14727977	Chronic dry cough	A	0.009	0.51 (0.41–0.64)	4.20×10^–9^	ncRNA intronic	*CPEB2*
**rs11172406**	12:58366606	ACEi-induced cough	A	0.361	1.11 (1.07–1.15)	1.73×10^–8^	Intergenic	*ATP23*; *CYP27B1*
**rs35336617**	12:85913015	ACEi-induced cough	T	0.703	0.87 (0.83–0.91)	2.71×10^–9^	Intergenic	*ALX1*; *RASSF9*

The single-trait analyses identified three novel signals mapping to five novel genes (table 1). While the sentinel variant found for chronic dry cough (rs141733360), and its mapped gene (*CPEB2*) have not been described previously in the literature as associated with any cough phenotype, the only phenotypes we observed associated with rs141733360 in the PheWAS were cough-related (supplementary table S14). The two sentinel variants for ACEi-induced cough mapped to *ATP23* and *CYP27B1* (rs11172406), and *ALX1* and *RASSF9* (rs35336617), none of which have been identified before for any cough phenotype. Our PheWAS showed that the allele associated with increased ACEi-induced cough (allele A of rs11172406), mapping to *ATP23* and *CYP27B1*, was associated with reduced urea (β±se −0.010±0.002; FDR=6.21×10^–3^) and urate levels (β±se −0.017±0.002; FDR=7.85×10^–14^). This finding was supported by the Open Targets query for its proxy rs10877067 (in high linkage disequilibrium with rs11172406, r^2^=0.88); allele T at this variant, which increased risk of ACEi-induced cough, was also associated with reduced urate levels (β (95% CI) −0.0224 (0.018–0.026); p=2.60×10^–29^) (supplementary tables S14 and S15).

In addition, we identified seven previously reported signals for ACEi-induced cough, six in the multi-trait analysis and one in the ACEi-induced cough single-trait analysis. Collectively, these mapped to nine genes (*KCNA10*, *KCNIP4*, *L3MBTL4*, *MAPKAP1*, *NTSR1*, *PREP*, *RBM15*, *SLCO4A1* and *SRBD1*), three of which (*KCNA10*, *MAPKAP1* and *RBM15*) were never before implicated for any cough phenotype (supplementary table S6). The PheWAS showed that the signal mapping to *MAPKAP1* was associated with increased risk of asthma [13] (OR 1.016, 95% CI 1.007–1.025; FDR=9.62×10^–3^) (supplementary table S16), while the signal mapping to *NTSR1* and *SLCO4A1* was associated with both risk of asthma [13] (OR 1.026, 95% CI 1.014–1.039; FDR=4.43×10^–4^) (supplementary table S16) and cough-related phenotypes (FDR <1%) (supplementary table S14) with the same direction of effect.

The 13 novel genes encode proteins with diverse functions, albeit with some commonalities (figure 3; supplementary table S12), including cellular structure and transport (*CTNNA1*, *RASSF9*); cell growth, division and repair (*ATP23*, *CPEB2*, *MAKPAK1*); gene expression (*ALX1*, *RBM15*); homeostasis (*VMA21* and *CYP27B1*); neuronal signalling (*CTNNA1*, *KCNA10*, *MAPKAP1*, *OR4C12*, *OR4C13*, *SIL1*); and protein processing (*SIL1*). Specifically, CTNNA1 is a member of the catenin protein family which is involved in cell–cell adhesion [30] and synapse morphogenesis and plasticity [39], and RASSF9 has a role in intracellular and endosomal transport, protein targeting and signal transduction [31]. ATP23 is an enzyme involved in DNA repair; CPEB2 is important for cell cycle regulation; ALX1 is a transcription factor; and RBM15 also regulates transcription. CYP27B1 metabolises vitamin D which is involved in calcium homeostasis and VMA21 is involved in the maintenance of intracellular pH. MAPKAP1 is a subunit of the mammalian target of rapamycin (mTOR) complex 2, a component of the mTOR signalling pathway vital for growth and metabolism [30, 31, 40]. *MAPKAP1* has been identified in GWAS of pain intensity [41], and dysregulation of the mTOR signalling pathway has been implicated in various neurodegenerative and neuropsychiatric disorders, including epilepsy [42]. OR4C12 and OR4C13 are G-protein coupled receptors predominantly expressed in nasal epithelium olfactory sensory neurons and involved in neural response to odorant stimuli [30, 31]. SIL1 is a glycoprotein involved in protein translocation and folding and is associated with Marinesco–Sjögren syndrome [32], which is characterised by a number of neurological symptoms such as cerebellar ataxia and intellectual disability [43].

We identified the gene product of *KCNA10*, a subunit of potassium ion channels that have a role in neuronal excitability, neurotransmitter release and smooth muscle contraction [30, 31], as a target for several potassium channel blockers, including fampridine, which has been approved for treatment of multiple sclerosis, as well as others currently undergoing clinical trials for other indications including stroke and spinal cord injury. Reminertant, a drug targeting the protein encoded by *NTSR1*, is also undergoing trials for small cell lung carcinoma (supplementary table S13).

Five of the seven sentinels previously reported for ACEi-induced cough [16] showed association (p<0.05) with chronic dry cough, more than expected by chance (binomial test p=6×10^−6^) (supplementary table S7). These mapped to the *SRBD1*, *KCNIP4*, *PREP*, *SCAI* and *NTSR1/SLCO4A1* genes [16], all five variants showing a consistent direction of effect between chronic dry cough and ACEi-induced cough and a larger effect size estimate for chronic dry cough than for cough on most days [16]. The association with chronic dry cough met a Bonferroni threshold for the sentinels at the *SRBD1* (rs1544730), *PREP* (rs7761208) and *NTSR1/ SLCO4A1* (rs6062847) loci.

Our sensitivity analyses for chronic dry cough, excluding short-term chronic dry cough cases (supplementary table S17; supplementary figure S3), and for ACEi-induced cough, excluding cases with a cough diagnosis within 12 months of switching to an ARB (supplementary table S18; supplementary figure S4), did not show any systematic or substantial attenuation of effect sizes of our sentinel variants. Additionally, although two sentinel variants were associated with increased risk of asthma, our other sensitivity analysis of both chronic dry cough and ACEi-induced cough showed that exclusion of asthma cases also did not substantially affect sentinel effect sizes (supplementary table S19; supplementary figure S5). Furthermore, sex-stratified and sex-interaction analyses showed that our sentinels were not driven by sex-specific effects (supplementary table S20). We did not observe any significant SNP heritability enrichment of gene expression or specific chromatin structures across any of the tissue types analysed (supplementary figure S6).

We identified 2760 cases of ACEi-induced cough in All of Us (supplementary table S3), allowing us to corroborate the collective findings from our EUR ancestry discovery cohorts in the form of ACEi-induced cough PGS association with ACEi-induced cough in All of Us participants of EUR ancestries (OR 1.27, 95% CI 1.21–1.33 per sd unit increase in the PGS; p=7.45×10^−23^). We then evaluated the transferability of findings across different ancestry groups; the EUR ancestry-derived PGS for ACEi-induced cough was associated with ACEi-induced cough in participants of AFR ancestries from UK Biobank and All of Us (OR 1.09, 95% CI 1.00–1.19 per sd unit increase in the PGS; p=0.045) and participants of SAS ancestries in Genes & Health (OR 1.23, 95% CI 1.14–1.32 per sd unit increase in the PGS; p=4.31×10^−8^) (supplementary table S5).

**FIGURE 4:**
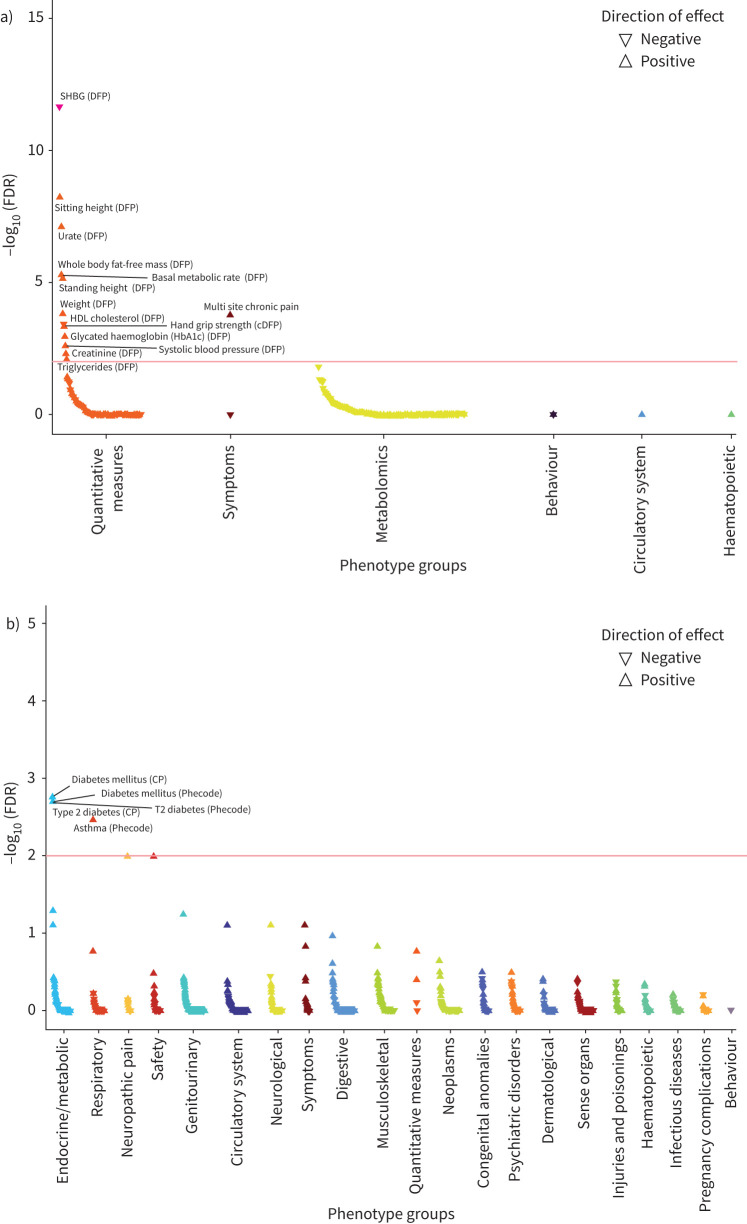
Angiotensin-converting enzyme induced cough polygenic score-based phenotype-wide association study results stratified by a) quantitative and b) binary phenotypes. FDR: false discovery rate; DFP: data-field phenotype; cDFP: combined data-field phenotype; CP: composite phenotype.

To inform understanding of the broader clinical consequences of cough-associated variants, we tested association between the ACEi-induced cough PGS and 1939 UK Biobank phenotypes. The ACEi-induced cough PGS was associated with quantitative phenotypes including reduced sex hormone binding globulin (β±se=−0.013±0.002; FDR=2.13×10^–12^) and reduced high-density lipoprotein cholesterol (β±se=−0.008±0.002; FDR=3.71×10^–4^), increased fat-free mass (β±se=0.006±0.001; FDR=5.13×10^–6^) and increased metabolic rate (β±se=0.006±0.001; FDR=5.13×10^–6^) (figure 4a; supplementary table S21). Most notably, an increase in the PGS was associated with increased multi-site chronic pain (β±se=0.007±0.001; FDR=1.68×10^–4^), which has been reported as a comorbidity accompanying chronic cough [44]. Furthermore, an increase in the PGS was associated with disease risk, including increased risk of diabetes (OR 1.03, 95% CI 1.02–1.05; FDR=1.72×10^–3^) and asthma (OR 1.03, 95% CI 1.01–1.04; FDR=3.09×10^–3^) (figure 4b; supplementary table S21). Supporting this, ACEi-induced cough was significantly genetically correlated with multi-site chronic pain (*r_g_*=0.20, p=9.00×10^–4^), asthma (*r_g_*=0.19, p=2.30×10^–3^) and type 2 diabetes (*r_g_*=0.12, p=1.75×10^–2^). Similarly, chronic dry cough also showed significant genetic correlations with both multi-site chronic pain (*r_g_=*0.31*,* p=6.04×10^–9^) and asthma (*r_g_=*0.30, p=3.40×10^–8^), with these correlations being stronger than those for ACEi-induced cough (supplementary figure S7). These findings support further that ACEi-induced cough and chronic dry cough share the same underlying phenotype.

## Discussion

Chronic cough is a common symptom, with prevalence estimates varying from 1–2% to 10% [3, 45], and imposes a substantial health burden [1]. Despite this, its biological mechanisms are not fully understood. We have utilised GWAS approaches to identify associated genetic variants and implicate relevant genes, shedding light on the molecular basis of chronic dry cough and ACEi-induced cough, and providing genetic evidence for future therapeutic developments. Specifically, we undertook the first GWAS of chronic dry cough and the largest multi-ancestry GWAS of ACEi-induced cough to date, and having demonstrated genetic overlap between chronic dry cough and ACEi-induced cough, we performed a multi-trait GWAS, thereby maximising power to discover novel variants associated with both traits. Further, we showed that the polygenic risk of ACEi-induced cough was associated with raised risk of conditions including asthma, diabetes and multi-site chronic pain, providing further insights as to the potential consequences of modulation of pathways involved in chronic cough.

We found 14 sentinel variants associated with these cough phenotypes which reached p<5×10^–8^ in the multi-trait or single-trait analyses. We demonstrated that these variants are robust to the presence of asthma, and inclusion of short-term chronic dry cough cases and ACEi-induced cough cases where cough may not have resolved after ACEi cessation, by showing consistent variant effects in sensitivity analyses excluding these conditions. Among our 14 sentinel variants, seven were novel, and five previously reported for ACEi-induced cough showed relevance for chronic dry cough. Through our comprehensive variant-to-gene mapping strategy, we mapped the seven novel sentinels to 10 genes not previously implicated in cough phenotypes (*SIL1*, *CTNNA1*, *OR4C12*, *OR4C13*, *VMA21*, *CPEB2*, *ATP23*, *CYP27B1*, *ALX1*, *RASSF9*), and we additionally mapped known loci to a further three novel genes (*MAPKAP1*, *KCNA10*, *RMB15*). As the sample sizes of studies with genome sequencing data grow, studies of rare variant associations with these phenotypes will help to assign the putative causal genes with greater confidence.

Collectively, the novel sentinels and genes, alongside those previously reported, enrich our understanding of the biological mechanisms underlying chronic dry cough and ACEi-induced cough. Many of the novel genes encode proteins with neurological functions (*CTNNA1*, *KCNA10*, *MAPKAP1*, *OR4C12*, *OR4C13*, *SIL1*), notably including KCNA10, a target of an approved drug for managing multiple sclerosis symptoms. Additionally, previously reported genes for ACEi-induced cough such as *KCNIP4*, *NTSR1* and *PREP* further strengthen the evidence supporting the neurobiological basis underlying the pathophysiology of chronic cough. Furthermore, we provide evidence that loci, genes and pathways previously implicated in ACEi-induced cough have a role in chronic dry cough and may provide insights into potential therapeutic targets [16, 19, 46]. Like *KCNA10*, *KCNIP4* encodes a potassium ion channel subunit and modulates neuronal excitability [30], and animal studies have provided evidence for the role of neuronal potassium channels in smooth muscle reactivity and cough reflex modulation [47]. NTSR1 mediates the activity of neurotensin, a pro-inflammatory peptide involved in the modulation of smooth muscle contraction [48]; is a target for a drug undergoing trials for small cell lung carcinoma; and also upregulates the secretion of γ-aminobutyric acid (GABA) [31]. Animal models and controlled trials have demonstrated that centrally acting agonists of GABA_B_ receptors inhibit the cough reflex [49, 50]; with baclofen being reported to suppress ACEi-induced cough [51]. PREP is involved in the mediation of neuropeptide activity, including the metabolism of bradykinin [52, 53], which has been shown to sensitise airway sensory nerves and induce cough reflex hypersensitivity in an animal model *via* the activation of airway C-fibres [54].

We found that the PGS for ACEi-induced cough was associated with increased multi-site chronic pain, a known comorbidity of chronic cough. The neurobiological mechanisms of chronic cough and chronic pain exhibit similarities, with central and peripheral sensitisation contributing to their underlying symptoms, and afferent nerve fibres involved in both conditions expressing common receptors [55]. P2X3 receptors are involved in nerve fibre sensitisation in chronic pain [56], and P2X3 receptor antagonists have shown antitussive efficacy in refractory and unexplained chronic cough patients in clinical trials [57].

By leveraging questionnaire responses from UK Biobank to define chronic dry cough and primary care electronic health record prescribing data to characterise ACEi-induced cough, we conducted the most powerful GWAS of chronic dry cough to date. This phenotyping approach allowed us to maximise the use of relevant phenotypic data available and overcome the limitation that drug response and adverse reaction-related traits are rarely specifically recorded in study populations, facilitating the study of ACEi-induced cough in large sample sizes across multiple cohorts. Another strength of this study is the implementation of a detailed variant-to-gene mapping framework to implicate putative causal genes and highlight biological mechanisms. Furthermore, we utilised recently developed techniques to improve power for understanding the potential consequences of pathway modulation through PGS-informed PheWAS. The SNP heritability estimates for chronic dry cough and ACEi-induced cough were comparable to that of other respiratory traits, such as asthma and COPD, where GWAS have been used to inform mechanistic understanding and identify drug targets.

We note potential limitations of our study. Misclassification of cases and controls is possible, particularly in the ACEi-induced cough GWAS when using an ACEi-to-ARB switch to characterise cases. However, such misclassification would tend to reduce effect size estimates towards the null and miss true associations. Furthermore, using an ACEi-to-ARB switch to capture ACEi-induced cough has been validated through manual interrogation of patient histories [18, 58] and consistency of genetic associations [16, 19], and we also show consistent PGS associations in independent populations. Confounding due to measured or unmeasured environmental, lifestyle or socioeconomic factors may affect observation studies, but is unlikely to affect genetic associations as genetic variants are assigned randomly during gamete formation [59]. Although we included individuals of diverse ancestries, the sample sizes for non-EUR groups remained modest despite our efforts to maximise data availability by utilising both electronic health records and questionnaire responses. Underrepresentation of non-EUR ancestry groups is common in GWASs [60], requiring initiatives to boost underrepresented populations and improve generalisability of findings across ancestries [61]. Despite limited sample sizes, we show PGS associations indicative of similar mechanisms across ancestries.

A key aim of this study was to provide molecular insights into the causes of chronic dry cough and ACEi-induced cough; we did not set out to produce risk prediction tools for chronic cough, which would require further large studies. We note that variable terms are used to describe and categorise chronic cough, including cough hypersensitivity syndrome, refractory chronic cough and unexplained chronic cough [62]. We did not attempt to distinguish these conditions, which would generally require specialist clinical assessment, given that cough is poorly coded in electronic health records [5] and not widely characterised in large cohort questionnaires. Such studies will require further development of registries of well-characterised patients with samples and consent for genetic studies, such as NEuroCOUGH [8].

Our findings expand on the established concept of cough hypersensitivity due to neuronal dysfunction, which in specific cases can be identified as a treatable trait, by showing that this mechanism applies broadly to chronic dry cough at the population level, and to ACEi-induced cough. Furthermore, identifying the association between genetic predisposition to cough with various known comorbidities, including chronic pain, could further advance our understanding of involved biological processes and should be considered in drug discovery and development efforts.

## Shareable PDF

10.1183/13993003.02341-2024.Shareable1 This PDF extract can be shared freely online.
Shareable PDF

ERJ-02341-2024.Shareable

## Data Availability

Access to UK Biobank (https://www.ukbiobank.ac.uk), the EXCEED Study (https://exceed.org.uk/), Genes & Health (https://www.genesandhealth.org/) and Copenhagen Hospital Biobank individual-level data are available to approved researchers upon application or data access request. Genome-wide summary statistics from the single-trait and multi-trait analyses are available *via* the EMBL-EBI GWAS Catalog under accession numbers GCST90573175, GCST90573176 and GCST90573177. The ACEi-induced cough phenotyping algorithm is publicly available (https://doi.org/10.5281/zenodo.6780065), and scripts used to run additional analyses are available upon request.

## Supplementary material

**Please note:** supplementary material is not edited by the Editorial Office, and is uploaded as it has been supplied by the author. Supplementary material ERJ-02341-2024.Supplement
