# Supplementary material for: Genomics of chronic dry cough unravels neurological pathways
Source: Eur Respir J. 2025 Sep 25;66(3):2402341. doi: 10.1183/13993003.02341-2024 (PMC12461901; doi:10.1183/13993003.02341-2024)
Supplement: Supplementary file 1 [file ERJ-02341-2024.Shareable.pdf]

EUROPEAN RESPIRATORY JOURNAL
ORIGINAL RESEARCH ARTICLE
K. COLEY ET AL.

SHAREABLE PDF

Genomics of chronic dry cough unravels neurological pathways

Kayesha Coley, Catherine John, Jonas Ghouse, David J. Shepherd, Nick Shrine, Abril G. Izquierdo, Stavroula Kanoni, Emma F. Magavern, Richard Packer, Lorcan McGarvey, Jaclyn A. Smith, Henning Bundgaard, Sisse R. Ostrowski, Christian Erikstrup, Ole B.V. Pedersen, David A. van Heel, Genes and Health Research Team, William Hennah, Mikko Marttila, Robert C. Free, Edward J. Hollox, Louise V. Wain, Martin D. Tobin and Chiara Batini

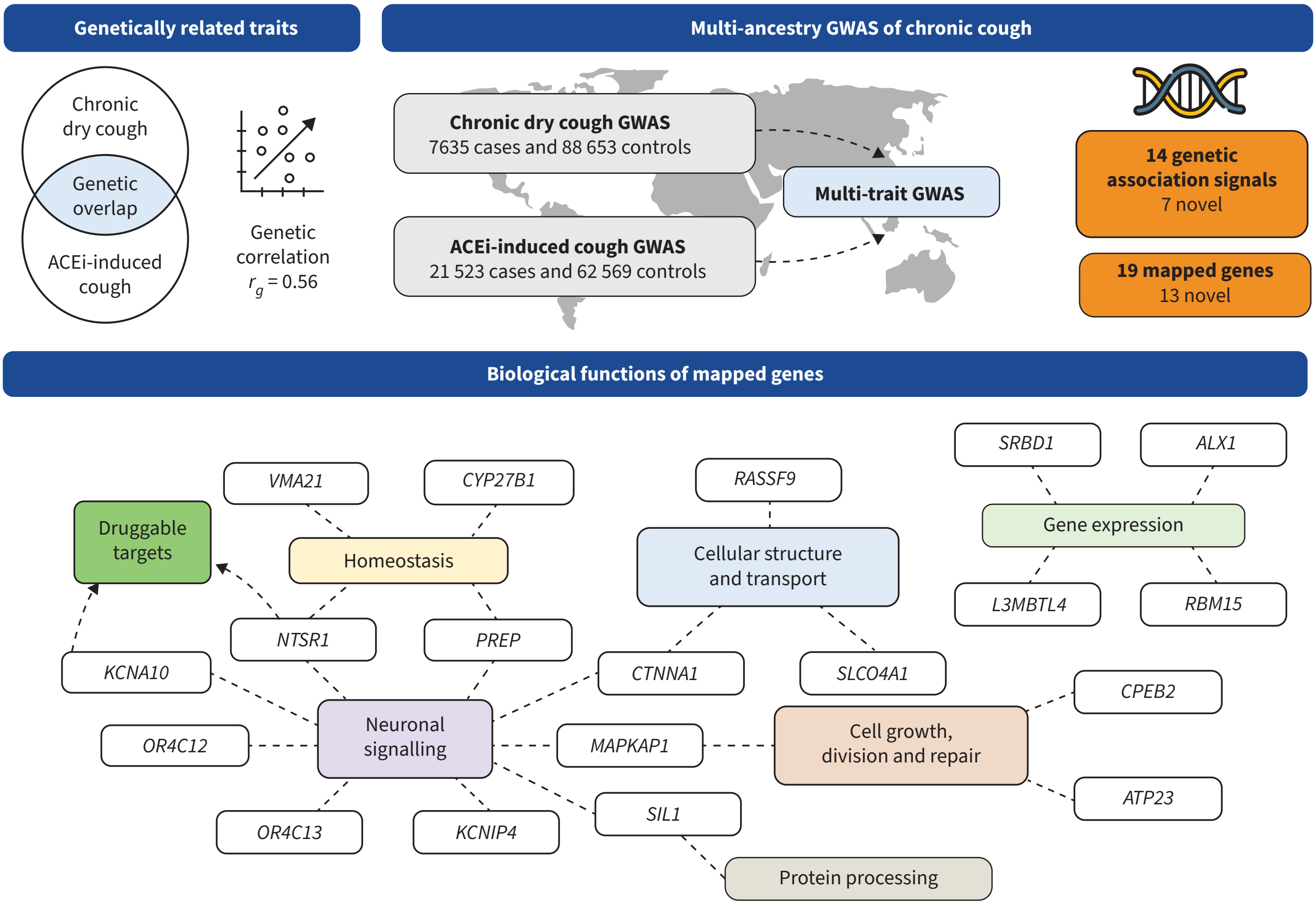

GRAPHICAL ABSTRACT Overview of the study. GWAS: genome-wide association study; ACEi: angiotensin-converting enzyme inhibitor.

SHAREABLE PDF

Genomics of chronic dry cough unravels neurological pathways

Kayesha Coley [1], Catherine John [1,2], Jonas Ghouse [3,4], David J. Shepherd[1], Nick Shrine [1], Abril G. Izquierdo[1], Stavroula Kanoni [5], Emma F. Magavern [5], Richard Packer [1,2], Lorcan McGarvey [6], Jaclyn A. Smith [7], Henning Bundgaard [8,9], Sisse R. Ostrowski [9,10], Christian Erikstrup[11,12], Ole B.V. Pedersen[9,13], David A. van Heel [14], Genes and Health Research Team[5], William Hennah [15,16], Mikko Marttila[17], Robert C. Free [2,18], Edward J. Hollox [19], Louise V. Wain [1,2], Martin D. Tobin [1,2] and Chiara Batini [1,2]

[1]Department of Population Health Sciences, University of Leicester, Leicester, UK. [2]University Hospitals of Leicester NHS Trust, Leicester, UK. [3]Laboratory for Molecular Cardiology, Department of Cardiology, Copenhagen University Hospital, Rigshospitalet, Copenhagen, Denmark. [4]Laboratory for Molecular Cardiology, Department of Biomedical Sciences, University of Copenhagen, Copenhagen, Denmark. [5]William Harvey Research Institute, Barts and the London School of Medicine and Dentistry, Queen Mary University of London, London, UK. [6]Wellcome-Wolfson Institute for Experimental Medicine, School of Medicine, Dentistry and Biomedical Sciences, Queen's University Belfast, Belfast, UK. [7]Division of Immunology, Immunity to Infection and Respiratory Medicine, The University of Manchester, Manchester University NHS Foundation Trust, Manchester, UK. [8]Department of Cardiology, Copenhagen University Hospital, Rigshospitalet, University of Copenhagen, Copenhagen, Denmark. [9]Department of Clinical Medicine, University of Copenhagen, Copenhagen, Denmark. [10]Department of Clinical Immunology, Rigshospitalet, Copenhagen University Hospital, Copenhagen, Denmark. [11]Department of Clinical Immunology, Aarhus University Hospital, Aarhus, Denmark. [12]Department of Clinical Medicine, Aarhus University, Aarhus, Denmark. [13]Department of Clinical Immunology, Zealand University Hospital, Køge, Denmark. [14]Blizard Institute, Barts and the London School of Medicine and Dentistry, Queen Mary University of London, London, UK. [15]Orion Pharma, Espoo, Finland. [16]Neuroscience Center, HiLIFE, University of Helsinki, Helsinki, Finland. [17]Orion Pharma, Nottingham, UK. [18]School of Computing and Mathematical Sciences, University of Leicester, Leicester, UK. [19]Department of Genetics and Genome Biology, University of Leicester, Leicester, UK.

Corresponding author: Kayesha Coley (kayesha.coley@leicester.ac.uk)

Check for updates

Shareable abstract (@ERSpublications)
This study leverages the genetics of two strongly correlated chronic cough traits (chronic dry cough and ACE inhibitor-induced cough). It highlights the contribution of neurobiological processes and provides new evidence for drug development and repurposing. https://bit.ly/3H0MHxy

Cite this article as: Coley K, John C, Ghouse J, *et al.* Genomics of chronic dry cough unravels neurological pathways. *Eur Respir J* 2025; 66: 2402341 [DOI: 10.1183/13993003.02341-2024].

This PDF extract can be shared freely online.

Copyright ©The authors 2025.

This version is distributed under the terms of the Creative Commons Attribution Licence 4.0.

This article has an editorial commentary: https://doi.org/10.1183/13993003.01267-2025

Received: 2 Dec 2024
Accepted: 14 May 2025

Abstract

Background Chronic dry cough is a symptom of common lung conditions, can occur as a side-effect of angiotensin-converting enzyme inhibitors (ACEis), or may be unexplained. Despite the substantial health burden presented by chronic dry cough, its biological mechanisms remain unclear. We hypothesised shared genetic architecture between chronic dry cough and ACEi-induced cough and aimed to identify causal genes underlying both phenotypes.

Methods We performed multi-ancestry genome-wide association studies (GWAS) of chronic dry cough and ACEi-induced cough, and a multi-trait GWAS of both phenotypes, utilising data from five cohort studies. Chronic dry cough was defined by questionnaire responses, and ACEi-induced cough by treatment switches or clinical diagnosis in electronic health records. We mapped putative causal genes and performed phenome-wide association studies (PheWAS) of associated variants, and polygenic scores for ACEi-induced cough, to identify pleiotropic effects.

Results We found seven novel genetic association signals reaching $p<5\times10^{-8}$ in the multi-trait or single-trait analyses of chronic dry cough and ACEi-induced cough. The novel variants mapped to 10 novel genes, and we mapped an additional three novel genes to known risk variants, many of which implicate neurological functions (*CTNNA1*, *KCNA10*, *MAPKAP1*, *OR4C12*, *OR4C13*, *SIL1*). The polygenic-score-based PheWAS highlighted associations with an elevated risk of several clinical conditions including asthma, diabetes and multi-site chronic pain.

Conclusion Our findings provide support for neuronal dysfunction underlying cough hypersensitivity in chronic dry cough and ACEi-induced cough, and identify diseases and traits associated with genetic predisposition to cough that could inform drug target discovery.
