# Supplementary material for: Genomics of chronic dry cough unravels neurological pathways
Source: Eur Respir J. 2025 Sep 25;66(3):2402341. doi: 10.1183/13993003.02341-2024 (PMC12461901; doi:10.1183/13993003.02341-2024)
Supplement: Supplementary file 2 [file ERJ-02341-2024.Supplement.pdf]

Supplementary Material

Genomics of chronic dry cough unravels neurological pathways

Kayesha Coley[1*], Catherine John[1,2], Jonas Ghouse[3,4], David J. Shepherd[1], Nick Shrine[1], Abril G. Izquierdo[1], Stavroula Kanoni[5], Emma F. Magavern[5], Richard Packer[1,2], Lorcan McGarvey[6], Jaclyn A. Smith[7], Henning Bundgaard[8,9], Sisse R. Ostrowski[9,10], Christian Erikstrup[11,12], Ole B. V. Pedersen[9,13], David A. van Heel[14], Genes & Health Research Team[5], William Hennah[15,16], Mikko Marttila[17], Robert C. Free[2,18], Edward J. Hollox[19], Louise V. Wain[1,2], Martin D. Tobin[1,2], Chiara Batini[1,2*]

[1]Department of Population Health Sciences, University of Leicester, Leicester, UK
[2]University Hospitals of Leicester NHS Trust, Groby Road, Leicester LE3 9QP, UK
[3]Laboratory for Molecular Cardiology, Department of Cardiology, Copenhagen University Hospital, Rigshospitalet, Building 9312, Henrik Harpestrengs Vej 4C, 2100 Copenhagen, Denmark
[4]Laboratory for Molecular Cardiology, Department of Biomedical Sciences, University of Copenhagen, Copenhagen, Denmark
[5]William Harvey Research Institute, Barts and the London School of Medicine and Dentistry, Queen Mary University of London, London, UK
[6]Wellcome-Wolfson Institute for Experimental Medicine, School of Medicine, Dentistry and Biomedical Sciences, Queen's University Belfast, Belfast, UK
[7]Division of Immunology, Immunity to Infection and Respiratory Medicine, The University of Manchester, Manchester University NHS Foundation Trust, Manchester, UK
[8]Department of Cardiology, Copenhagen University Hospital, Rigshospitalet, University of Copenhagen, Copenhagen, Denmark
[9]Department of Clinical Medicine, University of Copenhagen, Copenhagen, Denmark
[10]Department of Clinical Immunology, Rigshospitalet, Copenhagen University Hospital, Copenhagen, Denmark
[11]Department of Clinical Immunology, Aarhus University Hospital, Aarhus, Denmark
[12]Department of Clinical Medicine, Aarhus University, Aarhus, Denmark
[13]Department of Clinical Immunology, Zealand University Hospital, Køge, Denmark
[14]Blizard Institute, Barts and the London School of Medicine and Dentistry, Queen Mary University of London, London, UK
[15]Orion Pharma, Espoo, Finland
[16]Neuroscience Center, HiLIFE, University of Helsinki, Helsinki, Finland
[17]Orion Pharma, Nottingham, UK
[18]School of Computing and Mathematical Sciences, University of Leicester, Leicester, UK
[19]Department of Genetics and Genome Biology, University of Leicester, Leicester, UK

[*]Correspondence: kayesha.coley@leicester.ac.uk (KC) and cb334@leicester.ac.uk (CB)

Table of Contents

Supplementary Methods *4*

Study populations **4**

Phenotype definitions **4**

Discovery genome-wide association studies **5**

Sex-stratified and sex interaction testing **5**

Polygenic scores **5**

Investigating clinical and biological relevance of genetic associations **6**

Heritability and phenotypic variance explained **6**

Genetic correlations **7**

Sensitivity analyses **7**

Supplementary Tables *8*

Supplementary Table 1. GWAS protocols for participating discovery cohorts. **8**

Supplementary Table 2. Description of studies included in Respiratory PheWAS. **9**

Supplementary Table 3. Sample sizes and demographics. **10**

Supplementary Table 4. SNP-heritability estimates **11**

Supplementary Table 5. ACEi-induced cough polygenic score association testing across cohorts and ancestral groups. **12**

Supplementary Table 6. Independent sentinel variants identified in multi-trait GWAS of chronic dry cough and ACEi-induced cough, and additional sentinels from each single-trait GWAS. **13**

Supplementary Table 7. Association statistics for sentinels from multi-trait GWAS of chronic dry cough and ACEi-induced cough, in each single-trait GWAS. **14**

Supplementary Table 8. Summary of protein-coding genes identified by variant-to-gene mapping. **15**

Supplementary Table 9. Variants with posterior inclusion probability (PIP) ≥10% identified through fine-mapping of multi-trait loci and additional independent loci from each single-trait GWAS. **16**

Supplementary Table 10. Genes with highest polygenic priority score (PoPs) within specified window of sentinels identified in the multi-trait GWAS and additional sentinels variants from each single-trait GWAS. **17**

Supplementary Table 11. QTL colocalisation results. **18**

Supplementary Table 12. Functions of mapped genes. **19**

Supplementary Table 13. Druggability. **20**

Supplementary Table 14. Sentinel variant PheWAS results (false discovery rate [FDR] <0.01) using DeepPheWAS. **21**

Supplementary Table 15. Open Targets Genetics 'GWAS lead variants' results. **22**

Supplementary Table 16. Sentinel variant Respiratory PheWAS results (false discovery rate [FDR] <0.01). **23**

Supplementary Table 17. Sensitivity analysis excluding short-term chronic dry cough. **24**

Supplementary Table 18. Sensitivity analysis excluding ACEi-induced cough cases with a cough code within 12 months of switching to an ARB. **25**

Supplementary Table 19. UK Biobank sensitivity analysis excluding asthma. **26**

Supplementary Table 20. Sex-stratified association testing and sex-interaction testing of sentinel variants in UK Biobank (European only). **27**

Supplementary Table 21. Polygenic score PheWAS results (false discovery rate [FDR] <0.01) using DeepPheWAS. **29**

Supplementary Figures *30*

Supplementary Figure 1A. Forest plots for novel sentinel variants. **30**

Supplementary Figure 1B. Forest plots for previously reported sentinel variants. **32**

Supplementary Figure 2. Putative causal genes identified by variant-to-gene mapping. **34**

Supplementary Figure 3. Sensitivity analysis excluding cases who coughed for less than one-year from the chronic dry cough trait. **35**

Supplementary Figure 4. Sensitivity analysis in UK Biobank excluding cases with a clinical cough code within 12 months after switching to an ARB from the ACEi-induced cough trait. **36**

Supplementary Figure 5. Sensitivity analysis in UK Biobank excluding asthma cases from the chronic dry cough and ACEi-induced cough traits. **37**

Supplementary Figure 6. Enrichment of heritability of chronic dry cough and ACEi-induced cough across tissue types using expressed genes (A) and chromatin regions (B-F). **38**

Supplementary Figure 7. Genetic correlations of chronic dry cough and ACEi-induced cough with clinical conditions associated with genetic predisposition to ACEi-induced cough. **41**

References ***42***

Supplementary Methods

Study populations

UK Biobank[1] is a large-scale resource of approximately 500,000 individuals from across the UK, aged 40-69 during the recruitment period between 2006 and 2010. The genotyping and imputation protocol for UK Biobank samples is described extensively in Bycroft *et al* (2018). Genetically-determined ancestry was defined using *k*-means clustering of the first two genetic principal components (PCs)[2], which were provided by UK Biobank[1].

The EXCEED Study[3] is a cohort study with participants recruited from Leicester City, Leicestershire and Rutland. Driven mainly through general practices, recruitment started in 2013 and was aimed at individuals between 40 and 69 years. EXCEED samples were genotyped using the UK Biobank Axiom array, and samples were excluded prior to imputation where the call rate was <97% of if genetic sex did not match phenotypic sex. Variants with call rate <95%, Hardy-Weinberg *p*-value $<1\times10^{-6}$ or minor allele frequency (MAF) <1% were excluded. Genotype data were phased using Eagle v2.4 and imputation was performed using the TOPMed imputation server (imputation: Minimac4, reference panel: TOPMed r2). Genetic ancestry was defined using *k*-means clustering of the first two PCs (calculated using EIGENSOFT[4]) with 1000 Genomes Project superpopulations[5] as a reference.

Genes & Health[6] is a cohort of over 60,000 British Bangladeshi and British Pakistani individuals who have been recruited from across Bradford, East London, and Manchester since 2015. Further details are available in Finer *et al* (2020). Genotyping of Genes & Health samples was performed using Illumina GSAv3EAMD and imputed to the TOPMed reference panel.

Copenhagen Hospital Biobank[7] is a Danish biobank established to leverage residual blood samples collected during patient hospitalisations. Genotyping of samples in Copenhagen Hospital Biobank was performed using the Illumina Global Screening Array and underwent standard quality control protocol described in Sorensen *et al* (2021). Genotyped data were phased using Eagle v2 and imputed to reference panel backbone consisting of whole-genome sequence data from 8,429 Danes and 7,146 samples from North-Western Europe[8].

The Mosley *et al* study[9] utilised the electronic Medical Records and Genomics (eMERGE) Network[10], a collection of research institutions and medical centres across the United States which focusses on integrating biorepositories and electronic health records (EHRs). Genotyping was performed using various Illumina arrays (**Supplementary Table 1**), and quality control performed as described in Mosley *et al* (2016). Before imputing the genotype data to the 1000 Genomes phase 3[5] reference panel using IMPUTE2, phasing was performed using SHAPEIT[9]. Genetic ancestry was defined using STRUCTURE with HapMap as a reference panel[9].

The All of Us Research Program[11] is a United States-based longitudinal cohort study that aims to enrol at least one million individuals, with recruitment having started in 2018. The most recent release of the Controlled Tier Dataset (v8) includes 414,830 individuals with short-read whole genome sequencing data, the majority of which also have linked electronic health records. The processing and quality control of the whole genome sequencing data is described extensively in the 2024 publication from The All of Us Research Program Genomics Investigators[12]. Genetic ancestry for study participants was inferred using a random forest classifier trained on principal components calculated from the Human Genome Diversity Project (HGDP)[13] and 1000 Genomes[5] reference panels. Genetic ancestry fractions for each individual were calculated with the Rye program using 16 genetic principal components[12], and are provided on the Researcher Workbench.

Phenotype definitions

ACEi-induced cough was defined using primary care EHRs linked to UK Biobank[1], EXCEED[3] and Genes & Health[6] participants. Cases switched from an ACEi to an ARB within 12 months of the date of their first ACEi prescription (*index date*) and did not receive any ACEi prescriptions in 12 months after the switch date, and controls received at least one ACEi prescription and no ARB prescriptions within 12 months after *index date*. This definition was also applied to All of Us[11] using prescription data from EHRs. The definition in Copenhagen Hospital Biobank utilised dispensary data and was based on a switch to an ARB after continuous ACEi treatment[7,8]. In the published eMERGE Network study, ACEi-induced cough was defined using a validated algorithm based on prescriptions and the allergy section in EHRs; validation was conducted using manual record review across four study sites (25-50 cases each), finding positive predictive values (PPV) of case status of 100% at all sites (and PPV of control status of 96-100%)[9].

Chronic dry cough was defined in UK Biobank using questionnaire fields 22502 ('cough on most days') and 22504 ('bring up phlegm/sputum/mucus on most days'), as in the phenotype generation protocol implemented in DeepPheWAS[14]. Cases had a non-productive cough on most days (22502 = 1 & 22504 = 0) and controls did not have a productive cough on most days (22502 = 0 & 22504 = 0). We excluded individuals already defined as either ACEi-induced cough cases or controls, and any individuals related at second degree or closer to any ACEi-induced cough cases or controls. Questionnaire field 22503 ('years of cough on most days') was used for the sensitivity analysis which excluded chronic dry cases who reported having a cough on most days for less than one year.

Discovery genome-wide association studies

The discovery genome-wide association studies (GWASs) of chronic dry cough and ACEi-induced cough were performed under an additive genetic model using imputed genomic variants (imputation quality ≥ 0.3, and minor allele count [MAC] ≥10 in cases and controls) in genetically-defined ancestral groups containing ≥30 cases (**Supplementary Table 1**). We applied the recommended imputation quality filter of 0.3[15,16] to maximise overlap of variants during meta-analysis. We also utilised summary statistics with MAF >1% and imputation quality ≥ 0.7 from the previously published study conducted in the eMERGE Network[9] (**Supplementary Table 1**). All summary statistics were harmonised to genome build hg19 using *liftover*[17]. All ancestry-specific GWASs, single-trait GWASs, and the multi-trait GWAS had a genomic inflation factor (λ_{GC}) <1.05, hence no genomic-control correction was applied.

Using a fixed effects inverse variance-weighted meta-analysis in METAL[18], we meta-analysed chronic dry cough GWASs across UK Biobank European (EUR) and South Asian (SAS) groups. Similarly, we meta-analysed ACEi-induced cough GWASs undertaken in: UK Biobank (African [AFR], East Asian [EAS], EUR and SAS); EXCEED (EUR), Genes & Health (SAS), eMERGE Network (AFR, EUR, 'other', as defined by the Mosley *et al*)[9] and Copenhagen Hospital Biobank (EUR). These single-trait meta-analyses were included in the multi-trait GWAS of chronic dry cough and ACEi-induced cough, this was performed using a fixed effects inverse variance-weighted model (in METAL[18]) and included genetic variants represented in both datasets.

Conditionally independent signals were identified in each ancestral group separately using forward selection, followed by backward elimination in GCTA[19,20]. For each locus, unconditional summary statistics, and an ancestry matched reference panel (10,000 randomly selected EUR individuals in UK Biobank for EUR, or appropriate super-population from 1000 Genomes Phase 3[5] for AFR, EAS, and SAS) were used as input. Firstly, in each ancestral group, we conditioned on each sentinel using --cojo-cond, meta-analysed across all ancestries (to compile each single-trait, and again to create the multi-trait). If the sentinel was not present in an ancestral group, this group was removed from the meta-analysis. If there were any variants with p-value $<5\times10^{-8}$, we ran another round of conditional analysis including the original sentinel, and this sentinel, and repeated the process until there were no more sentinels reaching p-value $<5\times10^{-8}$. In backward selection, we obtained joint p-values for all sentinels in the conditional set for each locus using --cojo-joint, meta-analysed across ancestries (to compile each single-trait, and again to create the multi-trait), and if any variant did not reach p-value $<5\times10^{-8}$ we removed the variant with the largest p-value from the conditional set. If required, we iterated between forward selection and backward elimination until no additional variants outside the conditional set were genome-wide significant in the meta-analysis, and all variants in the conditional set had p-value $<5\times10^{-8}$ in the joint model post meta-analysis.

We calculated LD between the multi-trait sentinels and sentinels identified for each single trait, as well as all independent sentinels across the multi-trait and single-trait GWASs and previously reported variants, using the reference panel based on 10,000 randomly selected EUR individuals in UK Biobank. We used a threshold of r^2 <0.1 to define independence and novelty.

Using the approximate Bayes Factor method[21], we fine-mapped the 2Mb locus surrounding each sentinel variant calculate the posterior probability of inclusion (PIP) for each variant and generate 95% credible sets. We set the prior W to 0.04, and utilised unconditional summary statistics, unless a secondary signal was identified, then conditional summary statistics were used. We included variants with MAF >0.1% and p-value <0.001. After ranking variant-level PIPs from smallest to largest, the PIPs were summed until at least 95% was reached to create the 95% credible sets.

Sex-stratified and sex interaction testing

In UK Biobank (EUR-only), we conducted sex-stratified association analyses and sex interaction testing for our sentinel variants. For the sex-stratified analysis, we followed the primary analysis protocol (detailed above) and performed association testing with chronic dry cough and ACEi-induced cough in males and females separately. We then combined results for both traits within each sex using an inverse-variance weighted fixed effects model. For the sex interaction analysis, we modified the primary analysis protocol to implement the --interaction sex[0]option in REGENIE. We extracted the ADD-INT_SNPxsex interaction effects for each trait, and performed a multi-trait meta-analysis using an inverse-variance weighted fixed effects model.

Polygenic scores

Using PRS-CS[22], we constructed polygenic scores (PGS) of ACEi-induced cough based on overlapping variants between the 1000 Genomes (Phase 3) EUR reference panel and GWAS summary statistics from: the eMERGE Network and the EUR-only meta-analysis of UK Biobank, EXCEED and Copenhagen Hospital Biobank. We tested: (i) the association of the eMERGE Network PRS with ACEi-induced cough in UK Biobank (EUR), (ii) the EUR-only UK Biobank, EXCEED and Copenhagen Hospital Biobank PRS with ACEi-induced cough in All of Us (EUR), (iii) the EUR-only UK Biobank, EXCEED and Copenhagen Hospital Biobank PRS with ACEi-induced cough in All of Us (AFR), UK Biobank (AFR) and Genes & Health (SAS). Associations were tested using a logistic regression model, adjusted for age, age-squared, sex, genotyping array (UK Biobank testing populations only) the first 10 principal components of genetic ancestry. The within-ancestry results were

meta-analysed using a fixed effects inverse variance-weighted model (with the *metafor* R package) [**Supplementary Table 5**].

We also used PRS-CS[22] to construct a EUR-only PGS based on EXCEED and Copenhagen Hospital Biobank (1,109,552 variants) and tested association with 1,939 traits defined in UK Biobank (EUR) using DeepPheWAS[14].

Investigating clinical and biological relevance of genetic associations

(i) Nearest gene: We identified nearby genes to each sentinel using ANNOVAR[23] and selected the closest protein-coding gene.

(ii) Variant annotation and pathogenicity estimates: We used ANNOVAR and Variant Effect Predictor[24] to annotate each variant with PIP ≥10%, obtained through fine-mapping of associated loci (described above) with its consequence, SIFT and PolyPhen-2 predictors, and Combined Annotation Dependent Depletion (CADD) score. The nearest genes to variants annotated as deleterious by SIFT, probably damaging by PolyPhen-2 or with a deleterious CADD Phred score of ≥12.37 were selected (**Supplementary Table 7**).

(iii) Polygenic Priority Score (PoPS): PoPS[25] is a similarity-based method which leverages gene-level features derived from multiple sources, including gene expression datasets, biological pathways and protein-protein interactions, to perform gene prioritisation. We applied PoPS to the EUR-only summary statistics of the multi-trait GWAS and each single-trait GWAS to generate gene-level polygenic priority scores and used the EUR population from 1000 Genomes Project[5] Phase 3 as an LD reference. For this analysis, we selected the gene with the highest polygenic priority score (based on results using the relevant summary statistics) within ±250kb of each variant (extended to ±500kb when no gene was identified within ±250kb) [**Supplementary Table 8**].

(iv) Rare variant association: To support identifying nearby rare (MAC ≥3 and MAF <1%) variant associations, we undertook exome-wide association studies (ExWASs) of chronic dry cough and ACEi-induced cough using the UK Biobank exome sequences (OQFE pipeline) and REGENIE[26]. The REGENIE protocol was the same as reported in Supplementary Table. GWAS protocols for participating cohorts, and we excluded variants which did not pass the following PLINK filters[27]: --geno 0.1 --hwe 1e-15 --mind 0.1. Using METAL[18], for each trait we meta-analysed the ExWAS summary statistics from different ancestral groups, and subsequently meta-analysed both traits in a multi-trait ExWAS. Using the relevant ExWAS meta-analysis summary statistics, we selected genes harbouring a rare variant association (p-value $<5\times10^{-6}$) within ±250kb of each variant.

(v) Expression quantitative trait loci (eQTL) colocalisation: We performed a preliminary look-up of the lead variants in GWAS summary statistics of gene expression levels in two *cis*-eQTL resources: (i) eQTLGen[28], a blood-only resource, and (ii) GTeX v8[29], restricted to adrenal, artery, blood, brain, oesophagus, heart, ileum, liver, lung, muscle, nerve, pituitary, spleen and stomach tissues. We identified variant-gene-tissue combinations with p-value $< 5\times10^{-8}$, and tested the corresponding locus (in EUR only) for colocalisation with the eQTL signal using coloc.susie[30]. We used an LD reference based on imputed genotype data from 10,000 EUR individuals in UK Biobank. Genes with a $H_4 \geq 0.7$ were selected (**Supplementary Table 9**).

(vi) Protein quantitative trait loci (pQTL) colocalisation: We performed a preliminary look-up of the lead variants in GWAS summary statistics of protein levels across three pQTL resources: (i) deCODE Genetics (includes 4,719 proteins measured by 4,907 aptamers)[31], (ii) the SCALLOP Consortium (includes 90 cardiovascular proteins)[32]; and (iii) UK Biobank-Pharma Proteomics Project (PPP) [includes 2,923 proteins][33]. We identified variant-protein combinations with p-value $<$ 1.8×10^{-9} in the deCODE Genetics dataset (as used to determine significance by the authors in the original publication[31]) or p-value $<5\times10^{-8}$ in the SCALLOP Consortium and UK Biobank-PPP datasets. For associated variants identified as *cis*-pQTLs, i.e. located within ±1Mb of the transcription start site of the gene encoding the measured protein, we tested the corresponding locus (in EUR only) for colocalisation with the pQTL signal using coloc.susie[30]. We used an LD reference based on imputed genotype data from 10,000 EUR individuals in UK Biobank. Encoding genes with a $H_4 \geq 0.7$ were selected.

For the Respiratory PheWAS, we performed a look-up of the sentinel variants in 28 GWASs performed across 11 clinical respiratory traits: asthma, bronchiectasis, bronchopneumonia, chronic bronchitis, chronic obstructive pulmonary disease, chronic sputum production, emphysema, idiopathic pulmonary fibrosis, respiratory infections, interstitial lung abnormality, and pneumothorax. The sources of these datasets are provided in **Supplementary Table 2**.

Heritability and phenotypic variance explained

We estimated the SNP heritability of chronic dry cough and of ACEi-induced cough using LD Score Regression (LDSC)[34,35]. Our analysis was based on UK Biobank-European (for chronic dry cough and ACEi-induced cough, separately) and Copenhagen Hospital Biobank-European (for ACEi-induced cough only) GWAS summary statistics restricted to common HapMap3 variants, and 1000 Genomes (Phase 3) EUR derived LD Scores. To contextualise these estimates, also calculated SNP heritability of other respiratory traits; we retrieved EUR-only GWAS summary statistics from Global

Biobank Meta-analysis Initiative studies of asthma[36] and chronic obstructive pulmonary disease[37] and estimated SNP heritability using the same approach (**Supplementary Table 4**).

We used stratified LDSC (S-LDSC)[38] to estimate the heritability enrichment across specific tissue types by incorporating gene expression (GTeX[39]) and chromatic accessibility (Roadmap Epigenomics[40] and EN-TEx[41]) datasets. The analysis focussed on adrenal, artery, blood, brain, esophagus, heart, ileum, liver, lung, muscle, nerve, pituitary, spleen and stomach tissue types. Following the methodology in Finucane *et al* (2015), we utilised UK Biobank-EUR GWAS summary statistics for the two single traits (chronic dry cough and ACEi-induced cough) restricted to common HapMap3 variants, alongside 1000 Genomes (Phase 3)-derived LD scores from the 97-annotation baseline-LD v2.2 model[42]. For the gene expression and each chromatin modification (H3K4me3, H3K27ac, H3K9ac, H3K4me1, and H3K36me3) analysis, we used a Bonferroni-corrected significance threshold for the number of tissues to determine significant associations.

We estimated the proportion of phenotypic variance explained by all independently associated sentinel variants using the following approximation:

$$\sum_{i=1}^{n} \frac{2f_i(1-f_i)\beta_i^2}{2f_i(1-f_i)\beta_i^2 + (se(\beta_i))^2 2N_i f_i(1-f_i)}$$

Where n is the number of sentinel variants, and f_i is the effect allele frequency, β_i is the effect estimate and N_i is the sample size of the ith variant.

Genetic correlations

Using LDSC[34,35], we estimated the genetic correlation between chronic dry cough and ACEi-induced using on UK Biobank-European GWAS summary statistics for each trait (restricted to HapMap3 variants) and 1000 Genomes (Phase 3) EUR derived LD Scores. We also retrieved EUR-only summary statistics for clinical traits with an FDR <1% in the polygenic score-based PheWAS (described below; multi-site chronic pain[43], asthma[36] and type 2 diabetes[44]) and estimated their genetic correlation with each cough trait using the same methodology.

Sensitivity analyses

We performed sensitivity analyses under three scenarios:
First, for the chronic dry cough trait, we excluded cases who coughed for less than one year. For our sentinel variants, we followed the primary analysis protocol (outlined above) and performed ancestry-specific association testing with chronic dry cough, ensuring short-term cough cases were excluded. Using an inverse-variance weighted fixed effects model, we then performed a new meta-analysis for chronic dry cough, followed by a combined multi-trait meta-analysis of both traits (**Supplementary Table 17; Supplementary Figure 3**).
Second, for the ACEi-induced cough trait, we conducted a sensitivity analysis in UK Biobank only, excluding ACEi-induced cough cases with a clinical cough code recorded in primary care EHRs within 12 months after switching to the ARB. For our sentinel variants, we performed ancestry-specific association testing with ACEi-induced cough while excluding these cases. We then used an inverse-variance weighted fixed effects model to perform a new meta-analysis for ACEi-induced cough, followed by a combined multi-trait meta-analysis of both traits (**Supplementary Table 18; Supplementary Figure 4**).
Third, for both the chronic dry cough and ACEi-induced cough traits, we performed another sensitivity analysis in UK Biobank excluding all individuals with asthma (defined by DeepPheWAS[14] using both self-report and EHR data).For our sentinel variants, we performed ancestry-specific association testing with chronic dry cough and ACEi-induced cough, excluding asthma cases. Using an inverse-variance weighted fixed effects model, we conducted separate meta-analyses for each trait, followed by a combined multi-trait meta-analysis of both traits (**Supplementary Table 19; Supplementary Figure 5**).

Supplementary Tables

Supplementary Table 1. GWAS protocols for participating discovery cohorts.

Abbreviations: AFR, African; EAS, East Asian; EUR, European; HRC, Haplotype Reference Consortium; MAC, minor allele count; MAF, minor allele frequency; PC, principal component; SAS, South Asian; TOPMed, Trans-Omics for Precision Medicine.

	**UK Biobank**	**EXCEED**	**Genes & Health**	**eMERGE Network (Mosley *et al*, 2016)**	**Copenhagen Hospital Biobank**
**Genetic data**					
**Genotyping array**	Applied Biosystems UK BiLEVE Axiom; Applied Biosystems UK Biobank Axiom	Applied Biosystems UK Biobank Axiom	Illumina Infinium Global Screening Array	HumanOmni1-Quad; HumanOmni5-Quad; Human1M-Duov3_B; HumanOmniExpress-12v1.0; HumanOmniExpress; Human660W-Quadv1_A	Illumina Global Screening Array
**Imputation panel**	HRC and UK10K + 1000 Genomes Phase 3	TOPMed	TOPMed	1000 Genomes phase 3	Based on whole genome sequencing data from 8,429 Danes and 7,146 samples from North-West Europe
**Variant filters**	Imputation quality score ≥0.3; MAC_{cases} ≥10; $MAC_{controls}$ ≥10			Imputation quality score ≥0.7; MAF ≥1%.	Imputation quality score ≥0.3; MAC_{cases} ≥10; $MAC_{controls}$ ≥10
**# variants passing filters (chronic dry cough)**	EUR: 19,494,967<br>SAS: 6,402,724	–	–	–	–
**# variants passing filters (ACEi-induced cough)**	AFR: 6,963,210<br>EAS: 4,460,597<br>EUR: 17,101,867<br>SAS: 8,455,429	EUR: 6,808,289	SAS: 9,494,374	AFR, EUR, 'Other': 1,931,833	EUR: 10,559,321
**Association testing (additive genetic model)**					
**Software**	REGENIE			PLINK 1.07	BOLT-LMM
**Model**	Approximate Firth logistic regression			Logistic regression	Linear mixed model
**Covariates**	Age, age^2, sex, genotyping array, ever smoking status (chronic dry cough only), PCs 1-10	Age, age^2, sex, PCs 1-10	Age, age^2, sex, PCs 1-20	Birth year, sex, PCs 1-10	Age, age^2, sex, PCs 1-10

Supplementary Table 2. Description of studies included in Respiratory PheWAS.

Clinical phenotype	Clinical subtype	Cohort	Ancestry	*N*	Author (Year)	Reference	Additional information
Asthma	Asthma	GBMI	MULTI	1800785	Tsuo (2022)	[36]	
Asthma	Asthma	UK Biobank; deCODE	EUR	771388	Olafsdottir (2020)	[45]	
Asthma	Asthma adult onset	UK Biobank	EUR	327253	Ferreira (2019)	[46]	
Asthma	Asthma childhood onset	UK Biobank	EUR	314633	Ferreira (2019)	[46]	
Asthma	Asthma exacerbations in children	Danish	EUR	68281	Ahluwalia (2020)	[47]	
Asthma	Asthma exacerbations in adults	UK Biobank	EUR	49494	Edris (2024)	[48]	
Bronchiectasis	Bronchiectasis	UK Biobank; FinnGen;Biobank Japan	MULTI	605195	Sakaue (2021)	[49]	
Bronchopneumonia	Bronchopneumonia	UK Biobank	EUR	456348	Jiang (2021)	[50]	
Bronchopneumonia	Bronchopneumonia	UK Biobank	EUR	387930	Backman (2021)	[51]	
Chronic bronchitis	Chronic bronchitis	UK Biobank	EUR	378459	DeepPheWAS	[14]	Defined in UK Biobank using DeepPheWAS
Chronic bronchitis	Chronic bronchitis	FinnGen	EUR	410564	FinnGen	[52]	FinnGen DF12: J10_BRONCHNAS
Chronic obstructive pulmonary disease	Chronic obstructive pulmonary disease	UK Biobank	EUR	196578	DeepPheWAS	[14]	Defined in UK Biobank using DeepPheWAS
Chronic obstructive pulmonary disease	Chronic obstructive pulmonary disease	FinnGen	EUR	433208	FinnGen	[52]	FinnGen DF12: J10_COPD
Chronic obstructive pulmonary disease	Chronic obstructive pulmonary disease with acute exacerbation	UK Biobank	EUR	40047	Williams	NA	
Chronic sputum production	Chronic sputum production	UK Biobank	EUR	58185	Packer (2023)	[53]	
Emphysema	Emphysema	UK Biobank	EUR	178144	DeepPheWAS	[14]	Defined in UK Biobank using DeepPheWAS
Emphysema	Emphysema	FinnGen	EUR	411781	FinnGen	[52]	FinnGen DF12: J10_EMPHYSEMA
Idiopathic Pulmonary Fibrosis	Idiopathic pulmonary fibrosis	GBMI	MULTI	1375570	Partanen (2022)	[54]	
Idiopathic Pulmonary Fibrosis	Idiopathic pulmonary fibrosis	US;UK;Spain	EUR	24589	Allen (2022)	[55]	
Idiopathic Pulmonary Fibrosis	Idiopathic pulmonary fibrosis DCLO	US;UK;Spain	EUR	975	Allen (2023)	[56]	
Idiopathic Pulmonary Fibrosis	Idiopathic pulmonary fibrosis FVC	US;UK;Spain	EUR	1329	Allen (2023)	[56]	
Idiopathic Pulmonary Fibrosis	Idiopathic pulmonary fibrosis Survival	US;UK;Spain	EUR	1481	Oldham (2023)	[57]	
Respiratory infections	Respiratory infections primary care	UK Biobank	EUR	196552	Williams	NA	
Respiratory infections	Respiratory infections secondary care	UK Biobank	EUR	120897	Williams (2023)	[58]	
Interstitial lung abnormality	Interstitial lung abnormality	FHS;AGES;COPDGene;ECLIPSE;MESA;SPIROMICS	MULTI	11973	Hobbs (2019)	[59]	
Interstitial lung abnormality	Subpleural predominant interstitial lung abnormality	FHS;AGES;COPDGene;ECLIPSE;MESA;SPIROMICS	MULTI	11561	Hobbs (2019)	[59]	
Pneumothorax	Pneumothorax	FinnGen	EUR	482368	FinnGen	[52]	FinnGen DF12: J10_PNEUMOTHORAX
Pneumothorax	Pneumothorax	UKB;FinnGen;BiobankJapan	MULTI	654724	Sakaue (2021)	[49]	

Supplementary Table 3. Sample sizes and demographics.
Age for chronic dry cough is age at baseline recruitment, while age for ACEi-induced cough in UK Biobank, EXCEED and Genes & Health is age at *index date* (i.e.at first ACEi prescription). Abbreviations: AFR, African; EAS, East Asian; EUR, European; SAS, South Asian; SD, standard deviation.

Cohort	Ancestral group	Total sample size (% female)	Case count (% female)	Control count (% female)	Mean age (SD) in total sample, years	Mean age (SD) in cases, years	Mean age (SD) in controls, years
**Discovery**							
**Chronic dry cough**							
**UK Biobank**	EUR	95399 (57.5)	7575 (59.1)	87824 (57.3)	55.8 (7.7)	56.8 (7.5)	55.7 (7.7)
	SAS	889 (47.7)	60 (60.0)	829 (46.8)	52.6 (8.0)	54.1 (7.8)	52.4 (8.0)
**ACEi-induced cough**							
**UK Biobank**	AFR	408 (56.9)	48 (75.0)	360 (54.4)	53.0 (8.2)	54.5 (8.1)	52.8 (8.2)
	EAS	148 (52.0)	32 (46.9)	116 (53.4)	53.4 (7.7)	57.5 (7.9)	52.3 (7.2)
	EUR	37372 (42.4)	5049 (55.0)	32323 (40.5)	57.9 (7.7)	59.1 (7.2)	57.8 (7.7)
	SAS	1101 (36.7)	178 (43.8)	923 (35.3)	54.5 (8.1)	56.3 (8.0)	54.2 (8.0)
**EXCEED**	EUR	838 (42.8)	128 (51.6)	710 (41.3)	55.0 (7.2)	55.5 (5.9)	54.9 (7.5)
**Genes & Health**	SAS	7599 (45.0)	819 (57.8)	6780 (43.4)	48.5 (10.9)	48.5 (9.9)	48.5 (11.0)
**eMERGE Network (Mosley *et al*, 2016)**	Multiple ancestries (AFR, EUR, 'other')	7080 (48.4)	1595 (60.7)	5485 (44.8)	..	..	..
**Copenhagen Hospital Biobank**	EUR	29546	13674 (44.5)	15872 (39.0)	75.4 (12.2)	75.7 (11.9)	76.0 (12.4)
**Corroboration**							
**ACEi-induced cough**							
**All of Us**	AFR	7567 (57.1)	548 (67.5)	7019 (56.3)	52.1 (10.9)	53.7 (10.6)	52.0 (10.9)
	AMR	3771 (54.9)	281 (63.7)	3490 (54.2)	52.3 (12.2)	52.8 (12.7)	52.3 (12.2)
	EAS	310 (46.5)	57 (50.9)	253 (45.5)	51.5 (13.2)	52.6 (13.5)	51.2 (13.1)
	EUR	20020 (45.3)	1874 (56.8)	18146 (44.1)	58.0 (11.8)	59.4 (11.8)	57.8 (11.8)

Supplementary Table 4. SNP-heritability estimates.

Cohort	Trait	Ancestral group	SNP-heritability, h^2 (SE)	Proportion of variance explained by sentinel variants (%)
UK Biobank	Chronic dry cough	EUR	0.0318 (0.0052)	4.96
UK Biobank	ACEi induced cough	EUR	0.0534 (0.0138)	27.8
Copenhagen Hospital Biobank	ACEi-induced cough	EUR	0.0377 (0.0161)	–
Global Biobank Meta-analysis Initiative (GBMI)	Asthma	EUR	0.0260 (0.0019)	–
Global Biobank Meta-analysis Initiative (GBMI)	Chronic obstructive pulmonary disease	EUR	0.0183 (0.0011)	–

Supplementary Table 5. ACEi-induced cough polygenic score association testing across cohorts and ancestral groups.
Abbreviations: CI, confidence interval; OR, odds ratio.

Training				Number of variants	Testing					
Cohort	Ancestry	Cases	Controls		Cohort	Ancestry	Cases	Controls	OR [95% CI]	*p*-value
eMERGE Network	Multiple ancestries (AFR, EUR, 'other')	1595	5485	409070	UK Biobank	EUR	5049	32323	1.06 [1.02, 1.09]	3.87E-04
UK Biobank, EXCEED & Copenhagen Hospital Biobank	EUR	18851	48905	1116135	UK Biobank & All of Us	AFR	596	7520	1.09 [1.00, 1.19]	4.51E-02
UK Biobank, EXCEED & Copenhagen Hospital Biobank	EUR	18851	48905	1116135	All of Us	EUR	1880	18332	1.27 [1.21, 1.33]	7.45E-23
UK Biobank, EXCEED & Copenhagen Hospital Biobank	EUR	18851	48905	1094654	Genes & Health	SAS	819	6779	1.23 [1.14, 1.32]	4.31E-08

Supplementary Table 6. Independent sentinel variants identified in multi-trait GWAS of chronic dry cough and ACEi-induced cough, and additional sentinels from each single-trait GWAS.

Mapped genes in bold are novel. Direction column has the following order: UK Biobank-European, UK Biobank-South Asian for chronic dry cough; UK Biobank-African, UK Biobank-East Asian, UK Biobank-European, UK Biobank-South Asian, EXCEED-European, Genes & Health-South Asian, eMERGE Network, Copenhagen Hospital Biobank for ACEi-induced cough; chronic dry cough, ACEi-induced cough for multi-trait analysis. Mean imputation quality is weighted by case number in each contributing cohort. *Abbreviations:* CI, confidence interval; OR, odds ratio.

Sentinel ID	Position (hg19)	Effect allele	Other allele	Trait	Effect allele frequency	Mean imputation quality	OR [95% CI]	*p*-value	*N* cases	*N* controls	Direction	Annotation	Mapped gene(s)	Novel sentinel variant?	Previously reported sentinels within locus		
															ID (r^2)	Reported gene	Ref
rs1544730	2:45588067	A	G	Multi-trait	0.223	0.983	1.11 [1.10, 1.16]	1.72E-16	27515	145377	++	Intergenic	*SRBD1*	N	rs1544730 (1.00)	*SRBD1*	[8]
4:21393932:T:TAAG	4:21393932	T	TAAG	Multi-trait	0.419	0.983	1.13 [1.14, 1.20]	4.97E-32	27563	145737	++	Intronic	*KCNIP4*	N	rs16870989 (0.711)<br>rs145489027 (0.709)	*KCNIP4*<br>*KCNIP4*	[8]<br>[9]
5:138404361:A:AAAG	5:138404361	A	AAAG	Multi-trait	0.326	0.981	1.08 [1.03, 1.13]	2.62E-08	12894	122015	++	Intronic	***CTNNA1; SIL1***	Y	–	–	–
rs7761208	6:105776289	T	C	Multi-trait	0.788	0.996	0.89 [0.84, 0.89]	1.72E-20	27531	145621	--	Intronic	*PREP*	N	rs12210271 (0.982)	*PREP*	[8]
rs7848821	9:128251930	A	G	Multi-trait	0.320	0.990	1.08 [1.05, 1.11]	4.02E-12	29158	151222	++	Intronic	***MAPKAP1***	N	rs360206 (0.158)	*SCAI*	[8]
rs112658458	11:49790181	A	G	Multi-trait	0.967	0.976	1.19 [1.14, 1.32]	8.31E-09	27117	142799	++	Intronic	***OR4C12; OR4C13***	Y	–	–	–
rs78598167	18:6110865	T	C	Multi-trait	0.023	0.976	1.33 [1.31, 1.56]	8.36E-15	27295	143722	++	Intronic	*L3MBTL4*	Y	rs8097200 (0.003)	*L3MBTL4*	[8]
rs343240	18:6308062	T	C	Multi-trait	0.178	0.993	0.91 [0.83, 0.89]	4.90E-11	27563	145737	+-	Intronic	*L3MBTL4*	N	rs8097200 (0.986)	*L3MBTL4*	[8]
rs6062847	20:61322018	T	C	Multi-trait	0.137	0.997	1.18 [1.15, 1.24]	1.10E-31	29066	150277	++	Intergenic	*NTSR1; SLCO4A1*	N	rs6062847 (1.00)	*NTSR1; SLCO4A1*	[8]
rs5924943	X:150594104	T	C	Multi-trait	0.469	0.987	1.07 [1.07, 1.12]	3.76E-16	27531	145621	++	Intergenic	***VMA21***	Y	–	–	–
rs141733360	4:14727977	A	C	Chronic dry cough	0.009	0.836	0.51 [0.41, 0.64]	4.20E-09	6254	41212	-?	ncRNA intronic	***CPEB2***	Y	–	–	–
rs7518061	1:111087944	T	C	ACEi-induced cough	0.310	0.993	0.88 [0.84, 0.92]	1.85E-09	7849	46697	---+--??	Intergenic	***KCNA10; RBM15***	N	rs7526729 (0.995)	*KCNA2*	[8]
rs11172406	12:58366606	A	C	ACEi-induced cough	0.361	0.992	1.11 [1.07, 1.15]	1.73E-08	5259	33362	+-+++++?	Intergenic	***ATP23; CYP27B1***	Y	–	–	–
rs35336617	12:85913015	T	TC	ACEi-induced cough	0.703	0.996	0.87 [0.83, 0.91]	2.71E-09	7575	87824	?---????	Intergenic	***ALX1; RASSF9***	Y	–	–	–

Supplementary Table 7. Association statistics for sentinels from multi-trait GWAS of chronic dry cough and ACEi-induced cough, in each single-trait GWAS.
Direction column has the following order: UK Biobank-European, UK Biobank-South Asian for chronic dry cough; UK Biobank-African, UK Biobank-East Asian, UK Biobank-European, UK Biobank-South Asian, EXCEED-European, Genes & Health-South Asian, eMERGE Network, Copenhagen Hospital Biobank for ACEi-induced cough. Mapped genes in bold are novel. *Abbreviations*: CI, confidence interval; OR, odds ratio.

Sentinel ID	Position (hg19)	Effect allele	Other allele	Cough trait	Effect allele frequency	OR [95% CI]	*p*-value	Direction	Mapped gene(s)
rs1544730	2:45588067	A	G	Chronic dry cough	0.210	1.07 [1.02, 1.11]	2.23E-03	+-	*SRBD1*
				ACEi-induced cough	0.230	1.13 [1.10, 1.16]	1.60E-15	?+++++?+	
4:21393932:T:TAAG	4:21393932	T	TAAG	Chronic dry cough	0.410	1.06 [1.03, 1.10]	4.18E-04	++	*KCNIP4*
				ACEi-induced cough	0.424	1.17 [1.14, 1.20]	1.96E-33	+-++++?+	
5:138404361:A:AAAG	5:138404361	A	AAAG	Chronic dry cough	0.326	1.09 [1.05, 1.13]	5.99E-06	++	***CTNNA1; SIL1***
				ACEi-induced cough	0.326	1.08 [1.03, 1.13]	1.15E-03	?+++????	
rs7761208	6:105776289	T	C	Chronic dry cough	0.804	0.94 [0.90, 0.98]	5.19E-03	--	*PREP*
				ACEi-induced cough	0.779	0.86 [0.84, 0.89]	4.36E-21	+?--+-?-	
rs7848821	9:128251930	A	G	Chronic dry cough	0.310	1.07 [1.03, 1.11]	3.81E-04	++	***MAPKAP1***
				ACEi-induced cough	0.325	1.08 [1.05, 1.11]	2.10E-09	+-+-++++	
rs112658458	11:49790181	A	G	Chronic dry cough	0.965	1.13 [1.02, 1.24]	1.56E-02	+?	***OR4C12; OR4C13***
				ACEi-induced cough	0.968	1.23 [1.14, 1.32]	6.30E-08	??+??-?+	
rs78598167	18:6110865	T	C	Chronic dry cough	0.018	1.13 [1.00, 1.29]	5.66E-02	+?	*L3MBTL4*
				ACEi-induced cough	0.025	1.43 [1.31, 1.56]	6.10E-16	??++?+?+	
rs343240	18:6308062	T	C	Chronic dry cough	0.176	1.01 [0.97, 1.06]	5.64E-01	++	*L3MBTL4*
				ACEi-induced cough	0.178	0.86 [0.83, 0.89]	5.18E-18	------?-	
rs6062847	20:61322018	T	C	Chronic dry cough	0.135	1.16 [1.10, 1.21]	2.07E-09	+?	*NTSR1; SLCO4A1*
				ACEi-induced cough	0.138	1.19 [1.15, 1.24]	4.31E-24	-?+-++++	
rs5924943	X:150594104	T	C	Chronic dry cough	0.467	1.03 [1.00, 1.06]	4.40E-02	++	***VMA21***
				ACEi-induced cough	0.470	1.09 [1.07, 1.12]	7.96E-18	+?++-+?+	

Supplementary Table 8. Summary of protein-coding genes identified by variant-to-gene mapping.
Variant positions are reported in genome build GRCh37. QTL supporting variants described are colocalising hits identified by coloc.susie.

Trait	Gene symbol	Ensembl ID	Supporting analyses	Supporting variants	Novel gene?	PMID
Multi-trait	*KCNIP4*	ENSG00000185774	nearest; pathogenicity; PoPs	nearest(4:21393932:T:TAAG [4:21393932]); pathogenicity(4:21393932:T:TAAG [4:21393932]); PoPS(4:21393932:T:TAAG [4:21393932])	N	35751511; 26169577
Multi-trait	*MAPKAP1*	ENSG00000119487	nearest; pathogenicity; PoPs	nearest(rs7848821 [9:128251930]); pathogenicity(rs7046471 [9:128250409]); PoPS(rs7848821 [9:128251930])	Y	
Multi-trait	*PREP*	ENSG00000085377	nearest; PoPs; eQTL	nearest(rs7761208 [6:105776289]); PoPS(rs7761208 [6:105776289]); eQTL(rs11156437 [6:105778067])	N	35751511
ACEi-induced cough	*ALX1*	ENSG00000180318	nearest; PoPs	nearest(rs35336617 [12:85913015]); PoPS(rs35336617 [12:85913015])	Y	
ACEi-induced cough	*ATP23*	ENSG00000166896	nearest; eQTL	nearest(rs11172406 [12:58366606]); eQTL(rs4627125 [12:58372886])	Y	
Chronic dry cough	*CPEB2*	ENSG00000137449	nearest; PoPs	nearest(rs141733360 [4:14727977]); PoPS(rs141733360 [4:14727977])	Y	
Multi-trait	*L3MBTL4*	ENSG00000154655	nearest; PoPs	nearest(rs78598167 [18:6110865]); nearest(rs343240 [18:6308062]); PoPS(rs78598167 [18:6110865]); PoPS(rs343240 [18:6308062])	N	35751511
Multi-trait	*OR4C13*	ENSG00000258817	nearest; pathogenicity	nearest(rs112658458 [11:49790181]); pathogenicity(rs112658458 [11:49790181])	Y	
Multi-trait	*SIL1*	ENSG00000120725	nearest; eQTL	nearest(5:138404361:A:AAAG [5:138404361]); eQTL(rs12173085 [5:138480025]); eQTL(rs55650316 [5:138467292])	Y	
Multi-trait	*SRBD1*	ENSG00000068784	nearest; PoPs	nearest(rs1544730 [2:45588067]); PoPS(rs1544730 [2:45588067])	N	35751511
Multi-trait	*CTNNA1*	ENSG00000044115	PoPs	PoPS(5:138404361:A:AAAG [5:138404361])	Y	
ACEi-induced cough	*CYP27B1*	ENSG00000111012	PoPs	PoPS(rs11172406 [12:58366606])	Y	
ACEi-induced cough	*KCNA10*	ENSG00000143105	nearest	nearest(rs7518061 [1:111087944])	Y	
Multi-trait	*NTSR1*	ENSG00000101188	nearest	nearest(rs6062847 [20:61322018])	N	35751511
Multi-trait	*OR4C12*	ENSG00000221954	PoPs	PoPS(rs112658458 [11:49790181])	Y	
ACEi-induced cough	*RASSF9*	ENSG00000198774	eQTL	eQTL(rs2199514 [12:85991536])	Y	
ACEi-induced cough	*RBM15*	ENSG00000162775	PoPs	PoPS(rs7518061 [1:111087944])	Y	
Multi-trait	*SLCO4A1*	ENSG00000101187	PoPs	PoPS(rs6062847 [20:61322018])	N	35751511
Multi-trait	*VMA21*	ENSG00000160131	nearest	nearest(rs5924943 [23:150594104])	Y	

Supplementary Table 9. Variants with posterior inclusion probability (PIP) ≥10% identified through fine-mapping of multi-trait loci and additional independent loci from each single-trait GWAS.
Abbreviations: CI, confidence interval; OR, odds ratio.

Trait	Locus sentinel	Variant	Variant position (hg19)	Effect allele	Other allele	Effect allele frequency	Posterior inclusion probability	OR [95% CI]	*p*-value	Nearest gene	Annotation	CADD score
Multi-trait	rs1544730	rs1544730	2:45588067	A	G	0.223	0.375	1.11 [1.08, 1.13]	1.72E-16	*SRBD1*	Intergenic	5.231
Multi-trait	rs1544730	rs2112037	2:45591129	T	C	0.222	0.140	1.11 [1.08, 1.13]	5.36E-16	*SRBD1*	Intergenic	0.854
Multi-trait	rs1544730	rs3755070	2:45629316	T	C	0.230	0.131	1.1 [1.08, 1.13]	5.93E-16	*SRBD1*	Intronic	5.647
Multi-trait	4:21393932:T:TAAG	4:21393932:T:TAAG	4:21393932	T	TAAG	0.419	1.000	1.13 [1.11, 1.15]	4.97E-32	*KCNIP4*	Intronic	17.44
Multi-trait	5:138404361:A:AAAG	5:138404361:A:AAAG	5:138404361	A	AAAG	0.326	0.140	1.08 [1.05, 1.11]	2.62E-08	*SIL1*	Intronic	0.709
Multi-trait	rs7848821	rs7848821	9:128251930	A	G	0.320	0.393	1.08 [1.05, 1.10]	4.02E-12	*MAPKAP1*	Intronic	8.129
Multi-trait	rs7848821	rs7046471	9:128250409	T	C	0.685	0.346	0.93 [0.91, 0.95]	4.61E-12	*MAPKAP1*	Intronic	14
Multi-trait	rs7848821	rs11794506	9:128251275	A	G	0.323	0.133	1.08 [1.05, 1.10]	1.34E-11	*MAPKAP1*	Intronic	5.953
Multi-trait	rs112658458	rs112658458	11:49790181	A	G	0.967	0.189	1.19 [1.12, 1.26]	8.31E-09	*OR4C13*	Intronic	13.18
Multi-trait	rs112658458	rs145450510	11:49733467	T	C	0.033	0.170	0.84 [0.79, 0.89]	9.43E-09	*OR4C13*	Intronic	2.287
Multi-trait	rs112658458	rs72914105	11:49643260	T	C	0.967	0.140	1.19 [1.12, 1.26]	1.20E-08	*OR4C13*	Intronic	4.918
Multi-trait	rs112658458	rs182343842	11:49861001	T	C	0.032	0.140	0.84 [0.79, 0.89]	1.17E-08	*OR4C13*	Intergenic	1.105
Multi-trait	rs112658458	rs72914160	11:49716385	T	C	0.967	0.110	1.19 [1.12, 1.26]	1.43E-08	*OR4C13*	Intronic	1.953
Multi-trait	rs78598167	rs78598167	18:6110865	T	C	0.023	0.988	1.32 [1.23, 1.42]	8.36E-15	*L3MBTL4*	Intronic	1.213
Multi-trait	rs6062847	rs6062847	20:61322018	T	C	0.137	1.000	1.18 [1.15, 1.22]	1.10E-31	*NTSR1*	Intergenic	3.435
Multi-trait	rs5924943	rs5924943	23:150594104	T	C	0.469	0.292	1.07 [1.05, 1.09]	3.76E-16	*VMA21*	Intergenic	1.199
Multi-trait	rs5924943	rs12007715	23:150597999	A	C	0.472	0.113	1.07 [1.05, 1.09]	9.88E-16	*VMA21*	Intergenic	8.138
Chronic dry cough	rs141733360	rs141733360	4:14727977	A	C	0.009	0.997	0.51 [0.41, 0.64]	4.20E-09	*CPEB2*	ncRNA intronic	1.376
ACEi-induced cough	rs7518061	rs7518061	1:111087944	T	C	0.310	0.453	0.88 [0.84, 0.92]	1.85E-09	*KCNA10*	Intergenic	0.733
ACEi-induced cough	rs7518061	rs7526729	1:111087058	A	G	0.688	0.440	1.14 [1.09, 1.19]	2.16E-09	*KCNA10*	Intergenic	5.952
ACEi-induced cough	rs11172406	rs11172406	12:58366606	A	C	0.361	0.605	1.11 [1.07, 1.15]	1.73E-08	*ATP23*	Intergenic	3.193
ACEi-induced cough	rs11172406	rs6581173	12:58415737	T	C	0.365	0.141	1.11 [1.07, 1.15]	7.83E-08	*ATP23*	Intergenic	3.683
ACEi-induced cough	rs35336617	rs35336617	12:85913015	T	TC	0.703	0.328	0.87 [0.83, 0.91]	2.71E-09	*ALX1*	Intergenic	0.5
ACEi-induced cough	rs35336617	12:85838772_G_GA	12:85838772	G	GA	0.306	0.133	1.13 [1.09, 1.18]	6.85E-09	*ALX1*	Intergenic	0.099
ACEi-induced cough	rs35336617	rs548204060	12:86033240	CA	C	0.375	0.111	1.14 [1.09, 1.19]	8.02E-09	*RASSF9*	Intergenic	1.145

Supplementary Table 10. Genes with highest polygenic priority score (PoPs) within specified window of sentinels identified in the multi-trait GWAS and additional sentinels variants from each single-trait GWAS.

Trait	Sentinel ID	Position (hg19)	Gene symbol	Ensembl ID	Polygenic priority score	Window (bp)
Multi-trait	rs1544730	2:45588067	*SRBD1*	ENSG00000068784	0.5313	250000
Multi-trait	4:21393932:T:TAAG	4:21393932	*KCNIP4*	ENSG00000185774	0.7403	250000
Multi-trait	5:38404361:A:AAAG	5:138404361	*CTNNA1*	ENSG00000044115	0.4941	250000
Multi-trait	rs7761208	6:105776289	*PREP*	ENSG00000085377	0.5370	250000
Multi-trait	rs7848821	9:128251930	*MAPKAP1*	ENSG00000119487	0.6797	250000
Multi-trait	rs112658458	11:49790181	*OR4C12*	ENSG00000221954	0.0051	250000
Multi-trait	rs78598167	18:6110865	*L3MBTL4*	ENSG00000154655	0.7104	250000
Multi-trait	rs343240	18:6308062	*L3MBTL4*	ENSG00000154655	0.7104	250000
Multi-trait	rs6062847	20:61322018	*SLCO4A1*	ENSG00000101187	0.3588	250000
Chronic dry cough	rs141733360	4:14727977	*CPEB2*	ENSG00000137449	-0.1807	500000
ACEi-induced cough	rs7518061	1:111087944	*RBM15*	ENSG00000162775	0.4918	250000
ACEi-induced cough	rs11172406	12:58366606	*CYP27B1*	ENSG00000111012	0.7569	250000
ACEi-induced cough	rs35336617	12:85913015	*ALX1*	ENSG00000180318	-0.1156	250000

Supplementary Table 11. QTL colocalisation results.

Results are filtered for evidence for colocalisation between GWAS and expression/protein QTLs (H4) ≥0.7.

QTL	Trait	Locus sentinel	Locus sentinel position (hg19)	Dataset	Tissue	Gene	Ensembl ID	hit1	hit2	H0	H1	H2	H3	H4
eQTL	Multi-trait	rs11949916	5:138396277	GTeX_v8	Lung	*SIL1*	ENSG00000120725	5_138467292_C_G	5_138313146_A_G	5.86E-09	2.88E-06	5.56E-04	2.72E-01	7.28E-01
eQTL	Multi-trait	rs11949916	5:138396277	eQTLGen	Whole_Blood	*SIL1*	ENSG00000120725	5_138480025_A_G	5_138377550_C_G	0.00E+00	0.00E+00	2.67E-04	1.24E-01	8.76E-01
eQTL	Multi-trait	rs11949916	5:138396277	eQTLGen	Whole_Blood	*SIL1*	ENSG00000120725	5_138480025_A_G	5_138392482_A_G	0.00E+00	0.00E+00	3.48E-04	1.62E-01	8.38E-01
eQTL	Multi-trait	rs11949916	5:138396277	eQTLGen	Whole_Blood	*SIL1*	ENSG00000120725	5_138480025_A_G	5_138398172_A_G	0.00E+00	0.00E+00	3.61E-04	1.68E-01	8.32E-01
eQTL	Multi-trait	rs11949916	5:138396277	eQTLGen	Whole_Blood	*SIL1*	ENSG00000120725	5_138480025_A_G	5_138396277_G_T	0.00E+00	0.00E+00	2.19E-04	1.01E-01	8.99E-01
eQTL	Multi-trait	rs7761208	6:105776289	eQTLGen	Whole_Blood	*PREP*	ENSG00000085377	6_105778067_C_T	6_105799455_C_T	2.56E-25	1.54E-13	6.26E-14	3.57E-02	9.64E-01
eQTL	ACEi-induced cough	rs11172406	12:58366606	GTeX_v8	Whole_Blood	*ATP23*	ENSG00000166896	12_58372886_C_T	12_58458032_A_G	8.11E-15	2.51E-13	7.61E-03	2.34E-01	7.59E-01
eQTL	ACEi-induced cough	rs12230791	12:85919981	GTeX_v8	Adrenal_Gland	*RASSF9*	ENSG00000198774	12_85991536_A_G	12_86016913_A_G	5.40E-10	3.97E-06	1.59E-05	1.15E-01	8.85E-01

Supplementary Table 12. Functions of mapped genes.

Gene Symbol	Gene Name	Description
ALX1	ALX Homeobox 1	DNA binding transcription factor likely to regulate expression of genes involved in mesenchyme-derived craniofacial structure development[60]. Associated with Frontonasal Dysplasia 3[61,62].
ATP23	ATP23 Metallopeptidase And ATP Synthase Assembly Factor Homolog	Encodes a metallopeptidase involved in DNA repair through binding via the DNA-binding subunit of DNA-dependent protein kinase, and also has a role in mitochondrial protein processing and ATP synthase complex assembly[60]. ATP23 is known to be amplified in glioblastomas[60], and is highly expressed in the reproductive system[62].
CPEB2	Cytoplasmic Polyadenylation Element Binding Protein 2	Important for transition from metaphase to anaphase in the cell cycle[62]. Involved in regulation of cytoplasmic translation[62].
CTNNA1	Catenin alpha 1	Member of the catenin family which is widely expressed across anatomical systems[62], and is most well-known for its role in cell-cell adhesion by connecting cadherins to the actin cytoskeleton[60]. Additional functions include cell migration, regulation of apoptosis, axon regeneration and integrin-mediated signalling. CTNNA1 variants have been associated with macular dystrophy (autosomal dominant inheritance)[61], and other eye diseases including butterfly-shaped pigment dystrophy[62].
CYP27B1	Cytochrome P450 Family 27 Subfamily B Member 1	Member of the cytochrome P450 family localised to the mitochondrial membrane[60,62]. Synthesises the active form of vitamin D3 which is involved in calcium homeostasis[60]. Associated with Vitamin D-dependent rickets, type I[61].
KCNA10	Potassium Voltage-Gated Channel Subfamily A Member 10	Intronless gene encoding a member of the voltage-gated potassium channel complex subfamily[60]. Mediates potassium ion transmembrane transport across plasma membranes[62].
KCNIP4	Potassium voltage-gated channel interacting protein 4	Integral component of voltage-gated potassium channel complexes predominantly expressed in the brain[60,62]. Involved in the regulation of potassium ion transmembrane transport and excitability in neurons and cardomycotyes[63]. Also involved in protein localisation to the plasma membrane[62].
L3MBTL4	L3MBTL histone methyl-lysine binding protein 4	Negatively regulates transcription through chromatin organisation and histone binding activity and has high baseline expression in the immune and hematopoietic systems[60,62]. Following evidence of association between L3MBTL4 and hypertension[64], in vivo analyses showed that L3MBTL4 induces the proliferation and remodelling of vascular smooth muscle cells via the MAPK pathway, resulting in hypertension[65].
MAPKAP1	MAPK associated protein 1	Subunit of mTORC2 which is involved in a range of biological processes via the mTOR signalling pathway[62]. With widespread expression across anatomical systems, MAPKAP1 regulates cell growth, autophagy, and survival in response to stimuli (e.g. hormones, growth factors and stress)[60]. Dysfunction of mTORC2 has been linked to neurodegenerative disease, epilepsy and cancer[66].
NTSR1	Neurotensin receptor 1	High baseline expression in the nervous system and encodes a G-protein coupled receptor which is implicated in many functions modulated by neurotensin, a vasoactive peptide which binds with high affinity to NTSR1[60,62]. Functions include regulation of neuropeptide signalling pathways, neurotransmitter secretion (specifically, gamma-aminobutyric acid and glutamate) and synaptic transmission[62].
OR4C12	Olfactory receptor family 4 subfamily C member 12	G-protein coupled receptor located in the plasma membrane which is involved in smell perception via the transduction of odorant signals upon detection of specific chemical stimuli[60,62]. Predominantly expressed in the olfactory sensory neurons of the nasal epithelium[67].
OR4C13	Olfactory receptor family 4 subfamily C member 13	
PREP	Prolyl endopeptidase	Cytosolic prolyl endopeptidase involved in the maturation and degradation of peptide hormones and neuropeptides[60], including angiotensin II, bradykinin and oxytocin[68,69]. Has high baseline expression in the digestive system[62], and is also hypothesised to be involved in the release of Ac-SDKP from its precursor thymosin-β4 which has anti-inflammatory and anti- fibrotic effects[70]. This is supported by the relationship between PREP and inflammatory disease, including neurodegeneration, cancer and COPD[71].
RASSF9	Ras Association Domain Family Member 9	Encoding protein is localised to endosomes and has a role in intracellular and endosomal transport, protein targeting and signal transduction[62].
RBM15	RNA Binding Motif Protein 15	Member of the SPEN family of proteins[60]. Involved in a wide range of biological processes, including RNA methylation, transcription regulation, alternative splicing regulation, and differentiation of myeloid cells and megakaryocytes[62].
SIL1	SIL1 nucleotide exchange factor	Glycoprotein which is widely expressed across anatomical systems[62] and involved in endoplasmic reticulum-based protein translocation and folding[60,62]. Homozygous mutations in SIL1 have been associated with Marinesco-Sjogren syndrome[61], characterised by ataxia, early onset cataracts, myopathy, muscle weakness, hypotonia and intellectual disability[72].
SLCO4A1	Solute Carrier Organic Anion Transporter Family Member 4A1	Highly expressed in the respiratory system[62] and located in the plasma membrane[60]. Involved in the transport of various molecules, including prostaglandins, thyroid hormones and organic anions[62].
SRBD1	S1 RNA Binding Domain 1	Likely to be a ribosomal component involved in translation[60,62].
VMA21	Vacuolar ATPase assembly factor VMA21	Chaperone for the assembly of lysosomal vascular ATPase (an enzyme which regulates intracellular pH) and has a high baseline expression in the immune and hematopoietic systems[60,62]. Mutations in VMA21 have been linked to the X-linked recessive disease, myopathy with excessive autophagy[73], and non-alcoholic fatty liver disease[74].

Supplementary Table 13. Druggability.

Gene	Drug name	Indication (phase)	ChEMBL ID	Experimental Factor Ontology Terms	Interaction type	Drug description	Approval Year
KCNA10	DALFAMPRIDINE	Trauma, Nervous System(2); Guillain-Barre Syndrome(2); Optic Neuritis(0); Muscle Spasticity(3); Multiple Sclerosis, Relapsing-Remitting(3); Cerebral Palsy(1); Multiple Sclerosis(4); Motor Neuron Disease(1); Stroke(3); Muscular Atrophy, Spinal(2); Spinal Cord Injuries(3); Renal Insufficiency(1); Multiple Sclerosis, Chronic Progressive(3); Ischemic Stroke(3); Sleep Apnea, Obstructive(2)	CHEMBL284348	nervous system injury; Guillain-Barre syndrome; optic neuritis; Spasticity; relapsing-remitting multiple sclerosis; cerebral palsy; multiple sclerosis; motor neuron disease; stroke; Proximal spinal muscular atrophy type 3; Spinal cord injury; Renal insufficiency; secondary progressive multiple sclerosis; Ischemic stroke; obstructive sleep apnea	blocker	Small molecule potassium channel blocker used to improve motor function.	2010
KCNA10	NERISPIRDINE	Multiple Sclerosis(2); Spinal Cord Injuries(2)	CHEMBL2107762	multiple sclerosis; Spinal cord injury	blocker		NA
NTSR1	REMINERTANT	Small Cell Lung Carcinoma(2)	CHEMBL506981	small cell lung carcinoma	antagonist		NA
KCNA10	TEDISAMIL	Arrhythmias, Cardiac(0); Atrial Fibrillation(3); Atrial Flutter(3)	CHEMBL2111110	cardiac arrhythmia; atrial fibrillation; atrial flutter	blocker		NA

Supplementary Table 14. Sentinel variant PheWAS results (false discovery rate [FDR] <0.01) using DeepPheWAS.
Mapped genes in bold are novel. *The chronic dry cough phenotype denoted here has the same definition as 'chronic dry cough' described above and utilised in our genetic analyses (**Supplementary Methods**). *Abbreviations*: EA, effect allele; MA, minor allele; MAF, minor allele frequency; OA, other allele; OR, odds ratio; SE, standard error.

Trait	Sentinel ID	Position (hg19)	EA	OA	MA	MAF	Trait description	Ancestry	N	FDR	*p*-value	OR	Beta	L95	U95	SE	Direction	Mapped gene(s)
Multi-trait	rs343240	18:6308062	C	T	T	0.194	Other specified cardiac dysrhythmias	SAS	7854	7.74E-03	7.96E-06	0.578	NA	0.454	0.735	0.123	-	*L3MBTL4*
Multi-trait	rs6062847	20:61322018	T	C	T	0.136	Chronic dry cough*	EUR	96223	1.12E-07	5.76E-11	1.163	NA	1.111	1.216	0.023	+	*NTSR1; SLCO4A1*
Multi-trait	rs6062847	20:61322018	T	C	T	0.136	Cough on most days	EUR	105125	4.24E-06	4.37E-09	1.111	NA	1.072	1.150	0.018	+	*NTSR1; SLCO4A1*
Multi-trait	rs5924943	X:150594104	T	C	T	0.467	IGF-1	EUR	374381	3.17E-06	1.64E-09	NA	-0.011	-0.015	-0.008	0.002	-	***VMA21***
Chronic dry cough	rs141733360	4:14727977	C	A	A	0.009	Chronic dry cough*	EUR	96223	1.98E-04	1.02E-07	1.894	NA	1.497	2.397	0.120	+	***CPEB2***
Chronic dry cough	rs141733360	4:14727977	C	A	A	0.009	Cough on most days	EUR	105125	2.18E-03	2.24E-06	1.469	NA	1.253	1.723	0.081	+	***CPEB2***
ACEi-induced cough	rs11172406	12:58366606	A	C	A	0.337	Sitting height	EUR	392979	5.17E-04	5.34E-07	NA	-0.009	-0.013	-0.005	0.002	-	***ATP23; CYP27B1***
ACEi-induced cough	rs11172406	12:58366606	A	C	A	0.337	Urate	EUR	376291	7.85E-14	4.05E-17	NA	-0.017	-0.021	-0.013	0.002	-	***ATP23; CYP27B1***
ACEi-induced cough	rs11172406	12:58366606	A	C	A	0.337	Urea	EUR	376503	6.21E-03	9.60E-06	NA	-0.010	-0.015	-0.006	0.002	-	***ATP23; CYP27B1***

Supplementary Table 15. Open Targets Genetics 'GWAS lead variants' results.
Mapped genes in bold are novel. *Abbreviations*: CI, confidence interval; OR, odds ratio; SE, standard error.

Trait	Sentinel variant	Query variant	Open Targets lead variant	Open Targets lead variant tested allele	r^2	Study ID	Study Trait	*p*-value	Beta	OR	95% CI	Author, Year (PMID)	Study N	Direction	Mapped gene(s)
Multi-trait	4:21393932:T:TAAG	rs7675300	rs1495509	C	0.997	GCST003027	Cough in response to angiotensin-converting enzyme inhibitor drugs	2.00E-09		1.23	(1.1, 1.3)	Mosley JD, 2016 (26169577)	12311	+	*KCNIP4*
Multi-trait	5:138404361:A:AAAG	rs11949916	rs11242445	T	0.864	GCST90013473	Biological sex	5.00E-10	-0.0117		(−0.015, −0.0080)	Pirastu N, 2021 (33888908)	2462132	-	***CTNNA1; SIL1***
Multi-trait	rs6062847	rs6062847	rs6062847	T	1.000	NEALE2_22502	Cough on most days	1.38E-09		1.13	(1.1, 1.2)	UKB Neale v2	91787	+	*NTSR1; SLCO4A1*
ACEi-induced cough	rs11172406	rs11172406	rs12578279	G	0.877	GCST006569	Self-reported math ability (MTAG) [MTAG]	5.00E-19				Lee JJ, 2018 (30038396)	670471	NA	***ATP23; CYP27B1***
ACEi-induced cough	rs11172406	rs11172406	rs12578279	G	0.877	GCST006573	Self-reported math ability	1.00E-16				Lee JJ, 2018 (30038396)	564698	NA	***ATP23; CYP27B1***
ACEi-induced cough	rs11172406	rs11172406	rs4760364	G	0.877	GCST006568	Highest math class taken (MTAG) [MTAG]	6.00E-18	-0.0152		(0.012, 0.019)	Lee JJ, 2018 (30038396)	811539	-	***ATP23; CYP27B1***
ACEi-induced cough	rs11172406	rs11172406	rs10877067	T	0.877	GCST90025965	Urate levels	2.60E-29	-0.0224		(0.018, 0.026)	Barton AR, 2021 (34226706)	437354	-	***ATP23; CYP27B1***

Supplementary Table 16. Sentinel variant Respiratory PheWAS results (false discovery rate [FDR] <0.01).
Mapped genes in bold are novel. *Abbreviations*: EA, effect allele; OA, other allele OR, odds ratio; SE, standard error.

Trait	Sentinel ID	Position (hg19)	EA	OA	Clinical phenotype	Clinical subtype	Cohort	Ancestry	Author (Year)	PMID	N	FDR	*p*-value	OR	Beta	L95	U95	SE	Direction	Mapped gene(s)
Multi-trait	rs6062847	20:61322018	T	C	Asthma	Asthma	GBMI	Multi	Tsuo (2022)	36778051	1800785	4.43E-04	1.70E-05	1.026	0.0261	1.014	1.039	0.0061	+	*NTSR1; SLCO4A1*
Multi-trait	rs7848821	9:128251930	A	G	Asthma	Asthma	GBMI	Multi	Tsuo (2022)	36778051	1800785	9.62E-03	3.70E-04	1.016	0.0158	1.007	1.025	0.0044	+	***MAPKAP1***

Supplementary Table 17. Sensitivity analysis excluding short-term chronic dry cough.
Mapped genes in bold are novel. Direction columns have the following order: UK Biobank-European, UK Biobank-South Asian for chronic dry cough; UK Biobank-African, UK Biobank-East Asian, UK Biobank-European, UK Biobank-South Asian, EXCEED-European, Genes & Health-South Asian, eMERGE Network, Copenhagen Hospital Biobank for ACEi-induced cough; chronic dry cough, ACEi-induced cough for multi-trait analysis. *Abbreviations*: EAF, effect allele frequency; CI, confidence interval; OR, odds ratio.

Trait	Sentinel ID	Position (hg19)	Effect Allele	Other Allele	Primary analysis						Sensitivity analysis (excluding short-term chronic dry cough)						Mapped gene(s)
					EAF	OR [95% CI]	*p*-value	*N* cases	*N* controls	Direction	EAF	OR [95% CI]	*p*-value	*N* cases	*N* controls	Direction	
Multi-trait	rs1544730	2:45588067	A	G	0.223	1.11 [1.08, 1.13]	1.72E-16	27515	145377	++	0.224	1.11 [1.08, 1.14]	3.80E-16	26753	145377	++	*SRBD1*
Multi-trait	4:21393932:T:TAAG	4:21393932	T	TAAG	0.419	1.13 [1.11, 1.15]	4.97E-32	27563	145737	++	0.420	1.13 [1.11, 1.16]	4.97E-32	26801	145737	++	*KCNIP4*
Multi-trait	5:138404361:A:AAAG	5:138404361	A	AAAG	0.326	1.08 [1.05, 1.11]	2.62E-08	12894	122015	++	0.326	1.09 [1.06, 1.12]	2.16E-08	12132	122015	++	***CTNNA1; SIL1***
Multi-trait	rs7761208	6:105776289	T	C	0.788	0.89 [0.87, 0.91]	1.72E-20	27531	145621	--	0.787	0.89 [0.86, 0.91]	2.81E-21	26769	145621	--	*PREP*
Multi-trait	rs7848821	9:128251930	A	G	0.320	1.08 [1.05, 1.10]	4.02E-12	29158	151222	++	0.320	1.08 [1.06, 1.10]	2.52E-12	28396	151222	++	***MAPKAP1***
Multi-trait	rs112658458	11:49790181	A	G	0.967	1.19 [1.12, 1.26]	8.31E-09	27117	142799	++	0.967	1.19 [1.13, 1.27]	4.77E-09	26363	142799	++	***OR4C12; OR4C13***
Multi-trait	rs78598167	18:6110865	T	C	0.023	1.33 [1.24, 1.43]	8.36E-15	27295	143722	++	0.023	1.34 [1.24, 1.44]	5.05E-15	26541	143722	++	*L3MBTL4*
Multi-trait	rs343240	18:6308062	T	C	0.178	0.91 [0.89, 0.94]	4.90E-11	27563	145737	+-	0.178	0.91 [0.88, 0.93]	4.75E-12	26801	145737	-+	*L3MBTL4*
Multi-trait	rs6062847	20:61322018	T	C	0.137	1.18 [1.15, 1.22]	1.10E-31	29066	150277	++	0.137	1.19 [1.15, 1.22]	9.81E-32	28312	150277	++	*NTSR1; SLCO4A1*
Multi-trait	rs5924943	X:150594104	T	C	0.469	1.07 [1.05, 1.09]	3.76E-16	27531	145621	++	0.469	1.07 [1.06, 1.09]	8.21E-17	26769	145621	++	***VMA21***
Chronic dry cough	rs141733360	4:14727977	A	C	0.009	0.51 [0.41, 0.64]	4.20E-09	7575	87824	-?	0.009	0.46 [0.37, 0.59]	3.45E-10	6821	87824	-?	***CPEB2***
ACEi-induced cough	rs7518061	1:111087944	T	C	0.310	0.88 [0.84, 0.92]	1.85E-09	6254	41212	---+--??	0.310	0.88 [0.84, 0.92]	1.85E-09	6254	41212	---+--??	***KCNA10; RBM15***
ACEi-induced cough	rs11172406	12:58366606	A	C	0.361	1.11 [1.07, 1.15]	1.73E-08	7849	46697	+-+++++?	0.361	1.11 [1.07, 1.15]	1.73E-08	7849	46697	+-+++++?	***ATP23; CYP27B1***
ACEi-induced cough	rs35336617	12:85913015	T	TC	0.703	0.87 [0.83, 0.91]	2.71E-09	5259	33362	?---????	0.703	0.87 [0.83, 0.91]	2.71E-09	5259	33362	?---????	***ALX1; RASSF9***

Supplementary Table 18. Sensitivity analysis excluding ACEi-induced cough cases with a cough code within 12 months of switching to an ARB.
Mapped genes in bold are novel. In the primary analysis, direction column has the following order: UK Biobank-European, UK Biobank-South Asian for chronic dry cough; UK Biobank-African, UK Biobank-East Asian, UK Biobank-European, UK Biobank-South Asian, EXCEED-European, Genes & Health-South Asian, eMERGE Network, Copenhagen Hospital Biobank for ACEi-induced cough; chronic dry cough, ACEi-induced cough for multi-trait analysis. In the UK Biobank only (excluding cough code after switch to ARB) sensitivity analysis, direction column has the following order: UK Biobank-European, UK Biobank-South Asian for chronic dry cough; UK Biobank-African, UK Biobank-East Asian, UK Biobank-European, UK Biobank-South Asian; chronic dry cough, ACEi-induced cough for multi-trait analysis. *Abbreviations*: EAF, effect allele frequency; CI, confidence interval; OR, odds ratio.

Trait	Sentinel ID	Position (hg19)	Effect Allele	Other Allele	Primary analysis						Sensitivity analysis (excluding cough code after switch to ARB)						Mapped gene(s)
					EAF	OR [95% CI]	*p*-value	*N* cases	*N* controls	Direction	EAF	OR [95% CI]	*p*-value	*N* cases	*N* controls	Direction	
Multi-trait	rs1544730	2:45588067	A	G	0.223	1.11 [1.08, 1.13]	1.72E-16	27515	145377	++	0.210	1.09 [1.06, 1.13]	1.59E-07	12349	122015	++	*SRBD1*
Multi-trait	4:21393932:T:TAAG	4:21393932	T	TAAG	0.419	1.13 [1.11, 1.15]	4.97E-32	27563	145737	++	0.411	1.13 [1.10, 1.16]	2.41E-17	12349	122015	++	*KCNIP4*
Multi-trait	5:138404361:A:AAAG	5:138404361	A	AAAG	0.326	1.08 [1.05, 1.11]	2.62E-08	12894	122015	++	0.326	1.08 [1.05, 1.11]	1.31E-07	12322	121899	++	***CTNNA1; SIL1***
Multi-trait	rs7761208	6:105776289	T	C	0.788	0.89 [0.87, 0.91]	1.72E-20	27531	145621	--	0.802	0.88 [0.85, 0.91]	4.36E-15	12366	122259	--	*PREP*
Multi-trait	rs7848821	9:128251930	A	G	0.320	1.08 [1.05, 1.10]	4.02E-12	29158	151222	++	0.311	1.08 [1.05, 1.11]	2.48E-07	12393	122375	++	***MAPKAP1***
Multi-trait	rs112658458	11:49790181	A	G	0.967	1.19 [1.12, 1.26]	8.31E-09	27117	142799	++	0.966	1.14 [1.06, 1.24]	6.15E-04	12112	120147	++	***OR4C12; OR4C13***
Multi-trait	rs78598167	18:6110865	T	C	0.023	1.33 [1.24, 1.43]	8.36E-15	27295	143722	++	0.019	1.40 [1.27, 1.54]	6.27E-12	12262	121070	++	*L3MBTL4*
Multi-trait	rs343240	18:6308062	T	C	0.178	0.91 [0.89, 0.94]	4.90E-11	27563	145737	+-	0.178	0.92 [0.89, 0.96]	7.25E-06	12393	122375	-+	*L3MBTL4*
Multi-trait	rs6062847	20:61322018	T	C	0.137	1.18 [1.15, 1.22]	1.10E-31	29066	150277	++	0.135	1.18 [1.14, 1.23]	3.50E-18	12306	121430	++	*NTSR1; SLCO4A1*
Multi-trait	rs5924943	X:150594104	T	C	0.469	1.07 [1.05, 1.09]	3.76E-16	27531	145621	++	0.469	1.07 [1.05, 1.09]	5.19E-09	12366	122259	++	***VMA21***
Chronic dry cough	rs141733360	4:14727977	A	C	0.009	0.51 [0.41, 0.64]	4.20E-09	7575	87824	-?	0.009	0.51 [0.41, 0.64]	4.20E-09	7575	87824	-?	***CPEB2***
ACEi-induced cough	rs7518061	1:111087944	T	C	0.310	0.88 [0.84, 0.92]	1.85E-09	6254	41212	---+--??	0.303	0.88 [0.84, 0.93]	6.18E-07	4758	33722	---+	***KCNA10; RBM15***
ACEi-induced cough	rs11172406	12:58366606	A	C	0.361	1.11 [1.07, 1.15]	1.73E-08	7849	46697	+-+++++?	0.340	1.11 [1.06, 1.16]	1.26E-05	4758	33722	+-++	***ATP23; CYP27B1***
ACEi-induced cough	rs35336617	12:85913015	T	TC	0.703	0.87 [0.83, 0.91]	2.71E-09	5259	33362	?---????	0.704	0.87 [0.83, 0.92]	4.48E-08	4714	33362	?---	***ALX1; RASSF9***

Supplementary Table 19. UK Biobank sensitivity analysis excluding asthma.
Mapped genes in bold are novel. In the primary analysis, direction column has the following order: UK Biobank-European, UK Biobank-South Asian for chronic dry cough; UK Biobank-African, UK Biobank-East Asian, UK Biobank-European, UK Biobank-South Asian, EXCEED-European, Genes & Health-South Asian, eMERGE Network, Copenhagen Hospital Biobank for ACEi-induced cough; chronic dry cough, ACEi-induced cough for multi-trait analysis. In the UK Biobank only (excluding asthma) sensitivity analysis, direction column has the following order: UK Biobank-European, UK Biobank-South Asian for chronic dry cough; UK Biobank-African, UK Biobank-East Asian, UK Biobank-European, UK Biobank-South Asian; chronic dry cough, ACEi-induced cough for multi-trait analysis. *Abbreviations*: EAF, effect allele frequency; CI, confidence interval; OR, odds ratio.

Trait	Sentinel ID	Position (hg19)	Effect Allele	Other Allele	Primary analysis						Sensitivity analysis in UK Biobank only (excluding asthma)						Mapped gene(s)
					EAF	OR [95% CI]	*p*-value	*N* cases	*N* controls	Direction	EAF	OR [95% CI]	*p*-value	*N* cases	*N* controls	Direction	
Multi-trait	rs1544730	2:45588067	A	G	0.223	1.11 [1.08, 1.13]	1.72E-16	27515	145377	++	0.210	1.09 [1.06, 1.13]	5.98E-07	10539	110137	++	*SRBD1*
Multi-trait	4:21393932:T:TAAG	4:21393932	T	TAAG	0.419	1.13 [1.11, 1.15]	4.97E-32	27563	145737	++	0.411	1.13 [1.09, 1.16]	2.07E-15	10584	110442	++	*KCNIP4*
Multi-trait	5:138404361:A:AAAG	5:138404361	A	AAAG	0.326	1.08 [1.05, 1.11]	2.62E-08	12894	122015	++	0.326	1.07 [1.04, 1.11]	6.16E-06	10539	110137	++	***CTNNA1; SIL1***
Multi-trait	rs7761208	6:105776289	T	C	0.788	0.89 [0.87, 0.91]	1.72E-20	27531	145621	--	0.802	0.88 [0.85, 0.91]	4.14E-12	10555	110339	--	*PREP*
Multi-trait	rs7848821	9:128251930	A	G	0.320	1.08 [1.05, 1.10]	4.02E-12	29158	151222	++	0.311	1.09 [1.06, 1.12]	6.28E-08	10584	110442	++	***MAPKAP1***
Multi-trait	rs112658458	11:49790181	A	G	0.967	1.19 [1.12, 1.26]	8.31E-09	27117	142799	++	0.966	1.18 [1.09, 1.28]	1.12E-04	10326	108517	++	***OR4C12; OR4C13***
Multi-trait	rs78598167	18:6110865	T	C	0.023	1.33 [1.24, 1.43]	8.36E-15	27295	143722	++	0.019	1.43 [1.29, 1.59]	1.34E-11	10464	109278	++	*L3MBTL4*
Multi-trait	rs343240	18:6308062	T	C	0.178	0.91 [0.89, 0.94]	4.90E-11	27563	145737	+-	0.178	0.91 [0.87, 0.94]	1.24E-06	10584	110442	+-	*L3MBTL4*
Multi-trait	rs6062847	20:61322018	T	C	0.137	1.18 [1.15, 1.22]	1.10E-31	29066	150277	++	0.135	1.20 [1.15, 1.25]	1.80E-18	10509	109583	++	*NTSR1; SLCO4A1*
Multi-trait	rs5924943	X:150594104	T	C	0.469	1.07 [1.05, 1.09]	3.76E-16	27531	145621	++	0.468	1.06 [1.04, 1.09]	1.03E-06	10555	110339	++	***VMA21***
Chronic dry cough	rs141733360	4:14727977	A	C	0.009	0.51 [0.41, 0.64]	4.20E-09	7575	87824	-?	0.009	0.58 [0.46, 0.74]	1.05E-05	6202	81172	-?	***CPEB2***
ACEi-induced cough	rs7518061	1:111087944	T	C	0.310	0.88 [0.84, 0.92]	1.85E-09	6254	41212	---+--??	0.304	0.87 [0.83, 0.92]	2.86E-07	4336	28514	---+	***KCNA10; RBM15***
ACEi-induced cough	rs11172406	12:58366606	A	C	0.361	1.11 [1.07, 1.15]	1.73E-08	7849	46697	+-+++++?	0.340	1.12 [1.07, 1.18]	2.80E-06	4336	28514	+-++	***ATP23; CYP27B1***
ACEi-induced cough	rs35336617	12:85913015	T	TC	0.703	0.87 [0.83, 0.91]	2.71E-09	5259	33362	?---????	0.704	0.87 [0.82, 0.91]	4.24E-08	4291	28209	?--+	***ALX1; RASSF9***

Supplementary Table 20. Sex-stratified association testing and sex-interaction testing of sentinel variants in UK Biobank (European only).
Mapped genes in bold are novel. *Abbreviations:* CI, confidence interval; OR, odds ratio.

Sentinel ID	Position (hg19)	Effect allele	Other allele	Trait	Sex-stratified association testing						Sex-interaction testing		Mapped gene
					Sex	*N* cases	*N* controls	Effect allele frequency	OR [95% CI]	*p*-value	OR [95% CI]	*p*-value	
rs1544730	2:45588067	A	G	Multi-trait	Both sexes	12624	120147	0.208	1.10 [1.06, 1.13]	2.95E-08	0.98 [0.91, 1.04]	4.63E-01	*SRBD1*
					Male	5374	56703	0.208	1.08 [1.03, 1.14]	1.47E-03			
					Female	7250	63444	0.209	1.11 [1.06, 1.16]	3.54E-06			
4:21393932:T:TAAG	4:21393932	T	TAAG	Multi-trait	Both sexes	12624	120147	0.410	1.13 [1.10, 1.16]	1.73E-18	0.96 [0.91, 1.01]	1.41E-01	*KCNIP4*
					Male	5374	56703	0.410	1.11 [1.06, 1.16]	7.31E-07			
					Female	7250	63444	0.411	1.14 [1.10, 1.19]	3.64E-13			
5:138404361:A:AAAG	5:138404361	A	AAAG	Multi-trait	Both sexes	12624	120147	0.326	1.08 [1.05, 1.12]	2.28E-08	0.97 [0.92, 1.03]	3.78E-01	***CTNNA1; SIL1***
					Male	5374	56703	0.325	1.07 [1.02, 1.12]	2.60E-03			
					Female	7250	63444	0.327	1.10 [1.06, 1.14]	1.53E-06			
rs7761208	6:105776289	T	C	Multi-trait	Both sexes	12624	120147	0.807	0.88 [0.85, 0.91]	1.37E-14	1.06 [0.99, 1.13]	1.19E-01	*PREP*
					Male	5374	56703	0.806	0.90 [0.85, 0.95]	3.43E-05			
					Female	7250	63444	0.807	0.86 [0.82, 0.90]	3.79E-11			
rs7848821	9:128251930	A	G	Multi-trait	Both sexes	12624	120147	0.309	1.08 [1.05, 1.11]	5.36E-08	1.02 [0.96, 1.08]	5.43E-01	***MAPKAP1***
					Male	5374	56703	0.309	1.09 [1.05, 1.14]	5.02E-05			
					Female	7250	63444	0.310	1.07 [1.03, 1.12]	2.44E-04			
rs112658458	11:49790181	A	G	Multi-trait	Both sexes	12624	120147	0.966	1.16 [1.07, 1.25]	2.01E-04	1.08 [0.92, 1.26]	3.67E-01	***OR4C12; OR4C13***
					Male	5374	56703	0.965	1.20 [1.07, 1.35]	1.80E-03			
					Female	7250	63444	0.966	1.12 [1.01, 1.23]	3.03E-02			
rs78598167	18:6110865	T	C	Multi-trait	Both sexes	12624	120147	0.018	1.38 [1.25, 1.52]	5.54E-11	0.76 [0.63, 0.92]	4.69E-03	*L3MBTL4*
					Male	5374	56703	0.017	1.18 [1.02, 1.37]	2.86E-02			
					Female	7250	63444	0.018	1.52 [1.34, 1.72]	2.62E-11			
rs343240	18:6308062	T	C	Multi-trait	Both sexes	12624	120147	0.176	0.92 [0.89, 0.95]	4.22E-06	1.01 [0.94, 1.08]	8.02E-01	*L3MBTL4*
					Male	5374	56703	0.175	0.92 [0.87, 0.97]	2.02E-03			
					Female	7250	63444	0.178	0.92 [0.88, 0.96]	5.61E-04			
rs6062847	20:61322018	T	C	Multi-trait	Both sexes	12624	120147	0.135	1.19 [1.15, 1.24]	8.64E-20	0.97 [0.90, 1.04]	3.91E-01	*NTSR1; SLCO4A1*
					Male	5374	56703	0.135	1.17 [1.11, 1.24]	6.39E-08			
					Female	7250	63444	0.135	1.21 [1.15, 1.27]	1.83E-13			
rs5924943	X:150594104	T	C	Multi-trait	Both sexes	12624	120147	0.465	1.07 [1.05, 1.09]	3.14E-09	0.99 [0.95, 1.04]	6.59E-01	***VMA21***
					Male	5374	56703	0.467	1.07 [1.04, 1.10]	6.14E-06			
					Female	7250	63444	0.464	1.07 [1.03, 1.11]	1.30E-04			

rs141733360	4:14727977	A	C	Chronic dry cough	Both sexes	7575	87824	0.009	0.51 [0.41, 0.64]	4.20E-09	0.72 [0.42, 1.22]	2.20E-01	***CPEB2***
					Male	3100	37468	0.009	0.43 [0.30, 0.61]	4.25E-06			
					Female	4475	50356	0.009	0.58 [0.44, 0.77]	1.58E-04			
rs7518061	1:111087944	T	C	ACEi-induced cough	Both sexes	5049	32323	0.301	0.87 [0.83, 0.91]	1.54E-08	1.01 [0.91, 1.11]	8.60E-01	***KCNA10; RBM15***
					Male	2274	19235	0.302	0.88 [0.82, 0.94]	1.93E-04			
					Female	2775	13088	0.301	0.87 [0.81, 0.92]	1.79E-05			
rs11172406	12:58366606	A	C	ACEi-induced cough	Both sexes	5049	32323	0.336	1.12 [1.07, 1.17]	8.45E-07	0.93 [0.98, 1.17]	1.42E-01	***ATP23; CYP27B1***
					Male	2274	19235	0.336	1.16 [1.09, 1.24]	1.06E-05			
					Female	2775	13088	0.336	1.09 [1.02, 1.16]	9.04E-03			
rs35336617	12:85913015	T	TC	ACEi-induced cough	Both sexes	5049	32323	0.704	0.87 [0.83, 0.91]	2.82E-09	0.97 [0.94, 1.13]	5.53E-01	***ALX1; RASSF9***
					Male	2274	19235	0.704	0.88 [0.82, 0.94]	2.19E-04			
					Female	2775	13088	0.704	0.86 [0.80, 0.91]	3.37E-06			

Supplementary Table 21. Polygenic score PheWAS results (false discovery rate [FDR] <0.01) using DeepPheWAS.
Abbreviations: OR, odds ratio; SE, standard error.

PheWAS ID	Phenotype	FDR	*p*-value	OR	Beta	L95	U95	SE	Phenotype group	Phenotype group (narrow)	Direction
Q0524	Sex hormone binding globulin	2.13E-12	4.01E-16	NA	-0.013	-0.016	-0.0096	0.0016	Quantitative Measure	Blood Biochemistry	-
Q0002	Sitting height	5.97E-09	3.37E-12	NA	0.0084	0.006	0.011	0.0012	Quantitative Measure	Anthropometry	+
Q0529	Urate	7.93E-08	5.97E-11	NA	0.009	0.0063	0.012	0.0014	Quantitative Measure	Blood Biochemistry	+
Q0008	Whole body fat-free mass	5.13E-06	5.80E-09	NA	0.0058	0.0039	0.0078	0.001	Quantitative Measure	Anthropometry	+
Q0010	Basal metabolic rate	5.13E-06	5.14E-09	NA	0.0061	0.0041	0.0081	0.001	Quantitative Measure	Anthropometry	+
Q0001	Standing height	7.06E-06	9.31E-09	NA	0.0064	0.0042	0.0086	0.0011	Quantitative Measure	Anthropometry	+
Q0003	Weight	1.50E-04	2.54E-07	NA	0.0073	0.0045	0.01	0.0014	Quantitative Measure	Anthropometry	+
Q1000	Multi site chronic pain	1.68E-04	3.48E-07	NA	0.0071	0.0044	0.0098	0.0014	Symptoms	Pain	+
Q0517	HDL cholesterol	3.71E-04	9.07E-07	NA	-0.0075	-0.011	-0.0045	0.0015	Quantitative Measure	Blood Biochemistry	-
Q0012	Hand grip strength	4.51E-04	1.19E-06	NA	0.057	0.034	0.079	0.012	Quantitative Measure	Body Measurements	+
Q0516	Glycated haemoglobin (HbA1c)	1.12E-03	3.79E-06	NA	0.0072	0.0041	0.01	0.0016	Quantitative Measure	Blood Biochemistry	+
P2035	Diabetes mellitus	1.72E-03	3.24E-07	1.0349	NA	1.0214	1.0486	NA	Endocrine/Metabolic	Endocrine/Metabolic	+
P2035.2	Type 2 diabetes	1.98E-03	1.38E-06	1.034	NA	1.02	1.0481	NA	Endocrine/Metabolic	Endocrine/Metabolic	+
P250	Diabetes mellitus	1.98E-03	1.49E-06	1.028	NA	1.0165	1.0396	NA	Endocrine/Metabolic	Endocrine/Metabolic	+
P250.2	Type 2 diabetes	1.98E-03	1.26E-06	1.0291	NA	1.0172	1.0411	NA	Endocrine/Metabolic	Endocrine/Metabolic	+
Q0110	Systolic blood pressure	2.54E-03	9.57E-06	NA	0.0067	0.0037	0.0097	0.0015	Quantitative Measure	Body Measurements	+
P495	Asthma	3.09E-03	2.91E-06	1.0256	NA	1.0148	1.0365	NA	Respiratory	Respiratory	+
Q0511	Creatinine	5.01E-03	2.17E-05	NA	0.0056	0.003	0.0081	0.0013	Quantitative Measure	Blood Biochemistry	+
Q0528	Triglycerides	8.13E-03	3.98E-05	NA	0.0065	0.0034	0.0096	0.0016	Quantitative Measure	Blood Biochemistry	+

Supplementary Figures

Supplementary Figure 1A. Forest plots for novel sentinel variants.
Filled triangles denote studies contributing to the chronic dry cough GWAS and empty triangles denote studies contributing to the ACEi-induced cough GWAS.

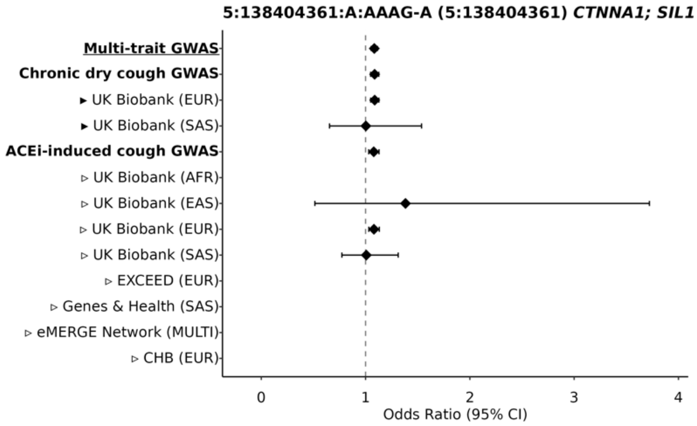

Study	N	OR	95% CI	*p*-value
**Multi-trait GWAS**	134909	1.08	[1.05, 1.11]	2.62e-08
**Chronic dry cough GWAS**	96288	1.09	[1.05, 1.13]	5.99e-06
▸ UK Biobank (EUR)	95399	1.09	[1.05, 1.13]	5.58e-06
▸ UK Biobank (SAS)	889	1.00	[0.65, 1.54]	0.99
**ACEi-induced cough GWAS**	38621	1.08	[1.03, 1.13]	0.00115
▹ UK Biobank (AFR)	0	-	-	-
▹ UK Biobank (EAS)	148	1.38	[0.51, 3.72]	0.521
▹ UK Biobank (EUR)	37372	1.08	[1.03, 1.13]	0.00109
▹ UK Biobank (SAS)	1101	1.01	[0.77, 1.31]	0.958
▹ EXCEED (EUR)	0	-	-	-
▹ Genes & Health (SAS)	0	-	-	-
▹ eMERGE Network (MULTI)	0	-	-	-
▹ CHB (EUR)	0	-	-	-

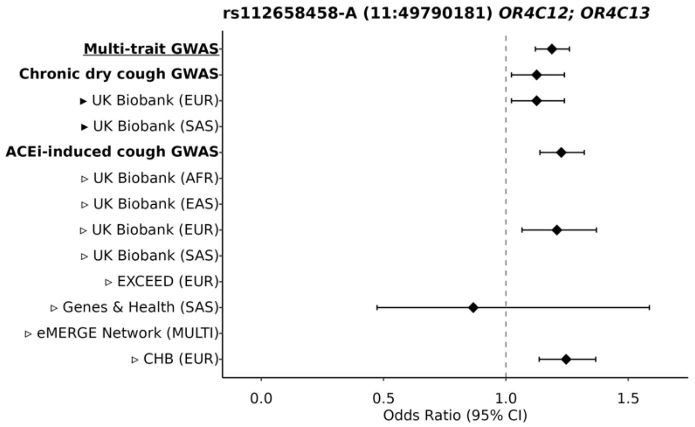

Study	N	OR	95% CI	*p*-value
**Multi-trait GWAS**	169916	1.19	[1.12, 1.26]	8.31e-09
**Chronic dry cough GWAS**	95399	1.13	[1.02, 1.24]	0.0156
▸ UK Biobank (EUR)	95399	1.13	[1.02, 1.24]	0.0156
▸ UK Biobank (SAS)	0	-	-	-
**ACEi-induced cough GWAS**	74517	1.23	[1.14, 1.32]	6.3e-08
▹ UK Biobank (AFR)	0	-	-	-
▹ UK Biobank (EAS)	0	-	-	-
▹ UK Biobank (EUR)	37372	1.21	[1.07, 1.37]	0.00311
▹ UK Biobank (SAS)	0	-	-	-
▹ EXCEED (EUR)	0	-	-	-
▹ Genes & Health (SAS)	7599	0.87	[0.47, 1.59]	0.643
▹ eMERGE Network (MULTI)	0	-	-	-
▹ CHB (EUR)	29546	1.25	[1.14, 1.37]	3.1e-06

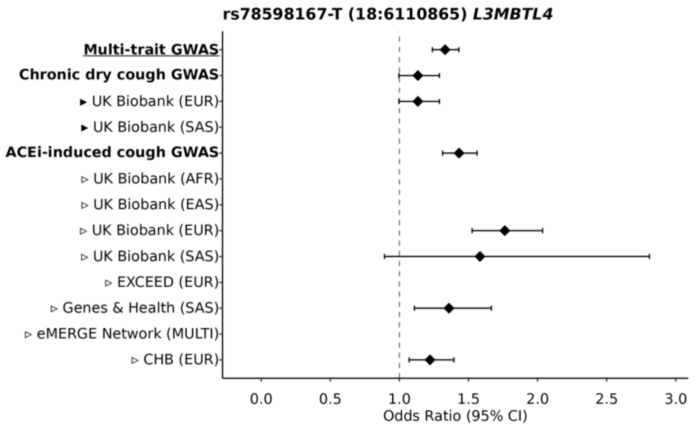

Study	N	OR	95% CI	*p*-value
**Multi-trait GWAS**	171017	1.33	[1.24, 1.43]	8.36e-15
**Chronic dry cough GWAS**	95399	1.13	[1.00, 1.29]	0.0566
▸ UK Biobank (EUR)	95399	1.13	[1.00, 1.29]	0.0566
▸ UK Biobank (SAS)	0	-	-	-
**ACEi-induced cough GWAS**	75618	1.43	[1.31, 1.56]	6.1e-16
▹ UK Biobank (AFR)	0	-	-	-
▹ UK Biobank (EAS)	0	-	-	-
▹ UK Biobank (EUR)	37372	1.76	[1.53, 2.04]	1.31e-14
▹ UK Biobank (SAS)	1101	1.58	[0.89, 2.81]	0.117
▹ EXCEED (EUR)	0	-	-	-
▹ Genes & Health (SAS)	7599	1.36	[1.11, 1.67]	0.00423
▹ eMERGE Network (MULTI)	0	-	-	-
▹ CHB (EUR)	29546	1.22	[1.07, 1.39]	0.003

rs5924943-T (X:150594104) *VMA21*

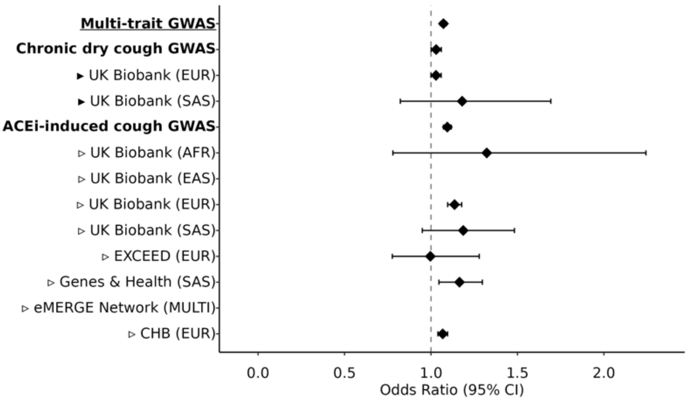

Study	N	OR	95% CI	*p*-value
**Multi-trait GWAS**	173152	1.07	[1.05, 1.09]	3.76e-16
**Chronic dry cough GWAS**	96288	1.03	[1.00, 1.06]	0.044
▸ UK Biobank (EUR)	95399	1.03	[1.00, 1.06]	0.0513
▸ UK Biobank (SAS)	889	1.18	[0.82, 1.69]	0.368
**ACEi-induced cough GWAS**	76864	1.09	[1.07, 1.12]	7.96e-18
▹ UK Biobank (AFR)	408	1.32	[0.78, 2.24]	0.3
▹ UK Biobank (EAS)	0	-	-	-
▹ UK Biobank (EUR)	37372	1.14	[1.10, 1.18]	2.03e-12
▹ UK Biobank (SAS)	1101	1.19	[0.95, 1.48]	0.133
▹ EXCEED (EUR)	838	1.00	[0.78, 1.28]	0.977
▹ Genes & Health (SAS)	7599	1.17	[1.05, 1.30]	0.0049
▹ eMERGE Network (MULTI)	0	-	-	-
▹ CHB (EUR)	29546	1.07	[1.04, 1.10]	9.8e-07

rs141733360-A (4:14727977) *CPEB2*

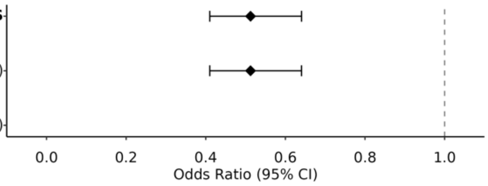

Study	N	OR	95% CI	*p*-value
**Chronic dry cough GWAS**	95399	0.51	[0.41, 0.64]	0.0000000042
▸ UK Biobank (EUR)	95399	0.51	[0.41, 0.64]	0.0000000042
▸ UK Biobank (SAS)	0	-	-	-

rs11172406-A (12:58366606) *ATP23; CYP27B1*

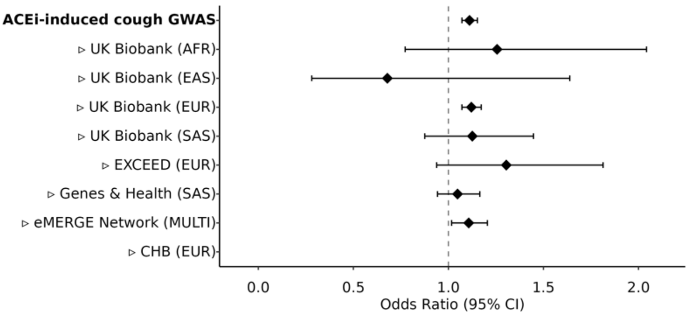

Study	N	OR	95% CI	*p*-value
**ACEi-induced cough GWAS**	54546	1.11	[1.07, 1.15]	1.73e-08
▹ UK Biobank (AFR)	408	1.26	[0.77, 2.04]	0.358
▹ UK Biobank (EAS)	148	0.68	[0.28, 1.64]	0.389
▹ UK Biobank (EUR)	37372	1.12	[1.07, 1.17]	8.45e-07
▹ UK Biobank (SAS)	1101	1.13	[0.88, 1.45]	0.355
▹ EXCEED (EUR)	838	1.30	[0.94, 1.81]	0.114
▹ Genes & Health (SAS)	7599	1.05	[0.94, 1.17]	0.385
▹ eMERGE Network (MULTI)	7080	1.11	[1.02, 1.21]	0.0187
▹ CHB (EUR)	0	-	-	-

rs35336617-T (12:85913015) *ALX1; RASSF9*

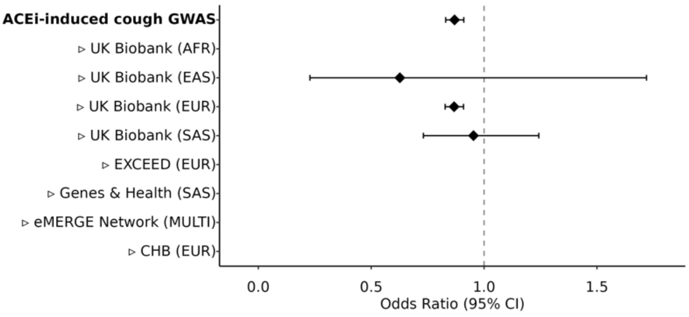

Study	N	OR	95% CI	*p*-value
**ACEi-induced cough GWAS**	38621	0.87	[0.83, 0.91]	2.71e-09
▹ UK Biobank (AFR)	0	-	-	-
▹ UK Biobank (EAS)	148	0.63	[0.23, 1.72]	0.364
▹ UK Biobank (EUR)	37372	0.87	[0.83, 0.91]	2.82e-09
▹ UK Biobank (SAS)	1101	0.95	[0.73, 1.24]	0.723
▹ EXCEED (EUR)	0	-	-	-
▹ Genes & Health (SAS)	0	-	-	-
▹ eMERGE Network (MULTI)	0	-	-	-
▹ CHB (EUR)	0	-	-	-

Supplementary Figure 1B. Forest plots for previously reported sentinel variants.
Filled triangles denote studies contributing to the chronic dry cough GWAS and empty triangles denote studies contributing to the ACEi-induced cough GWAS.

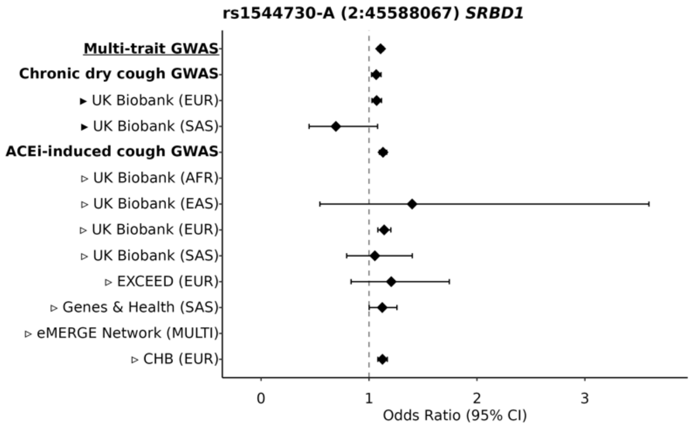

Study	N	OR	95% CI	*p*-value
**Multi-trait GWAS**	172892	1.11	[1.08, 1.13]	1.72e-16
**Chronic dry cough GWAS**	96288	1.07	[1.02, 1.11]	0.00223
▸ UK Biobank (EUR)	95399	1.07	[1.03, 1.12]	0.00127
▸ UK Biobank (SAS)	889	0.69	[0.45, 1.08]	0.105
**ACEi-induced cough GWAS**	76604	1.13	[1.10, 1.16]	1.6e-15
▹ UK Biobank (AFR)	0	-	-	-
▹ UK Biobank (EAS)	148	1.40	[0.54, 3.59]	0.485
▹ UK Biobank (EUR)	37372	1.14	[1.08, 1.20]	1.1e-06
▹ UK Biobank (SAS)	1101	1.05	[0.79, 1.40]	0.717
▹ EXCEED (EUR)	838	1.21	[0.83, 1.74]	0.32
▹ Genes & Health (SAS)	7599	1.12	[1.00, 1.26]	0.047
▹ eMERGE Network (MULTI)	0	-	-	-
▹ CHB (EUR)	29546	1.12	[1.08, 1.17]	3.4e-09

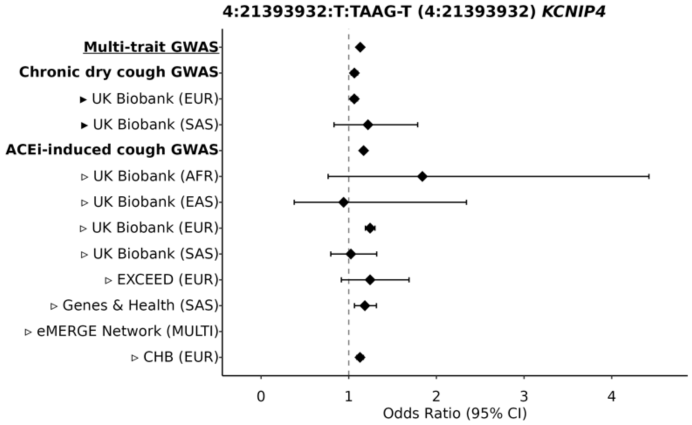

Study	N	OR	95% CI	*p*-value
**Multi-trait GWAS**	173300	1.13	[1.11, 1.15]	4.97e-32
**Chronic dry cough GWAS**	96288	1.06	[1.03, 1.10]	0.000418
▸ UK Biobank (EUR)	95399	1.06	[1.03, 1.10]	0.00056
▸ UK Biobank (SAS)	889	1.22	[0.83, 1.79]	0.309
**ACEi-induced cough GWAS**	77012	1.17	[1.14, 1.20]	1.96e-33
▹ UK Biobank (AFR)	408	1.84	[0.77, 4.42]	0.173
▹ UK Biobank (EAS)	148	0.94	[0.38, 2.34]	0.896
▹ UK Biobank (EUR)	37372	1.24	[1.19, 1.30]	1.48e-22
▹ UK Biobank (SAS)	1101	1.02	[0.80, 1.32]	0.855
▹ EXCEED (EUR)	838	1.24	[0.92, 1.69]	0.163
▹ Genes & Health (SAS)	7599	1.18	[1.07, 1.32]	0.00176
▹ eMERGE Network (MULTI)	0	-	-	-
▹ CHB (EUR)	29546	1.13	[1.09, 1.17]	1.2e-12

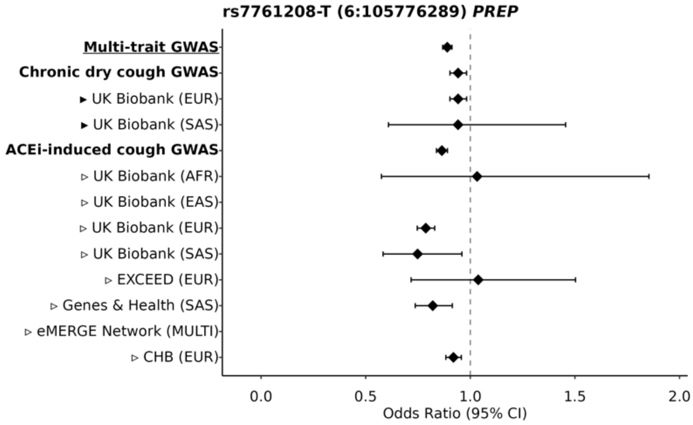

Study	N	OR	95% CI	*p*-value
**Multi-trait GWAS**	173152	0.89	[0.87, 0.91]	1.72e-20
**Chronic dry cough GWAS**	96288	0.94	[0.90, 0.98]	0.00519
▸ UK Biobank (EUR)	95399	0.94	[0.90, 0.98]	0.00541
▸ UK Biobank (SAS)	889	0.94	[0.61, 1.46]	0.786
**ACEi-induced cough GWAS**	76864	0.86	[0.84, 0.89]	4.36e-21
▹ UK Biobank (AFR)	408	1.03	[0.58, 1.85]	0.915
▹ UK Biobank (EAS)	0	-	-	-
▹ UK Biobank (EUR)	37372	0.79	[0.75, 0.83]	8.78e-19
▹ UK Biobank (SAS)	1101	0.75	[0.58, 0.96]	0.0225
▹ EXCEED (EUR)	838	1.04	[0.72, 1.50]	0.844
▹ Genes & Health (SAS)	7599	0.82	[0.74, 0.91]	0.000362
▹ eMERGE Network (MULTI)	0	-	-	-
▹ CHB (EUR)	29546	0.92	[0.88, 0.96]	4.2e-05

rs7848821-A (9:128251930) *MAPKAP1*

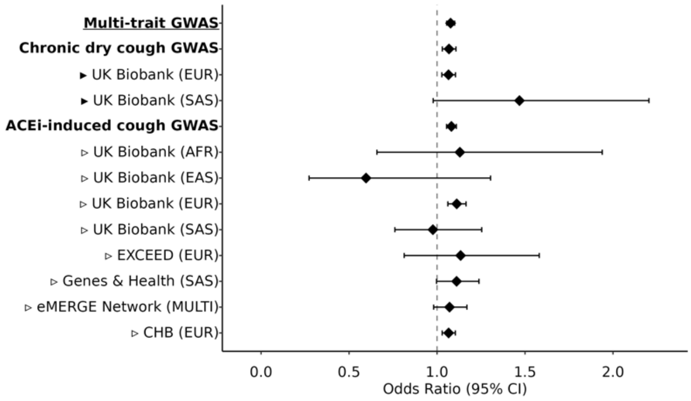

	N	OR	95% CI	*p*-value
**Multi-trait GWAS**	180380	1.08	[1.05, 1.10]	4.02e-12
**Chronic dry cough GWAS**	96288	1.07	[1.03, 1.11]	0.000381
▸ UK Biobank (EUR)	95399	1.06	[1.03, 1.10]	0.000671
▸ UK Biobank (SAS)	889	1.47	[0.98, 2.20]	0.0639
**ACEi-induced cough GWAS**	84092	1.08	[1.05, 1.11]	2.1e-09
▹ UK Biobank (AFR)	408	1.13	[0.66, 1.94]	0.658
▹ UK Biobank (EAS)	148	0.60	[0.27, 1.30]	0.195
▹ UK Biobank (EUR)	37372	1.11	[1.06, 1.16]	7.57e-06
▹ UK Biobank (SAS)	1101	0.98	[0.76, 1.25]	0.852
▹ EXCEED (EUR)	838	1.13	[0.81, 1.58]	0.458
▹ Genes & Health (SAS)	7599	1.11	[1.00, 1.24]	0.0566
▹ eMERGE Network (MULTI)	7080	1.07	[0.98, 1.17]	0.129
▹ CHB (EUR)	29546	1.07	[1.03, 1.10]	0.00037

rs343240-T (18:6308062) *L3MBTL4*

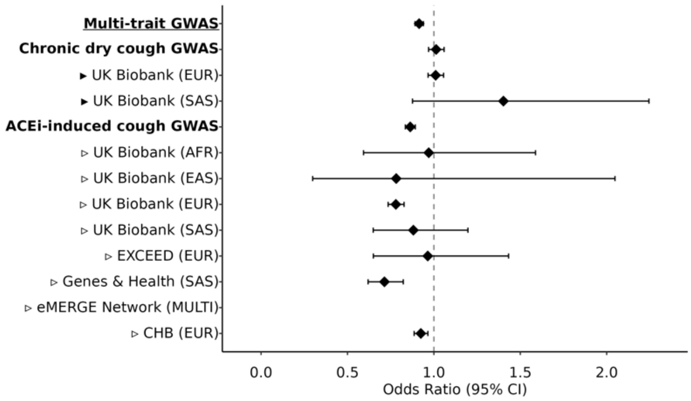

	N	OR	95% CI	*p*-value
**Multi-trait GWAS**	173300	0.92	[0.89, 0.94]	4.9e-11
**Chronic dry cough GWAS**	96288	1.01	[0.97, 1.06]	0.564
▸ UK Biobank (EUR)	95399	1.01	[0.97, 1.06]	0.655
▸ UK Biobank (SAS)	889	1.40	[0.88, 2.24]	0.159
**ACEi-induced cough GWAS**	77012	0.86	[0.83, 0.89]	5.18e-18
▹ UK Biobank (AFR)	408	0.97	[0.59, 1.59]	0.904
▹ UK Biobank (EAS)	148	0.78	[0.30, 2.05]	0.617
▹ UK Biobank (EUR)	37372	0.78	[0.74, 0.83]	1.61e-16
▹ UK Biobank (SAS)	1101	0.88	[0.65, 1.20]	0.418
▹ EXCEED (EUR)	838	0.96	[0.65, 1.43]	0.858
▹ Genes & Health (SAS)	7599	0.71	[0.62, 0.82]	2.03e-06
▹ eMERGE Network (MULTI)	0	-	-	-
▹ CHB (EUR)	29546	0.93	[0.89, 0.97]	0.00035

rs6062847-T (20:61322018) *NTSR1; SLCO4A1*

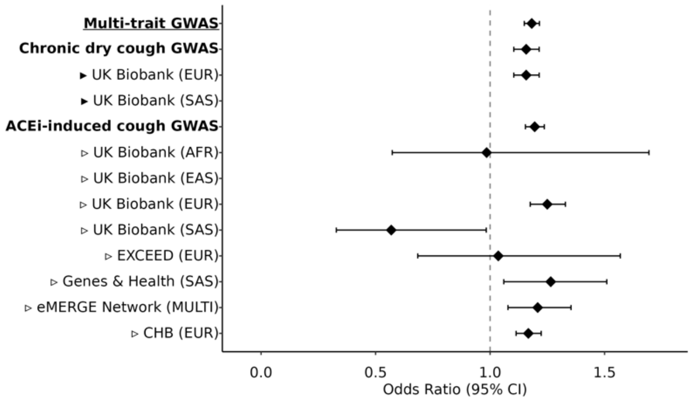

	N	OR	95% CI	*p*-value
**Multi-trait GWAS**	179343	1.18	[1.15, 1.22]	1.1e-31
**Chronic dry cough GWAS**	95399	1.16	[1.10, 1.21]	2.07e-09
▸ UK Biobank (EUR)	95399	1.16	[1.10, 1.21]	2.07e-09
▸ UK Biobank (SAS)	0	-	-	-
**ACEi-induced cough GWAS**	83944	1.20	[1.15, 1.24]	4.31e-24
▹ UK Biobank (AFR)	408	0.98	[0.57, 1.69]	0.956
▹ UK Biobank (EAS)	0	-	-	-
▹ UK Biobank (EUR)	37372	1.25	[1.18, 1.33]	1.07e-12
▹ UK Biobank (SAS)	1101	0.57	[0.33, 0.98]	0.0434
▹ EXCEED (EUR)	838	1.04	[0.68, 1.57]	0.868
▹ Genes & Health (SAS)	7599	1.26	[1.06, 1.51]	0.0108
▹ eMERGE Network (MULTI)	7080	1.21	[1.08, 1.35]	0.00113
▹ CHB (EUR)	29546	1.17	[1.11, 1.22]	1e-10

rs7518061-T (1:111087944) *KCNA10; RBM15*

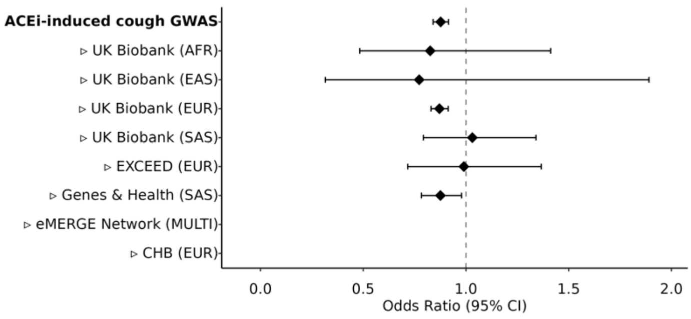

	N	OR	95% CI	*p*-value
**ACEi-induced cough GWAS**	47466	0.88	[0.84, 0.92]	1.85e-09
▹ UK Biobank (AFR)	408	0.83	[0.48, 1.41]	0.485
▹ UK Biobank (EAS)	148	0.77	[0.32, 1.89]	0.572
▹ UK Biobank (EUR)	37372	0.87	[0.83, 0.91]	1.54e-08
▹ UK Biobank (SAS)	1101	1.03	[0.79, 1.34]	0.82
▹ EXCEED (EUR)	838	0.99	[0.72, 1.37]	0.95
▹ Genes & Health (SAS)	7599	0.88	[0.78, 0.98]	0.0193
▹ eMERGE Network (MULTI)	0	-	-	-
▹ CHB (EUR)	0	-	-	-

Supplementary Figure 2. Putative causal genes identified by variant-to-gene mapping.
Filled circles indicate genes identified by specific analysis. Protein expression and rare variant columns have been omitted as these did not lead to the mapping of any genes. Novel genes are in bold.

Variant-to-gene mapping

Gene	Nearest gene	Pathogenicity predictions	Polygenic priority score	Gene expression
KCNIP4	●	●	●	
MAPKAP1	●	●	●	
PREP	●		●	●
ALX1	●		●	
ATP23	●			●
CPEB2	●		●	
L3MBTL4	●		●	
OR4C13	●	●		
SIL1	●			●
SRBD1	●		●	
CTNNA1			●	
CYP27B1			●	
KCNA10	●			
NTSR1	●			
OR4C12			●	
RASSF9				●
RBM15			●	
SLCO4A1			●	
VMA21	●			

Supplementary Figure 3. Sensitivity analysis excluding cases who coughed for less than one-year from the chronic dry cough trait.

Sentinel variant effect sizes (beta) in the multi-trait primary analysis and sensitivity analysis excluding short-term chronic dry cough cases are plotted. Standard errors are reported in orange.

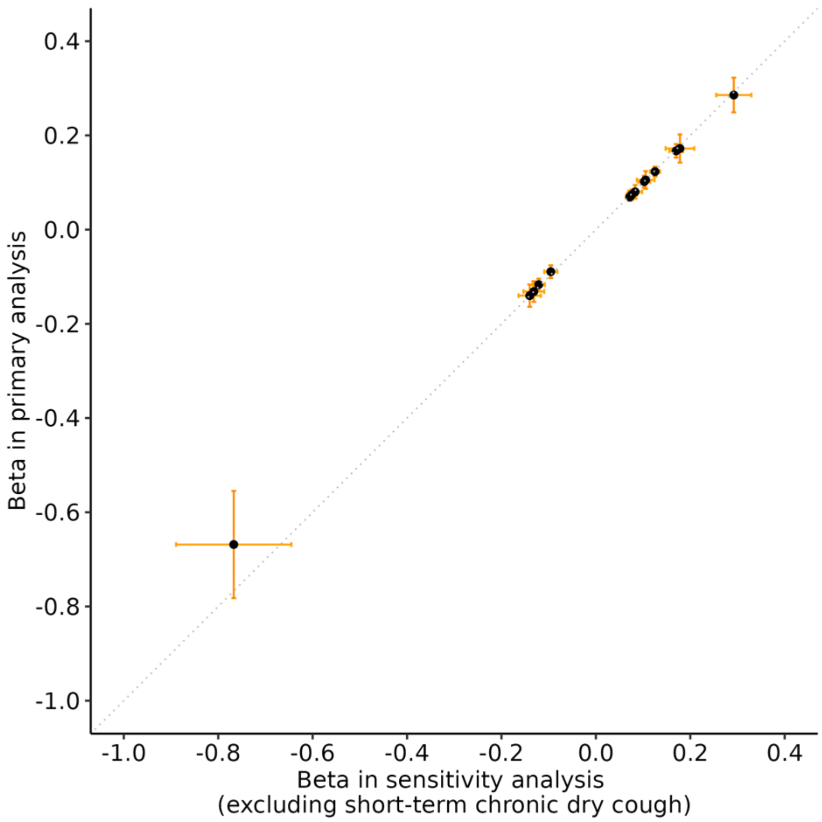

Supplementary Figure 4. Sensitivity analysis in UK Biobank excluding cases with a clinical cough code within 12 months after switching to an ARB from the ACEi-induced cough trait.
Sentinel variant effect sizes (beta) in the multi-trait primary analysis and UK Biobank-specific sensitivity analysis excluding cases with a clinical report of coughing after switching.

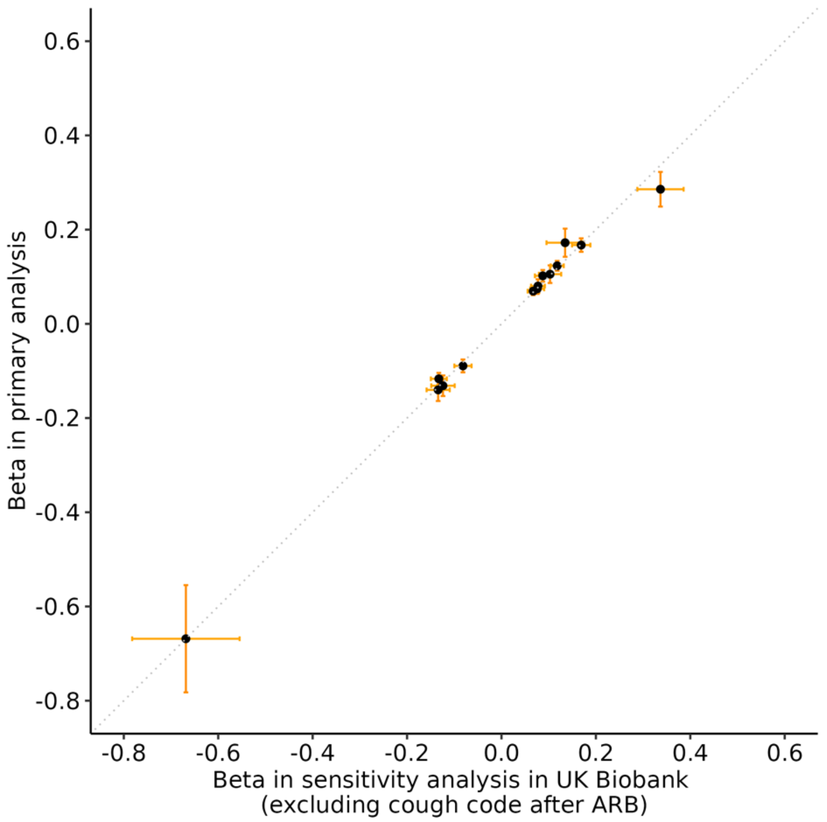

Supplementary Figure 5. Sensitivity analysis in UK Biobank excluding asthma cases from the chronic dry cough and ACEi-induced cough traits.

Sentinel variant effect sizes (beta) in the multi-trait primary analysis and UK Biobank-specific sensitivity analysis excluding asthma cases are plotted. Standard errors are reported in orange.

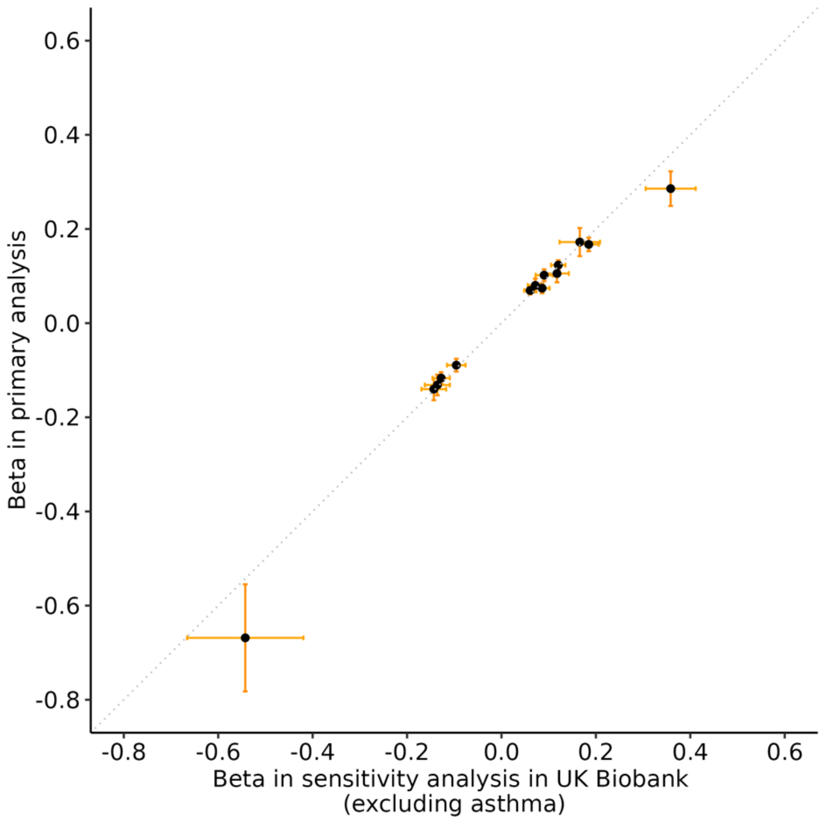

Supplementary Figure 6. Enrichment of heritability of chronic dry cough and ACEi-induced cough across tissue types using expressed genes (A) and chromatin regions (B-F).
Grey vertical dotted lines denote nominal significance and Bonferroni corrected significance thresholds.

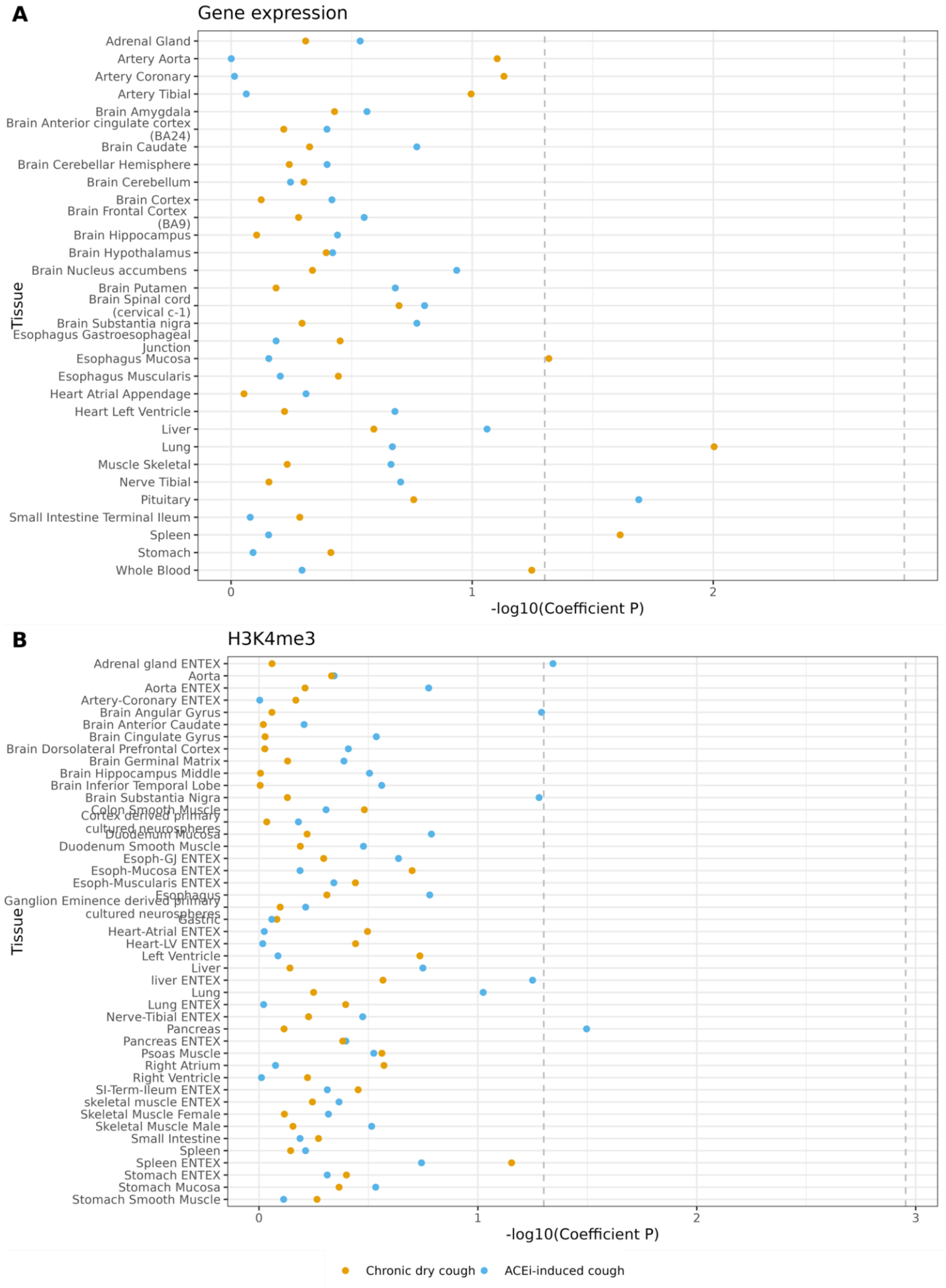

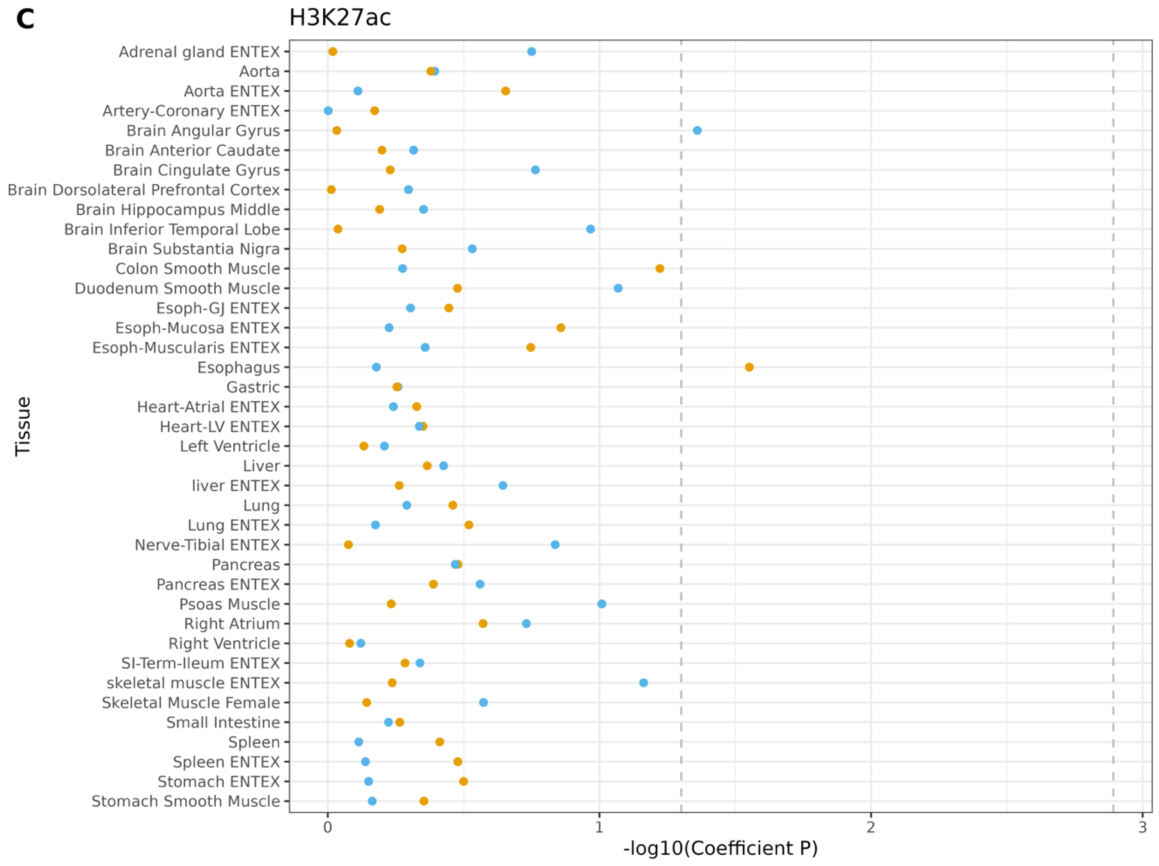
C
H3K27ac
Tissue
Adrenal gland ENTEX
Aorta
Aorta ENTEX
Artery-Coronary ENTEX
Brain Angular Gyrus
Brain Anterior Caudate
Brain Cingulate Gyrus
Brain Dorsolateral Prefrontal Cortex
Brain Hippocampus Middle
Brain Inferior Temporal Lobe
Brain Substantia Nigra
Colon Smooth Muscle
Duodenum Smooth Muscle
Esoph-GJ ENTEX
Esoph-Mucosa ENTEX
Esoph-Muscularis ENTEX
Esophagus
Gastric
Heart-Atrial ENTEX
Heart-LV ENTEX
Left Ventricle
Liver
liver ENTEX
Lung
Lung ENTEX
Nerve-Tibial ENTEX
Pancreas
Pancreas ENTEX
Psoas Muscle
Right Atrium
Right Ventricle
SI-Term-Ileum ENTEX
skeletal muscle ENTEX
Skeletal Muscle Female
Small Intestine
Spleen
Spleen ENTEX
Stomach ENTEX
Stomach Smooth Muscle
0
1
2
3
-log10(Coefficient P)

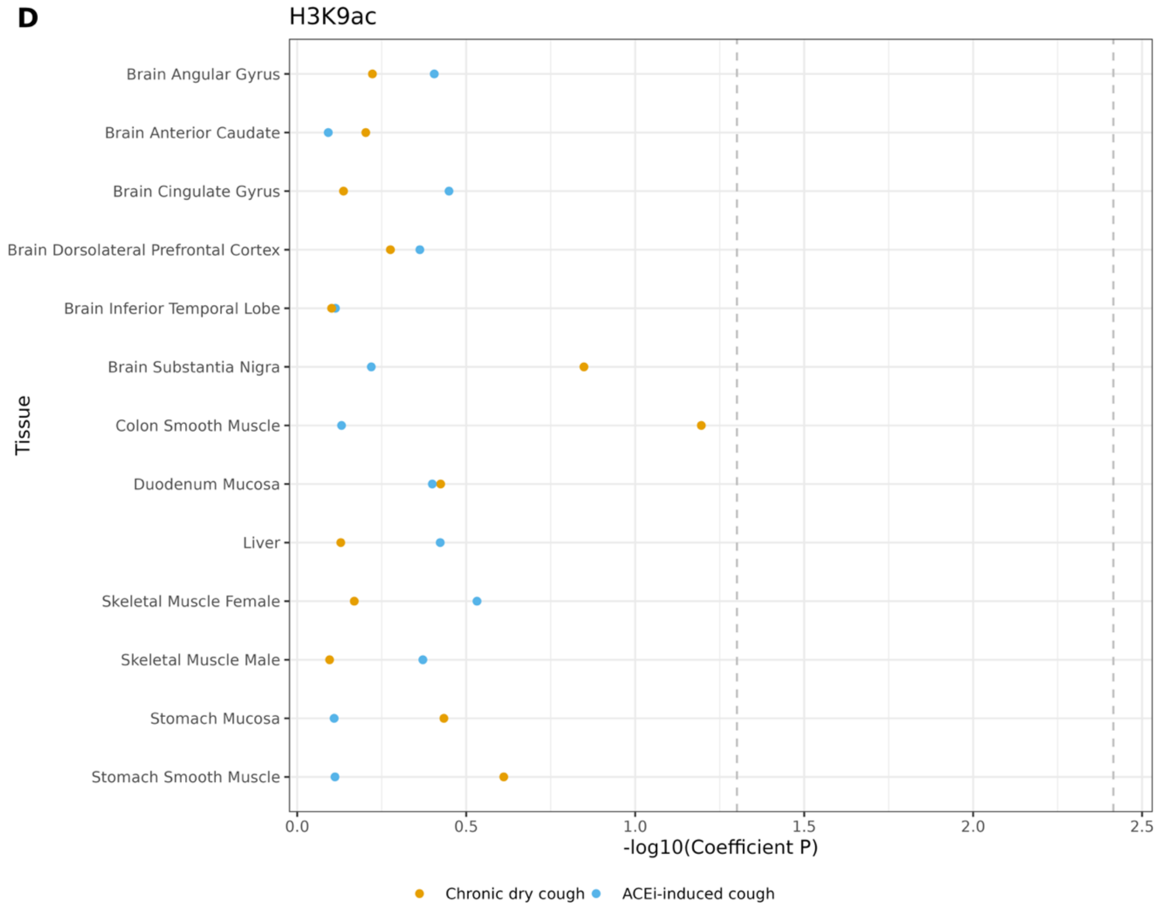
D
H3K9ac
Tissue
Brain Angular Gyrus
Brain Anterior Caudate
Brain Cingulate Gyrus
Brain Dorsolateral Prefrontal Cortex
Brain Inferior Temporal Lobe
Brain Substantia Nigra
Colon Smooth Muscle
Duodenum Mucosa
Liver
Skeletal Muscle Female
Skeletal Muscle Male
Stomach Mucosa
Stomach Smooth Muscle
0.0
0.5
1.0
1.5
2.0
2.5
-log10(Coefficient P)
Chronic dry cough
ACEi-induced cough

E

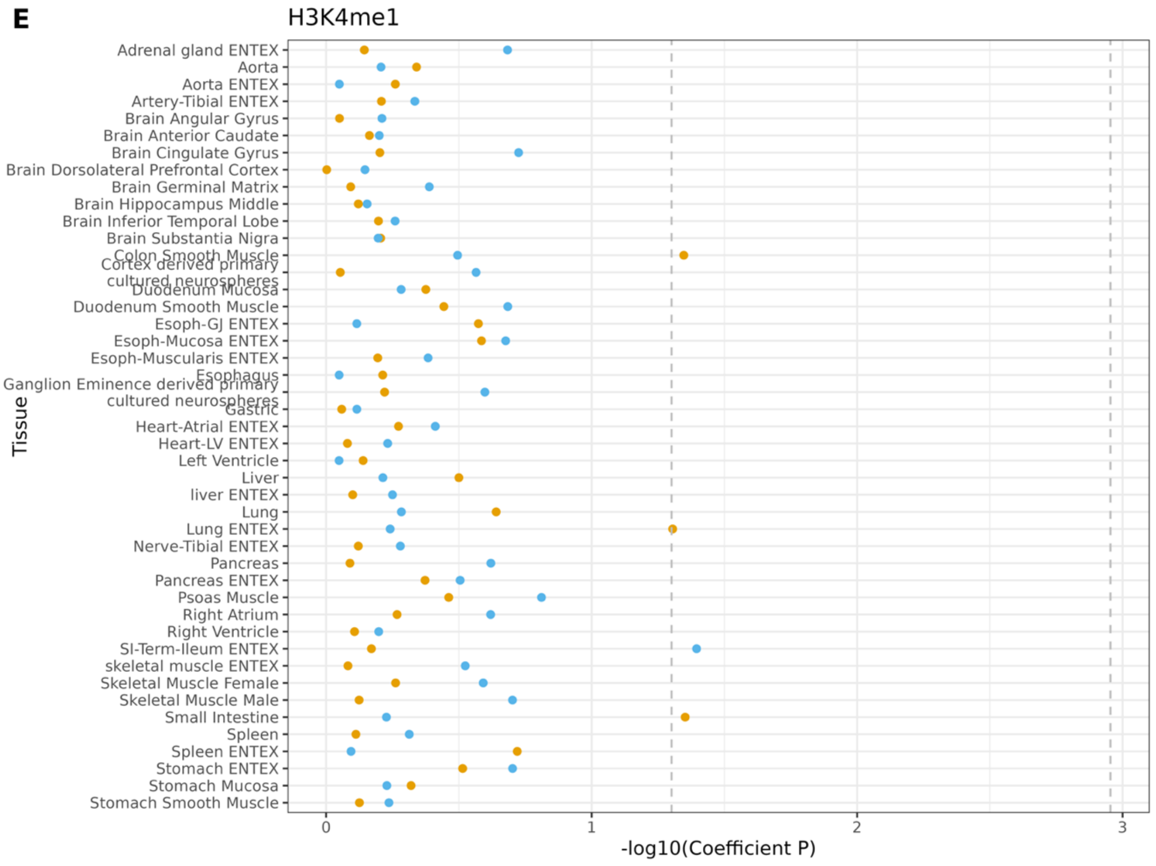
H3K4me1
Tissue
-log10(Coefficient P)
0
1
2
3
Adrenal gland ENTEX
Aorta
Aorta ENTEX
Artery-Tibial ENTEX
Brain Angular Gyrus
Brain Anterior Caudate
Brain Cingulate Gyrus
Brain Dorsolateral Prefrontal Cortex
Brain Germinal Matrix
Brain Hippocampus Middle
Brain Inferior Temporal Lobe
Brain Substantia Nigra
Colon Smooth Muscle
Cortex derived primary cultured neurospheres
Duodenum Mucosa
Duodenum Smooth Muscle
Esoph-GJ ENTEX
Esoph-Mucosa ENTEX
Esoph-Muscularis ENTEX
Esophagus
Ganglion Eminence derived primary cultured neurospheres
Gastric
Heart-Atrial ENTEX
Heart-LV ENTEX
Left Ventricle
Liver
liver ENTEX
Lung
Lung ENTEX
Nerve-Tibial ENTEX
Pancreas
Pancreas ENTEX
Psoas Muscle
Right Atrium
Right Ventricle
SI-Term-Ileum ENTEX
skeletal muscle ENTEX
Skeletal Muscle Female
Skeletal Muscle Male
Small Intestine
Spleen
Spleen ENTEX
Stomach ENTEX
Stomach Mucosa
Stomach Smooth Muscle

F

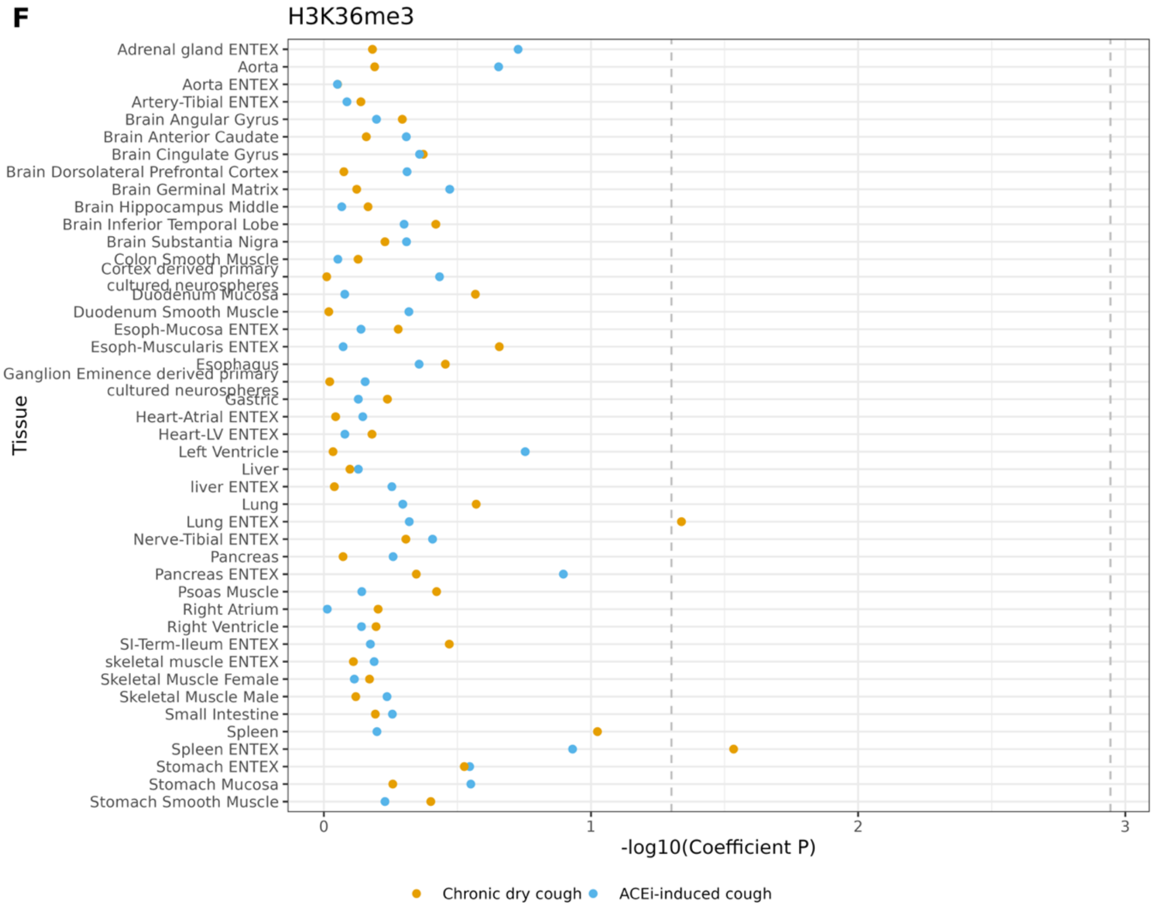
H3K36me3
Tissue
-log10(Coefficient P)
0
1
2
3
Adrenal gland ENTEX
Aorta
Aorta ENTEX
Artery-Tibial ENTEX
Brain Angular Gyrus
Brain Anterior Caudate
Brain Cingulate Gyrus
Brain Dorsolateral Prefrontal Cortex
Brain Germinal Matrix
Brain Hippocampus Middle
Brain Inferior Temporal Lobe
Brain Substantia Nigra
Colon Smooth Muscle
Cortex derived primary cultured neurospheres
Duodenum Mucosa
Duodenum Smooth Muscle
Esoph-Mucosa ENTEX
Esoph-Muscularis ENTEX
Esophagus
Ganglion Eminence derived primary cultured neurospheres
Gastric
Heart-Atrial ENTEX
Heart-LV ENTEX
Left Ventricle
Liver
liver ENTEX
Lung
Lung ENTEX
Nerve-Tibial ENTEX
Pancreas
Pancreas ENTEX
Psoas Muscle
Right Atrium
Right Ventricle
SI-Term-Ileum ENTEX
skeletal muscle ENTEX
Skeletal Muscle Female
Skeletal Muscle Male
Small Intestine
Spleen
Spleen ENTEX
Stomach ENTEX
Stomach Mucosa
Stomach Smooth Muscle
Chronic dry cough
ACEi-induced cough

Supplementary Figure 7. Genetic correlations of chronic dry cough and ACEi-induced cough with clinical conditions associated with genetic predisposition to ACEi-induced cough.

Filled circles represent point estimate for genetic correlation and error bars indicate 95% confidence intervals. Asterisk above point estimate denotes *p*-value <0.05 for specific trait combination.

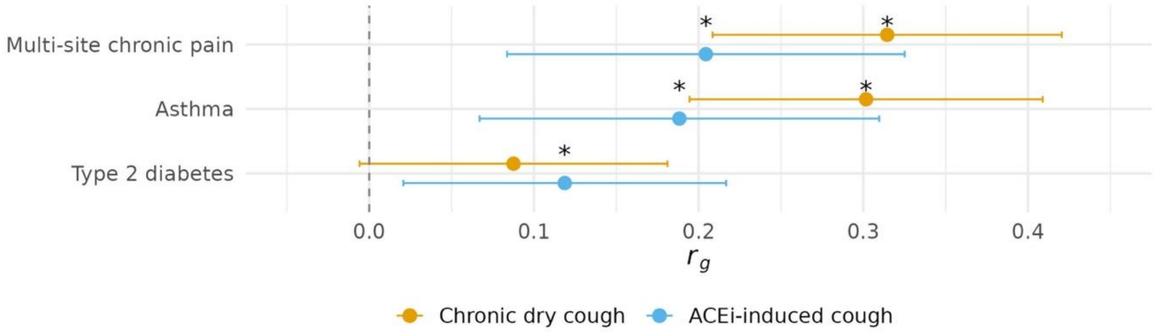

References

1. Bycroft C, Freeman C, Petkova D, et al. The UK Biobank resource with deep phenotyping and genomic data. *Nature*. Oct 2018;562(7726):203-209. doi:10.1038/s41586-018-0579-z

2. Shrine N, Guyatt AL, Erzurumluoglu AM, et al. New genetic signals for lung function highlight pathways and chronic obstructive pulmonary disease associations across multiple ancestries. *Nat Genet*. Mar 2019;51(3):481-493. doi:10.1038/s41588-018-0321-7

3. John C, Reeve NF, Free RC, et al. Cohort profile: Extended Cohort for E-health, Environment and DNA (EXCEED). *Int J Epidemiol*. Oct 1 2019;48(5):1734. doi:10.1093/ije/dyz175

4. DReichLab. EIG. https://github.com/DReichLab/EIG

5. 1000 Genomes Project Consortium. A global reference for human genetic variation. *Nature*. Oct 1 2015;526(7571):68-74. doi:10.1038/nature15393

6. Finer S, Martin HC, Khan A, et al. Cohort Profile: East London Genes & Health (ELGH), a community-based population genomics and health study in British Bangladeshi and British Pakistani people. *Int J Epidemiol*. Feb 1 2020;49(1):20-21i. doi:10.1093/ije/dyz174

7. Sorensen E, Christiansen L, Wilkowski B, et al. Data Resource Profile: The Copenhagen Hospital Biobank (CHB). *Int J Epidemiol*. Jul 9 2021;50(3):719-720e. doi:10.1093/ije/dyaa157

8. Ghouse J, Tragante V, Muhammad A, et al. Polygenic risk score for ACE-inhibitor-associated cough based on the discovery of new genetic loci. *Eur Heart J*. Dec 1 2022;43(45):4707-4718. doi:10.1093/eurheartj/ehac322

9. Mosley JD, Shaffer CM, Van Driest SL, et al. A genome-wide association study identifies variants in KCNIP4 associated with ACE inhibitor-induced cough. *Pharmacogenomics J*. Jun 2016;16(3):231-7. doi:10.1038/tpj.2015.51

10. Gottesman O, Kuivaniemi H, Tromp G, et al. The Electronic Medical Records and Genomics (eMERGE) Network: past, present, and future. *Genet Med*. Oct 2013;15(10):761-71. doi:10.1038/gim.2013.72

11. All of Us Research Program Investigators, Denny JC, Rutter JL, et al. The "All of Us" Research Program. *N Engl J Med*. Aug 15 2019;381(7):668-676. doi:10.1056/NEJMsr1809937

12. All of Us Research Program Genomics Investigators. Genomic data in the All of Us Research Program. *Nature*. Mar 2024;627(8003):340-346. doi:10.1038/s41586-023-06957-x

13. Bergstrom A, McCarthy SA, Hui R, et al. Insights into human genetic variation and population history from 929 diverse genomes. *Science*. Mar 20 2020;367(6484)doi:10.1126/science.aay5012

14. Packer RJ, Williams AT, Hennah W, et al. DeepPheWAS: an R package for phenotype generation and association analysis for phenome-wide association studies. *Bioinformatics*. Apr 3 2023;39(4)doi:10.1093/bioinformatics/btad073

15. Center for Statistical Genetics (University of Michigan). MaCH FAQ. Accessed 1st April 2025, https://genome.sph.umich.edu/wiki/MaCH_FAQ

16. Roshyara NR, Kirsten H, Horn K, Ahnert P, Scholz M. Impact of pre-imputation SNP-filtering on genotype imputation results. *BMC Genet*. Aug 12 2014;15:88. doi:10.1186/s12863-014-0088-5

17. Karolchik D, Hinrichs AS, Kent WJ. The UCSC Genome Browser. *Curr Protoc Bioinformatics*. Dec 2012;Chapter 1:1 4 1-1 4 33. doi:10.1002/0471250953.bi0104s40

18. Willer CJ, Li Y, Abecasis GR. METAL: fast and efficient meta-analysis of genomewide association scans. *Bioinformatics*. Sep 1 2010;26(17):2190-1. doi:10.1093/bioinformatics/btq340

19. Yang J, Ferreira T, Morris AP, et al. Conditional and joint multiple-SNP analysis of GWAS summary statistics identifies additional variants influencing complex traits. *Nat Genet*. Mar 18 2012;44(4):369-75, S1-3. doi:10.1038/ng.2213

20. Yang J, Lee SH, Goddard ME, Visscher PM. GCTA: a tool for genome-wide complex trait analysis. *Am J Hum Genet*. Jan 7 2011;88(1):76-82. doi:10.1016/j.ajhg.2010.11.011

21. Wakefield J. A Bayesian measure of the probability of false discovery in genetic epidemiology studies. *Am J Hum Genet*. Aug 2007;81(2):208-27. doi:10.1086/519024

22. Ge T, Chen CY, Ni Y, Feng YA, Smoller JW. Polygenic prediction via Bayesian regression and continuous shrinkage priors. *Nat Commun*. Apr 16 2019;10(1):1776. doi:10.1038/s41467-019-09718-5

23. Wang K, Li M, Hakonarson H. ANNOVAR: functional annotation of genetic variants from high-throughput sequencing data. *Nucleic Acids Res*. Sep 2010;38(16):e164. doi:10.1093/nar/gkq603

24. McLaren W, Gil L, Hunt SE, et al. The Ensembl Variant Effect Predictor. *Genome Biol*. Jun 6 2016;17(1):122. doi:10.1186/s13059-016-0974-4

25. Weeks EM, Ulirsch JC, Cheng NY, et al. Leveraging polygenic enrichments of gene features to predict genes underlying complex traits and diseases. *Nat Genet*. Jul 13 2023;55(8):1267-1276. doi:10.1038/s41588-023-01443-6

26. Mbatchou J, Barnard L, Backman J, et al. Computationally efficient whole-genome regression for quantitative and binary traits. *Nat Genet*. Jul 2021;53(7):1097-1103. doi:10.1038/s41588-021-00870-7

27. Chang CC, Chow CC, Tellier LC, Vattikuti S, Purcell SM, Lee JJ. Second-generation PLINK: rising to the challenge of larger and richer datasets. *Gigascience*. 2015;4:7. doi:10.1186/s13742-015-0047-8

28. Vosa U, Claringbould A, Westra HJ, et al. Large-scale cis- and trans-eQTL analyses identify thousands of genetic loci and polygenic scores that regulate blood gene expression. *Nat Genet*. Sep 2021;53(9):1300-1310. doi:10.1038/s41588-021-00913-z

29. GTEx Consortium. The GTEx Consortium atlas of genetic regulatory effects across human tissues. *Science*. Sep 11 2020;369(6509):1318-1330. doi:10.1126/science.aaz1776

30. Wallace C. A more accurate method for colocalisation analysis allowing for multiple causal variants. *PLoS Genet*. Sep 2021;17(9):e1009440. doi:10.1371/journal.pgen.1009440

31. Ferkingstad E, Sulem P, Atlason BA, et al. Large-scale integration of the plasma proteome with genetics and disease. *Nat Genet*. Dec 2021;53(12):1712-1721. doi:10.1038/s41588-021-00978-w

32. Folkersen L, Gustafsson S, Wang Q, et al. Genomic and drug target evaluation of 90 cardiovascular proteins in 30,931 individuals. *Nat Metab*. Oct 2020;2(10):1135-1148. doi:10.1038/s42255-020-00287-2

33. Sun BB, Chiou J, Traylor M, et al. Plasma proteomic associations with genetics and health in the UK Biobank. *Nature*. Oct 2023;622(7982):329-338. doi:10.1038/s41586-023-06592-6

34. Bulik-Sullivan B, Finucane HK, Anttila V, et al. An atlas of genetic correlations across human diseases and traits. *Nat Genet*. Nov 2015;47(11):1236-41. doi:10.1038/ng.3406

35. Bulik-Sullivan BK, Loh PR, Finucane HK, et al. LD Score regression distinguishes confounding from polygenicity in genome-wide association studies. *Nat Genet*. Mar 2015;47(3):291-5. doi:10.1038/ng.3211

36. Tsuo K, Zhou W, Wang Y, et al. Multi-ancestry meta-analysis of asthma identifies novel associations and highlights the value of increased power and diversity. *Cell Genom*. Dec 14 2022;2(12):100212. doi:10.1016/j.xgen.2022.100212

37. Zhou W, Kanai M, Wu KH, et al. Global Biobank Meta-analysis Initiative: Powering genetic discovery across human disease. *Cell Genom*. Oct 12 2022;2(10):100192. doi:10.1016/j.xgen.2022.100192

38. Finucane HK, Bulik-Sullivan B, Gusev A, et al. Partitioning heritability by functional annotation using genome-wide association summary statistics. *Nat Genet*. Nov 2015;47(11):1228-35. doi:10.1038/ng.3404

39. GTEx Consortium. Human genomics. The Genotype-Tissue Expression (GTEx) pilot analysis: multitissue gene regulation in humans. *Science*. May 8 2015;348(6235):648-60. doi:10.1126/science.1262110

40. Roadmap Epigenomics Consortium, Kundaje A, Meuleman W, et al. Integrative analysis of 111 reference human epigenomes. *Nature*. Feb 19 2015;518(7539):317-30. doi:10.1038/nature14248

41. Encode Project Consortium. An integrated encyclopedia of DNA elements in the human genome. *Nature*. Sep 6 2012;489(7414):57-74. doi:10.1038/nature11247

42. Gazal S, Finucane HK, Furlotte NA, et al. Linkage disequilibrium-dependent architecture of human complex traits shows action of negative selection. *Nat Genet*. Oct 2017;49(10):1421-1427. doi:10.1038/ng.3954

43. Johnston KJA, Adams MJ, Nicholl BI, et al. Genome-wide association study of multisite chronic pain in UK Biobank. *PLoS Genet*. Jun 2019;15(6):e1008164. doi:10.1371/journal.pgen.1008164

44. Mahajan A, Spracklen CN, Zhang W, et al. Multi-ancestry genetic study of type 2 diabetes highlights the power of diverse populations for discovery and translation. *Nat Genet*. May 2022;54(5):560-572. doi:10.1038/s41588-022-01058-3

45. Olafsdottir TA, Theodors F, Bjarnadottir K, et al. Eighty-eight variants highlight the role of T cell regulation and airway remodeling in asthma pathogenesis. *Nat Commun*. Jan 20 2020;11(1):393. doi:10.1038/s41467-019-14144-8

46. Ferreira MAR, Mathur R, Vonk JM, et al. Genetic Architectures of Childhood- and Adult-Onset Asthma Are Partly Distinct. *Am J Hum Genet*. Apr 4 2019;104(4):665-684. doi:10.1016/j.ajhg.2019.02.022

47. Ahluwalia TS, Eliasen AU, Sevelsted A, et al. FUT2-ABO epistasis increases the risk of early childhood asthma and Streptococcus pneumoniae respiratory illnesses. *Nat Commun*. Dec 16 2020;11(1):6398. doi:10.1038/s41467-020-19814-6

48. Edris A, Voorhies K, Lutz SM, et al. Asthma exacerbations and eosinophilia in the UK Biobank: a genome-wide association study. *ERJ Open Res*. Jan 2024;10(1)doi:10.1183/23120541.00566-2023

49. Sakaue S, Kanai M, Tanigawa Y, et al. A cross-population atlas of genetic associations for 220 human phenotypes. *Nat Genet*. Oct 2021;53(10):1415-1424. doi:10.1038/s41588-021-00931-x

50. Jiang L, Zheng Z, Fang H, Yang J. A generalized linear mixed model association tool for biobank-scale data. *Nat Genet*. Nov 2021;53(11):1616-1621. doi:10.1038/s41588-021-00954-4

51. Backman JD, Li AH, Marcketta A, et al. Exome sequencing and analysis of 454,787 UK Biobank participants. *Nature*. Nov 2021;599(7886):628-634. doi:10.1038/s41586-021-04103-z

52. Kurki MI, Karjalainen J, Palta P, et al. FinnGen provides genetic insights from a well-phenotyped isolated population. *Nature*. Jan 2023;613(7944):508-518. doi:10.1038/s41586-022-05473-8

53. Packer RJ, Shrine N, Hall R, et al. Genome-wide association study of chronic sputum production implicates loci involved in mucus production and infection. *Eur Respir J*. Jun 2023;61(6)doi:10.1183/13993003.01667-2022

54. Partanen JJ, Happola P, Zhou W, et al. Leveraging global multi-ancestry meta-analysis in the study of idiopathic pulmonary fibrosis genetics. *Cell Genom*. Oct 12 2022;2(10):100181. doi:10.1016/j.xgen.2022.100181

55. Allen RJ, Stockwell A, Oldham JM, et al. Genome-wide association study across five cohorts identifies five novel loci associated with idiopathic pulmonary fibrosis. *Thorax*. Aug 2022;77(8):829-833. doi:10.1136/thoraxjnl-2021-218577

56. Allen RJ, Oldham JM, Jenkins DA, et al. Longitudinal lung function and gas transfer in individuals with idiopathic pulmonary fibrosis: a genome-wide association study. *Lancet Respir Med*. Jan 2023;11(1):65-73. doi:10.1016/S2213-2600(22)00251-X

57. Oldham JM, Allen RJ, Lorenzo-Salazar JM, et al. PCSK6 and Survival in Idiopathic Pulmonary Fibrosis. *Am J Respir Crit Care Med*. Jun 1 2023;207(11):1515-1524. doi:10.1164/rccm.202205-0845OC

58. Williams A, Shrine N, Naghra-van Gijzel H, et al. Genome-wide association study of susceptibility to hospitalised respiratory infections [version 2; peer review: 2 approved with reservations, 1 not approved]. *Wellcome Open Research*. 2023;6(290)doi:10.12688/wellcomeopenres.17230.2

59. Hobbs BD, Putman RK, Araki T, et al. Overlap of Genetic Risk between Interstitial Lung Abnormalities and Idiopathic Pulmonary Fibrosis. *Am J Respir Crit Care Med*. Dec 1 2019;200(11):1402-1413. doi:10.1164/rccm.201903-0511OC

60. Stelzer G, Rosen N, Plaschkes I, et al. The GeneCards Suite: From Gene Data Mining to Disease Genome Sequence Analyses. *Curr Protoc Bioinformatics*. Jun 20 2016;54:1 30 1-1 30 33. doi:10.1002/cpbi.5

61. Hamosh A, Scott AF, Amberger JS, Bocchini CA, McKusick VA. Online Mendelian Inheritance in Man (OMIM), a knowledgebase of human genes and genetic disorders. *Nucleic Acids Res*. Jan 1 2005;33(Database issue):D514-7. doi:10.1093/nar/gki033

62. Ochoa D, Hercules A, Carmona M, et al. The next-generation Open Targets Platform: reimagined, redesigned, rebuilt. *Nucleic Acids Res*. Jan 6 2023;51(D1):D1353-D1359. doi:10.1093/nar/gkac1046

63. Shibata R, Misonou H, Campomanes CR, et al. A fundamental role for KChIPs in determining the molecular properties and trafficking of Kv4.2 potassium channels. *J Biol Chem*. Sep 19 2003;278(38):36445-54. doi:10.1074/jbc.M306142200

64. Liu X, Hu C, Bao M, et al. Genome Wide Association Study Identifies L3MBTL4 as a Novel Susceptibility Gene for Hypertension. *Sci Rep*. Aug 2 2016;6:30811. doi:10.1038/srep30811

65. Hu C, Zuo K, Li K, et al. p38/JNK Is Required for the Proliferation and Phenotype Changes of Vascular Smooth Muscle Cells Induced by L3MBTL4 in Essential Hypertension. *Int J Hypertens*. 2020;2020:3123968. doi:10.1155/2020/3123968

66. Huang S. mTOR Signaling in Metabolism and Cancer. *Cells*. Oct 13 2020;9(10)doi:10.3390/cells9102278

67. Oh SJ. System-Wide Expression and Function of Olfactory Receptors in Mammals. *Genomics Inform*. Mar 2018;16(1):2-9. doi:10.5808/GI.2018.16.1.2

68. Serfozo P, Wysocki J, Gulua G, et al. Prolyl Oligopeptidase-Dependent Angiotensin II Conversion to Angiotensin-(1-7) in the circulation. *Hypertension*. Jan 2020;75(1):173-182. doi:10.1161/HYPERTENSIONAHA.119.14071

69. Vickers C, Hales P, Kaushik V, et al. Hydrolysis of biological peptides by human angiotensin-converting enzyme-related carboxypeptidase. *J Biol Chem*. Apr 26 2002;277(17):14838-43. doi:10.1074/jbc.M200581200

70. Kumar N, Yin C. The anti-inflammatory peptide Ac-SDKP: Synthesis, role in ACE inhibition, and its therapeutic potential in hypertension and cardiovascular diseases. *Pharmacol Res*. Aug 2018;134:268-279. doi:10.1016/j.phrs.2018.07.006

71. Garcia-Horsman JA. The role of prolyl oligopeptidase, understanding the puzzle. *Ann Transl Med*. Aug 2020;8(16):983. doi:10.21037/atm-20-3412

72. Krieger M, Roos A, Stendel C, et al. SIL1 mutations and clinical spectrum in patients with Marinesco-Sjogren syndrome. *Brain*. Dec 2013;136(Pt 12):3634-44. doi:10.1093/brain/awt283

73. Ramachandran N, Munteanu I, Wang P, et al. VMA21 deficiency prevents vacuolar ATPase assembly and causes autophagic vacuolar myopathy. *Acta Neuropathol*. Mar 2013;125(3):439-57. doi:10.1007/s00401-012-1073-6

74. Cannata Serio M, Graham LA, Ashikov A, et al. Mutations in the V-ATPase Assembly Factor VMA21 Cause a Congenital Disorder of Glycosylation With Autophagic Liver Disease. *Hepatology*. Dec 2020;72(6):1968-1986. doi:10.1002/hep.31218
